# Sex‐Specific Regulation of Glycemic Homeostasis by Theabrownin from Pu‐erh Tea

**DOI:** 10.1002/advs.202519337

**Published:** 2026-04-16

**Authors:** Yang Li, Junliang Kuang, Dan Zheng, Jieyi Wang, Kun Ge, Chun Qu, Xiaojiao Zheng, Zhenxing Ren, Fengjie Huang, Mingliang Zhao, Yimin Sun, Huiheng Zhang, Keke Ding, Xixi Xia, Yajun Tang, Lu Liu, Jian Zhou, Fei Gao, Xiaohui Ma, Yongquan Xu, Guoxiang Xie, Aihua Zhao, Wei Jia

**Affiliations:** ^1^ Center For Translational Medicine Shanghai Key Laboratory of Diabetes Mellitus Shanghai Sixth People's Hospital Affiliated to Shanghai Jiao Tong University School of Medicine Shanghai China; ^2^ Human Metabolomics Institute, Inc. Shenzhen China; ^3^ Department of Pharmacology and Pharmacy University of Hong Kong Hong Kong China; ^4^ Department of Endocrinology and Metabolism Shanghai Sixth People's Hospital Affiliated to Shanghai Jiao Tong University School of Medicine Shanghai China; ^5^ Tasly Research Institute Tianjin China; ^6^ School of Pharmacy, Health Science Center Ningbo University Ningbo China

**Keywords:** α‐glucosidase, hyperglycemia, MUC2, sex differences, Theabrownin

## Abstract

Theabrownin (TB) from Pu‐erh tea has demonstrated efficacy in alleviating hyperglycemia, yet its mechanism remains incompletely understood. In our study, we found that Pu‐erh tea or TB intervention effectively lowered postprandial blood glucose levels in females with normal and impaired glucose tolerance (IGT), as well as in hyperglycemic mouse models. Specifically, oral administration of Pu‐erh tea or TB led to the inhibition of small intestinal α‐glucosidase activity in a female‐specific manner. TB's inhibition of α‐glucosidase in vivo was significantly enhanced by mucin 2 (MUC2), a protein enriched in female intestinal mucin and upregulated by estradiol. Through noncovalent interactions, TB binds to MUC2 to form a complex, significantly boosting its inhibitory effect on α‐glucosidase in females. These findings reveal the sex‐specific molecular mechanism underlying the blood glucose‐lowering effects of TB from Pu‐erh tea, offering a promising intervention for maintaining glycemic balance in individuals with IGT or type 2 diabetes. Additionally, our study highlights the importance of considering sex differences in the judicious use of supplements or pharmacotherapy.

## Graphical abstract

Pu‐erh tea's key component, theabrownin (TB), lowers blood glucose in a sex‐specific manner. In females, estrogen boosts intestinal MUC2 production, which dramatically enhances TB's ability to inhibit the carbohydrate‐digesting enzyme α‐glucosidase. This discovery of the estrogen‐MUC2‐TB axis opens new avenues for personalized nutrition in hyperglycemic management.

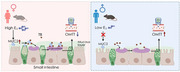

## 1 Introduction

Sex disparities exist in type 2 diabetes (T2D) concerning pathogenic factors, prevalence, and complications. Females have a lower likelihood of developing T2D than males but tend to experience more severe complications [1, 2, 3]. Factors including obesity, polycystic ovary syndrome (PCOS), gestational diabetes, menopausal hormonal changes, and psychosocial stress increase T2D risk in females [4, 5, 6, 7, 8]. Additionally, impaired glucose tolerance (IGT) is more prevalent in females than males [9, 10]. Females with T2D show a higher relative risk of cardiovascular disease (CVD), stroke, nerve injury, renal insufficiency, and depression compared to males [11, 12, 13, 14]. Despite their impact on glucose levels and overall health, these sex‐specific differences are often overlooked in the management of T2D. Recognizing and addressing these distinctions is crucial for the effective prevention and management of T2D, especially considering the lack of awareness among physicians and current guidelines.

Pu‐erh tea, derived from *Camellia sinensis var. assamica*, is a unique microbially fermented tea originating from southwestern China [15, 16]. Theabrownin (TB) is a macromolecular polymer of polyphenols formed during the fermentation of Pu‐erh tea. Our previous studies have shown TB to be the key bioactive component in mitigating hypercholesterolemia by regulating bile acid metabolism through reshaping the microbiota structure [17, 18]. In our earlier study, we noted a reduction in blood glucose levels in females compared to males following the Pu‐erh tea intervention, although these changes did not reach statistical significance across all participants. Other studies on the potential hypoglycemic effects of Pu‐erh tea or TB primarily focus on observational outcomes, potentially involving modulation of gut microbiota, improvement of insulin sensitivity, or in vitro inhibition of α‐glucosidase [19, 20, 21]. However, it remains unclear whether these effects vary by sex or how Pu‐erh tea or TB impacts α‐glucosidase activity in vivo. Alpha‐glucosidase, essential for postprandial blood glucose (PBG) elevation, is widely distributed in the small intestine. α‐glucosidase inhibitors (AGIs) are recognized for reducing PBG levels, potentially contributing to the prevention of T2D and CVD [22, 23, 24]. Chinese expert consensus documents have outlined these benefits in clinical studies involving patients with IGT and diabetes [25].

Building on our previous observations, we hypothesize that the glucose‐lowering effects of TB from Pu‐erh tea exhibit sex‐specificity. This study aims to test this hypothesis by investigating the effects of TB on glucose levels in female and male individuals with normal glucose tolerance (NGT) and impaired glucose tolerance (IGT), as well as in normal and hyperglycemic mouse models. In investigating the sex‐specific inhibition of α‐glucosidase, we will analyze the physiological variances based on sex in the mouse small intestine. This examination aims to elucidate how distinct small molecular metabolites and macromolecular proteins influence the glucose‐lowering effects of TB, the correlation between TB and critical differential metabolites and proteins, and the interaction between TB and proteins. Results generated from the study will highlight the significance of taking into consideration sex disparities when applying supplements or pharmaceuticals in a rational and effective manner.

## 2 Results

### 2.1 Pu‐erh Tea Lowered Postprandial Blood Glucose in Females but Not in Males

**FIGURE 1:**
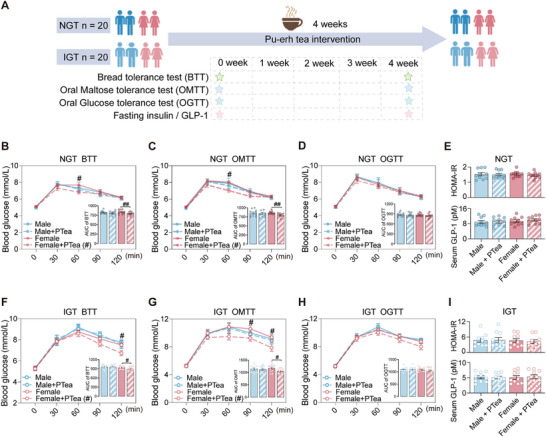
Pu‐erh tea regulated glucose homeostasis with sex differences in the NGT and IGT individuals. (A) Diagram of human study 1: After enrolment and adaptation period, the NGT (n = 20, 10 individuals of each sex) and IGT (n = 20, 10 individuals of each sex) individuals consumed Pu‐erh tea for 4 weeks. The assessments are displayed in the diagram. (B–E) (B) Blood glucose levels and AUC of BTT, (C) blood glucose levels and AUC of OMTT, (D) blood glucose levels and AUC of OGTT, and (E) HOMA‐IR and serum active GLP‐1 levels in the NGT individuals. (F–I) (F) Blood glucose levels and AUC of BTT, (G) blood glucose levels and AUC of OMTT, (H) blood glucose levels and AUC of OGTT, and (I) HOMA‐IR and serum active GLP‐1 levels in the IGT individuals. Data are presented as the mean ± SEM. ^#^ *p* < 0.05 and ^##^ *p* < 0.01 compared with the female groups with Pu‐er tea based on the Wilcoxon matched‐pairs signed rank test.

To investigate the glucose‐lowering effects of Pu‐erh tea, we enrolled 20 individuals clinically diagnosed with NGT and 20 individuals clinically diagnosed with IGT, matched for age and sex. Participants were instructed to drink 300 mL of instant Pu‐erh tea infusion twice daily at 50 mg/kg/day for 4 weeks, following the recommended daily intake of instant Pu‐erh tea in our previous study (Figure 1A; Table S1). At the end of the fourth week, all NGT individuals underwent the bread tolerance test (BTT). The results showed a decreasing trend in blood glucose levels at 60 min after Pu‐erh tea consumption, with a significant reduction in the area under the curve (AUC) of the BTT (Figure S1A) in NGT individuals. Further analysis showed that this decreasing trend was primarily observed in female individuals, while no significant changes were noted in male individuals (Figure 1B). Postprandial blood glucose levels are closely correlated with α‐glucosidase activity, insulin levels, and serum glucagon‐like peptide‐1 (GLP‐1) levels [26, 27]. Subsequent measurements of blood glucose levels were conducted using the oral maltose tolerance test (OMTT) and oral glucose tolerance test (OGTT) in NGT individuals, along with assessments of fasting serum insulin, homeostatic model assessment for insulin resistance (HOMA‐IR), and serum GLP‐1 levels. The results showed a similar trend of reduced blood glucose levels post‐intervention in female individuals during the OMTT (Figure 1C; Figure S1B). However, no significant changes were observed in the OGTT (Figure 1D; Figure S1C), fasting serum insulin (Figure S1D,E), HOMA‐IR (Figure 1E; Figure S1F), or serum GLP‐1 levels (Figure 1E; Figure S1G) following the Pu‐erh tea intervention.

Consistent with the findings in NGT individuals, Pu‐erh tea led to significantly lower PBG levels in IGT individuals, with average reductions of 0.5 mmol/L and 0.8 mmol/L at 120 min in the BTT and OMTT, respectively (Figure S1H,I). Similarly, the reduction in blood glucose levels was primarily observed in females rather than males (Figure 1F,G). Additionally, there were no significant changes in the OGTT (Figure S1J), although a slight improvement in blood glucose levels was noted among females (Figure 1H). Fasting serum insulin (Figure S1K,L), HOMA‐IR (Figure 1I; Figure S1M), and serum GLP‐1 levels (Figure 1I; Figure S1N) remained unchanged in IGT individuals after the 4‐week Pu‐erh tea intervention.

In summary, our findings suggest that Pu‐erh tea effectively reduces PBG levels in females but not in males. These effects may be related to α‐glucosidase activity rather than direct impacts on glucose utilization, insulin levels, insulin sensitivity, or serum GLP‐1 levels.

### 2.2 Pu‐erh Tea Inhibited Small Intestinal α‐Glucosidase Activity in a Sex‐Dependent Manner

To further verify the findings in humans, we assessed the glucose‐lowering effect of Pu‐erh tea in hyperglycemic mouse models. We administered Pu‐erh tea to mice with high‐fat diet (HFD) induced diabetes and those with HFD combined with streptozotocin (HFD+STZ) induced diabetes.

**FIGURE 2:**
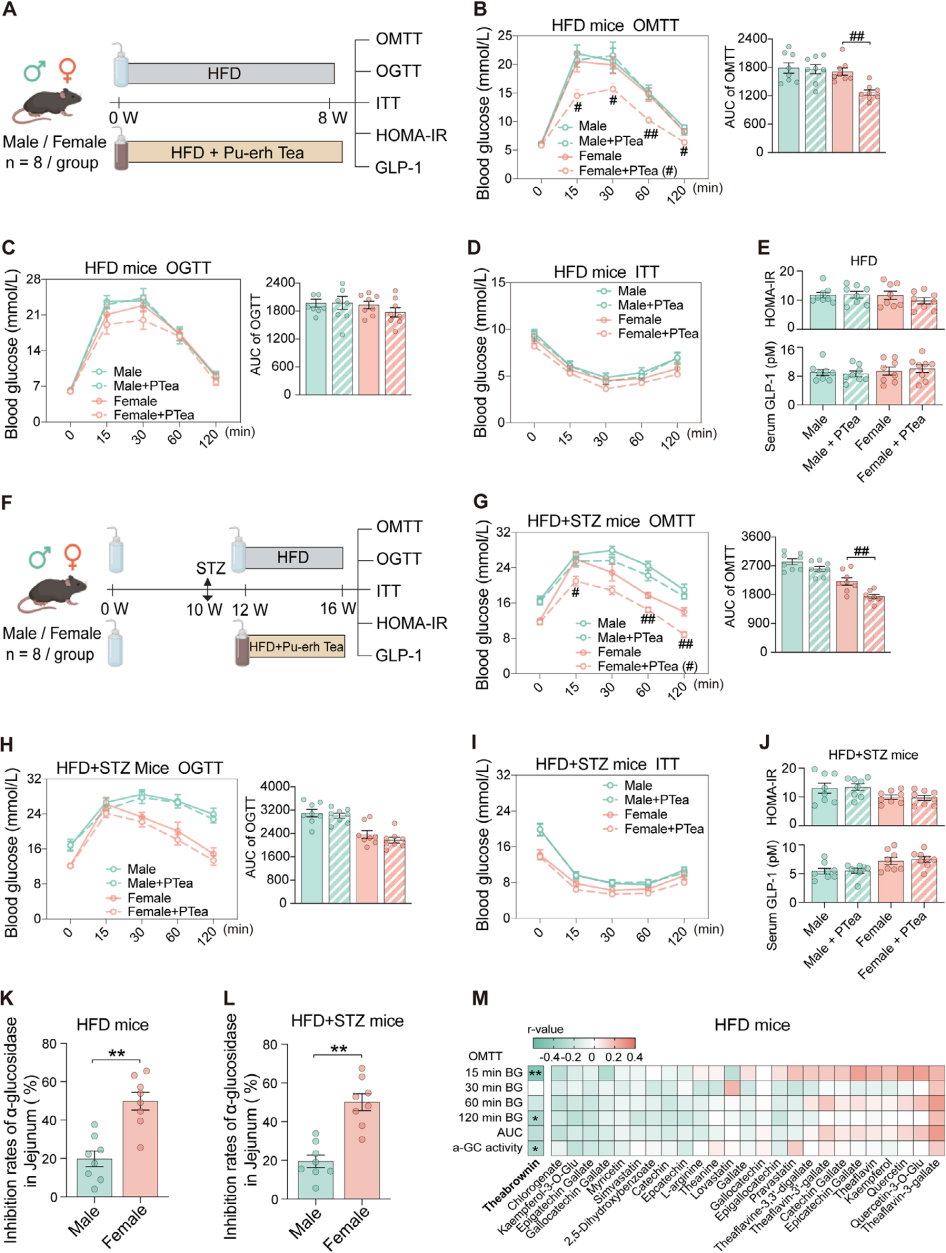
Pu‐erh tea inhibited small intestinal α‐glucosidase activity with sex differences in the HFD and HFD+STZ mice. (A) Animal experiment 1 in the HFD mice (HFD, HFD+PTea) (n = 16/group, 8 mice of each sex). (B–E) (B) Blood glucose levels and AUC of OMTT, (C) blood glucose levels and AUC of OGTT, (D) blood glucose levels of ITT, and (E) HOMA‐IR and serum active GLP‐1 levels in the HFD mice. (F) Animal experiment 2 in HFD+STZ mice (control, PTea) (n = 16/group, 8 mice of each sex). (G–J) (G) Blood glucose levels and AUC of OMTT, (H) blood glucose levels and AUC of OGTT, (I) blood glucose levels of ITT, and (J) HOMA‐IR and serum active GLP‐1 levels in the HFD+STZ mice. (K, L) Inhibition rates of α‐glucosidase in the jejunum of the HFD (K) and HFD+STZ mice (L), respectively. (M) Heatmaps of Spearman correlation coefficients of blood glucose (BG) levels in the OMTT, α‐glucosidase, with Pu‐erh tea components in HFD mice. The color of the cells indicates the Spearman correlation coefficients. Data are shown as mean ± SEM. ^#^ *p* < 0.05 and ^##^ *p* < 0.01 in (B) and (G) compared with the female groups with Pu‐er tea, based on the Mann–Whitney U test; ^**^ *p* < 0.01 in (K) and (L) compared with the male groups based on the Mann–Whitney U test; ^*^ *p* < 0.05 and ^**^ *p* < 0.01 in (M) compared with the male groups, based on the Spearman correlation. r: Spearman correlation coefficient.

In the HFD model, mice were given an instant Pu‐erh tea infusion at a concentration of 3 mg/mL, equivalent to 450 mg/kg/day body weight (9 times the equivalent human dose, calculated based on the body surface area of humans and mice) via their water bottles ad libitum for 8 consecutive weeks (Figure 2A). We have found that Pu‐erh tea administration did not significantly alter body weight, food or water consumption in either male or female mice compared to control groups (Figure S2A–C).

Consistent with our findings from human IGT individuals in the OMTT, Pu‐erh tea significantly reduced PBG levels and AUC in the mice (Figure S2D), with more pronounced effects observed in female mice. Notably, blood glucose levels in female mice even returned to normal levels at 120 min (Figure 2B). However, no significant differences were observed in the OGTT or insulin tolerance test (ITT) (Figure S2E,F), although there was an improvement noted in the female intervention group (Figure 2C,D). Fasting serum insulin (Figure S2G,H), the HOMA‐IR (Figure 2E; Figure S2I), and serum GLP‐1 levels (Figure 2E; Figure S2J) remained unchanged.

In the HFD+STZ diabetic mouse model, mice were treated with the same doses of instant Pu‐erh tea infusion used in the HFD model for 4 consecutive weeks (Figure 2F). The result also showed no significant changes in body weight, dietary, and water intake compared to Pu‐erh tea treatment with control groups (Figure S3A–C). Similar to the HFD model, Pu‐erh tea improved glucose levels in the OMTT (Figure S3D), particularly in female mice (Figure 2G). However, there were no significant changes in the OGTT (Figure 2H; Figure S3E), ITT (Figure 2I; Figure S3F), fasting serum insulin (Figure S3G,H), HOMA‐IR (Figure 2J; Figure S3I), and serum GLP‐1 levels (Figure 2J; Figure S3J) in this model.

To verify that Pu‐erh tea improves PBG levels by inhibiting α‐glucosidase in the small intestine, we measured the mucosal enzymatic activity across different physiological segments of the small intestine in both mouse models. Our results showed that α‐glucosidase inhibition was significantly greater in female mice treated with Pu‐erh tea compared to male mice in the duodenum (Figure S3K,L), jejunum (Figure 2K,L), and ileum (Figure S3M,N).

To pinpoint the specific compounds in instant Pu‐erh tea responsible for its blood glucose‐lowering effects and inhibition of α‐glucosidase activity, we conducted a quantitative analysis of tea polyphenols and tea pigments in the small intestinal contents of mice treated with HFD and Pu‐erh tea using quadrupole time‐of‐flight mass spectrometry (UPLC/QTOFMS). Among the various components present in the tea, TB demonstrated the most significant and negative correlation with blood glucose levels at 15 and 120 min during the OMTT, as well as with the α‐glucosidase activity in the small intestine (Figure 2M).

### 2.3 TB Exhibited a More Pronounced Inhibition of α‐Glucosidase in Females Compared to Males

**FIGURE 3:**
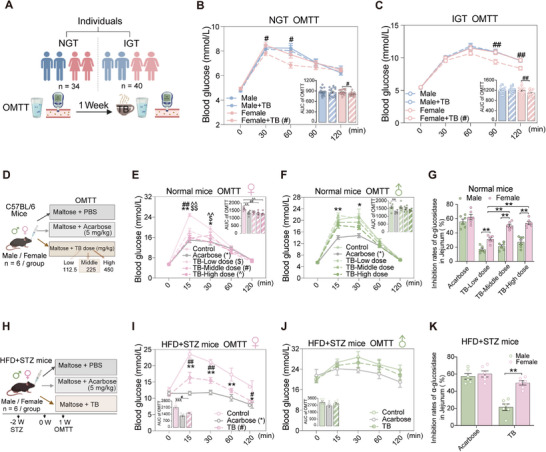
Theabrownin inhibited α‐glucosidase with sex differences. (A) Diagram of human study 2: After enrolment and adaptation period, the NGT (n = 34, 17 individuals of each sex) and IGT (n = 40, 20 individuals of each sex) individuals accepted the assessments displayed in the diagram. (B, C) Blood glucose levels and AUC of OMTT in the NGT individuals (B) and IGT individuals (C). (D) Animal experiment 3 in the normal mice (control, Acarbose, TB‐Low dose, TB‐Middle dose, TB‐High dose) (n = 12/group, 6 mice of each sex). (E, F) Blood glucose levels and AUC of OMTT in the female (E) and male mice (F). (G) Inhibition rates of α‐glucosidase in the jejunum of the male and female mice. (H) Animal experiment 4 in the HFD+STZ mice (control, Acarbose, TB) (n = 12/group, 6 mice of each sex). (I, J) Blood glucose levels and AUC of OMTT in the female (I) and male mice (J). (K) Inhibition rates of α‐glucosidase in the jejunum of the male and female mice. Data are shown as mean ± SEM. ^#^ *p* < 0.05 and ^##^ *p* < 0.01 in (B) and (C) compared with the female individuals, based on the Wilcoxon matched‐pairs signed rank test; In (E, F) and (I, J), ^*^, ^#^, ^$^ *p* < 0.05, ^**^, ^$$, ##, ^^^ *p* < 0.01 compared with control groups based on the Kruskal–Wallis test with Benjamini Hochberg adjustment; ^**^ *p* < 0.01 in (G) and (K) compared with the male groups, based on the Mann–Whitney U test.

Given the correlation between TB and the reduction of blood glucose levels in the OMTT, as well as the inhibition of α‐glucosidase activity, we postulated that TB played a pivotal role in these outcomes. To test this hypothesis, we enrolled 34 individuals clinically diagnosed with NGT and 40 individuals diagnosed with IGT, who were age‐ and sex‐matched (Figure 3A, Table S2). The study aimed to examine the impact of TB on blood glucose levels during the OMTT.

All individuals underwent two OMTTs, with the second test conducted one week after a single oral administration of TB at a dose of 25 mg/kg dissolved in 300 mL of boiling water. The results showed a significant reduction in glucose levels at 30 and 60 min in NGT females (Figure 3B) and at 90 and 120 min in IGT females (Figure 3C) after TB treatment. However, these effects were not observed in NGT or IGT males, although there was a decreasing trend in all individuals after TB administration (Figure S4A,B).

To investigate whether the impact of TB on PBG reduction is due to sex‐dependent inhibitory effects on α‐glucosidase activity, we first administered a single dose of TB at low (112.5 mg/kg), medium (225 mg/kg), and high (450 mg/kg) to normal mice, with Acarbose as a positive control. We then evaluated its effects on PBG levels through the OMTT (Figure 3D). The glucose‐lowering effects of TB were more pronounced with increasing doses of TB, particularly in female mice (Figure 3E; Figure S4C), while no significant effects were observed in male mice, even at the highest dose (Figure 3F).

Subsequently, we measured the activities of α‐glucosidase in the duodenum, jejunum, and ileum of the mice from each group. The results showed that the inhibition of intestinal α‐glucosidase activity increased with TB doses (Figure S4D–F), with a significant inhibition observed in female mice but not in male mice (Figure 3G; Figure S4G,H). Notably, the high dose of TB in female mice exhibited a similar inhibitory effect to that of Acarbose. Furthermore, when assessing the effects of TB on PBG improvement in diabetic model mice (Figure 3H), female mice showed a more significant improvement in glucose levels (Figure 3I,J; Figure S4I) and a pronounced inhibition of α‐glucosidase activity in various segments of the small intestine compared to male mice (Figure 3K; Figure S4J–N).

### 2.4 Estradiol Enhanced TB's Inhibition of α‐Glucosidase in vivo

**FIGURE 4:**
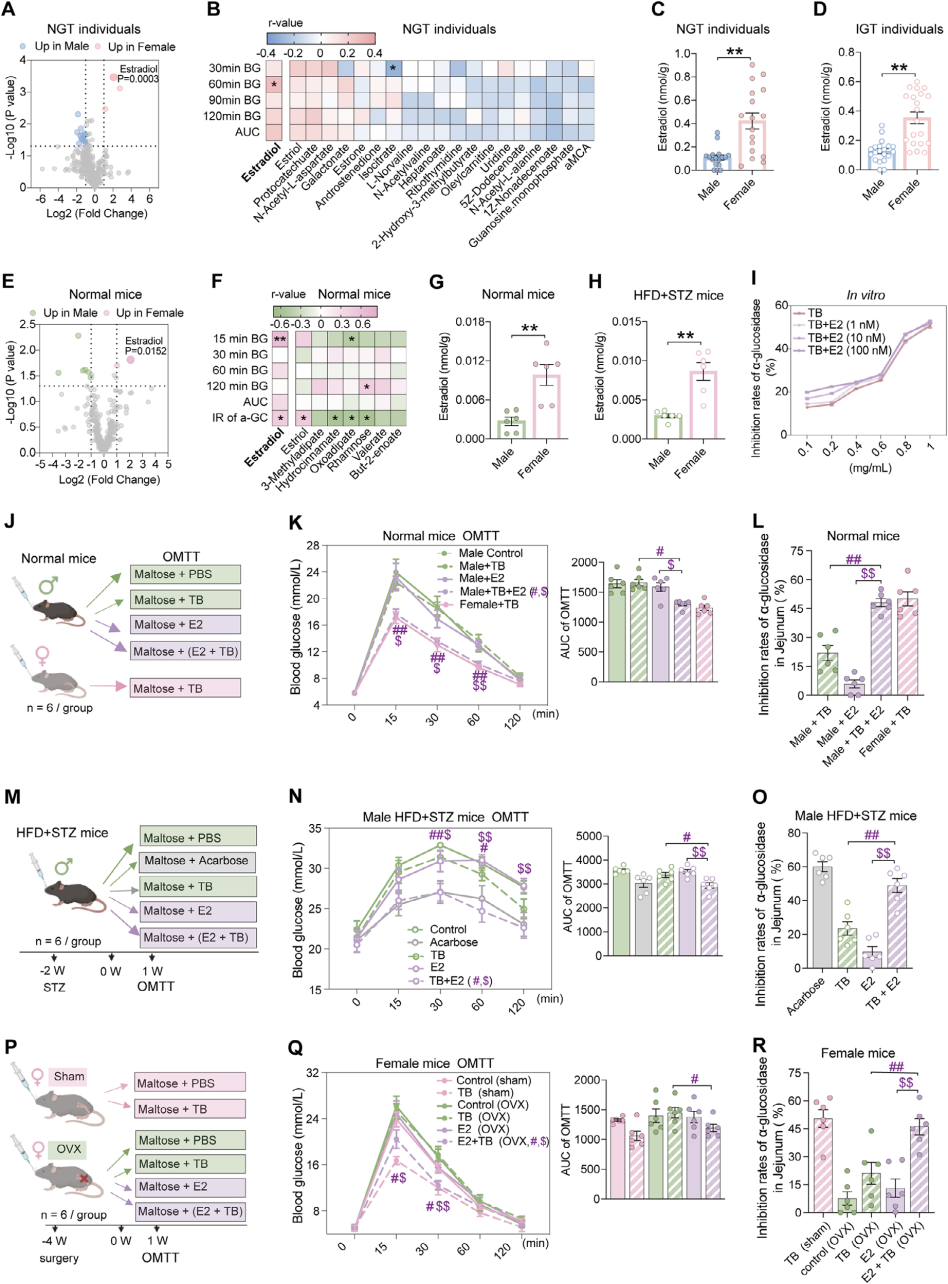
Estradiol enhanced TB's Inhibition of α‐glucosidase in vivo. (A) Differential metabolites abundance from feces in 17 male vs 17 female NGT individuals, highlighted with a volcano plot. The blue dots indicate the metabolites are up in the male, and the red dots indicate the metabolites are up in the female. (B) Heatmaps of Pearson correlation coefficients of decreased blood glucose (BG) levels in the OMTT with significantly differential metabolite abundance in the NGT individuals. The color of the cells indicates the Pearson correlation coefficients. (C, D) The levels of fecal estradiol in the NGT (C) and IGT individuals (D). (E) Differential metabolite abundance from small intestine content in 6 normal male vs 6 female mice, highlighted with a volcano plot. The green dots indicate the metabolites are up in the male, and the pink dots indicate the metabolites are up in the female. (F) Heatmaps of Pearson correlation coefficients of decreased BG levels in the OMTT and α‐glucosidase with significantly differential metabolite abundance in the normal mice. The color of the cells indicates the Pearson correlation coefficients. (G, H) The levels of estradiol of small intestine content in the normal (G) and HFD+STZ mice (H). (I) Inhibitory effects of TB, TB+Estradiol (E2,1 nM), TB+E2 (10 nM), and TB+E2 (100 nM) on α‐glucosidase in vitro. (J) Animal experiment 5 in the male normal mice with E2 (Male control, Male TB, Male E2, Male E2+TB, Female TB) (n = 6/group). (K, L) (K) Blood glucose levels and AUC of OMTT and (L) inhibition rates of α‐glucosidase in the jejunum of the normal mice. (M) Animal experiment 6 in the male mice with E2 (Control, Acarbose, TB, E2, E2+TB) (n = 6/group). (N, O) (N) Blood glucose levels and AUC of OMTT and (O) inhibition rates of α‐glucosidase in the jejunum of the male mice with E2. (P) Animal experiment 7 in the female mice of ovariectomy (Sham control, Sham TB, OVX control, OVX TB, OVX E2, OVX E2+TB) (n = 6/group). (Q, R) (Q) Blood glucose levels and AUC of OMTT and (R) inhibition rates of α‐glucosidase in the jejunum of the female mice after ovariectomy. Data are shown as mean ± SEM. ^*^ *p* < 0.05 and ^**^ *p* < 0.01 in (B) and (F) based on the Pearson correlation. ^**^ *p* < 0.01 in (C, D) and (G, H) compared with male groups based on the Mann–Whitney U test. In (K–R), ^#^ *p* < 0.05 and ^##^ *p* < 0.01 compared with male TB and OVX TB groups, respectively; ^$^ *p* < 0.05 and ^$$^ *p* < 0.01 and compared with male E2 and OVX E2 groups, respectively, based on the Kruskal–Wallis test with Benjamini Hochberg adjustment.

To investigate the molecular mechanism underlying the sex‐dependent effects of TB on α‐glucosidase inhibition, we first evaluated the expressions of sucrase‐isomaltase (*Si*) and maltase‐glucoamylase (*Mgam*) [28, 29, 30], two major α‐glucosidase genes in mice. Our results showed no sex‐specific differences in the expressions of these two genes (Figure S5A–C), both before and after TB intervention (Figure S5D–F). Additionally, there were no significant variations in α‐glucosidase activity across different segments of the small intestine between female and male mice (Figure S5G–I). However, significant sex‐specific differences were observed in fecal metabolites of NGT individuals, as shown in partial least‐squares discriminant analysis (PLS‐DA) (Figure S6A). Estradiol emerged as the most upregulated metabolite in the fecal metabolites analysis (the volcano plot, Figure 4A) and was positively correlated with decreased postprandial glucose levels pre‐ and post‐TB intervention (Figure 4B). The concentration of estradiol in female feces was approximately four times higher than in males, across both NGT and IGT individuals (Figure 4C,D).

Consistent with human findings, significant sex‐specific differences in metabolites were also observed in the small intestinal content of mice, as shown in the PLS‐DA (Figure S6B). Estradiol was identified as the most upregulated metabolite in female mice (Figure 4E) and positively correlated with the reduction in PBG levels and the inhibition of α‐glucosidase activity, both pre‐ and post‐TB intervention (Figure 4F). Similarly, estradiol levels were significantly higher in females compared to males, in normal mice as well as those with HFD+STZ‐induced diabetes (Figure 4G,H). These results suggest that estradiol is a key sex‐specific metabolite that may contribute to the differential effects of TB.

To explore the direct impact of estradiol on TB's inhibition of α‐glucosidase activity, in vitro experiments were conducted using estradiol alone and in combination with TB. The results indicated that estradiol alone (Figure S6C) or in combination with TB (Figure 4I) did not enhance α‐glucosidase activity. However, in male normal mice, the combination of estradiol and TB during TB administration led to a significant reduction in glucose levels in the OMTT (Figure 4J,K) and increased inhibition of α‐glucosidase activity compared to estradiol alone (Figure 4L; Figure S6D,E).

We then administered estradiol to male diabetic mice (Figure 4M) and observed similar reductions in glucose levels (Figure 4N) and inhibition of α‐glucosidase in the group receiving a combination of estradiol and TB (Figure 4O; Figure S6F,G). To confirm the critical role of estradiol in TB‐mediated α‐glucosidase inhibition, we conducted additional experiments on ovariectomized (OVX) female mice. The mice were treated with estradiol alone, TB alone, and a combination of TB and estradiol, and the changes in glucose levels during the OMTT were assessed (Figure 4P). The group receiving a combination of TB and estradiol showed a significant reduction in glucose levels, similar to those in the control group, whereas no effects were observed in the groups treated with estradiol or TB alone in the OVX mice (Figure 4Q). The inhibition of α‐glucosidase activity confirmed the changes observed in glucose levels (Figure 4R; Figure S6H,I).

These findings show that estradiol significantly boosts TB's inhibitory effect on α‐glucosidase activity. However, such enhancement may not be a direct synergistic effect of estradiol and TB. It is plausible that there is a substance under the regulation of estradiol that interacts with TB to facilitate the inhibition of α‐glucosidase.

### 2.5 MUC2 Expression in the Small Intestine was Higher in Female Mice Compared to Male Mice

**FIGURE 5:**
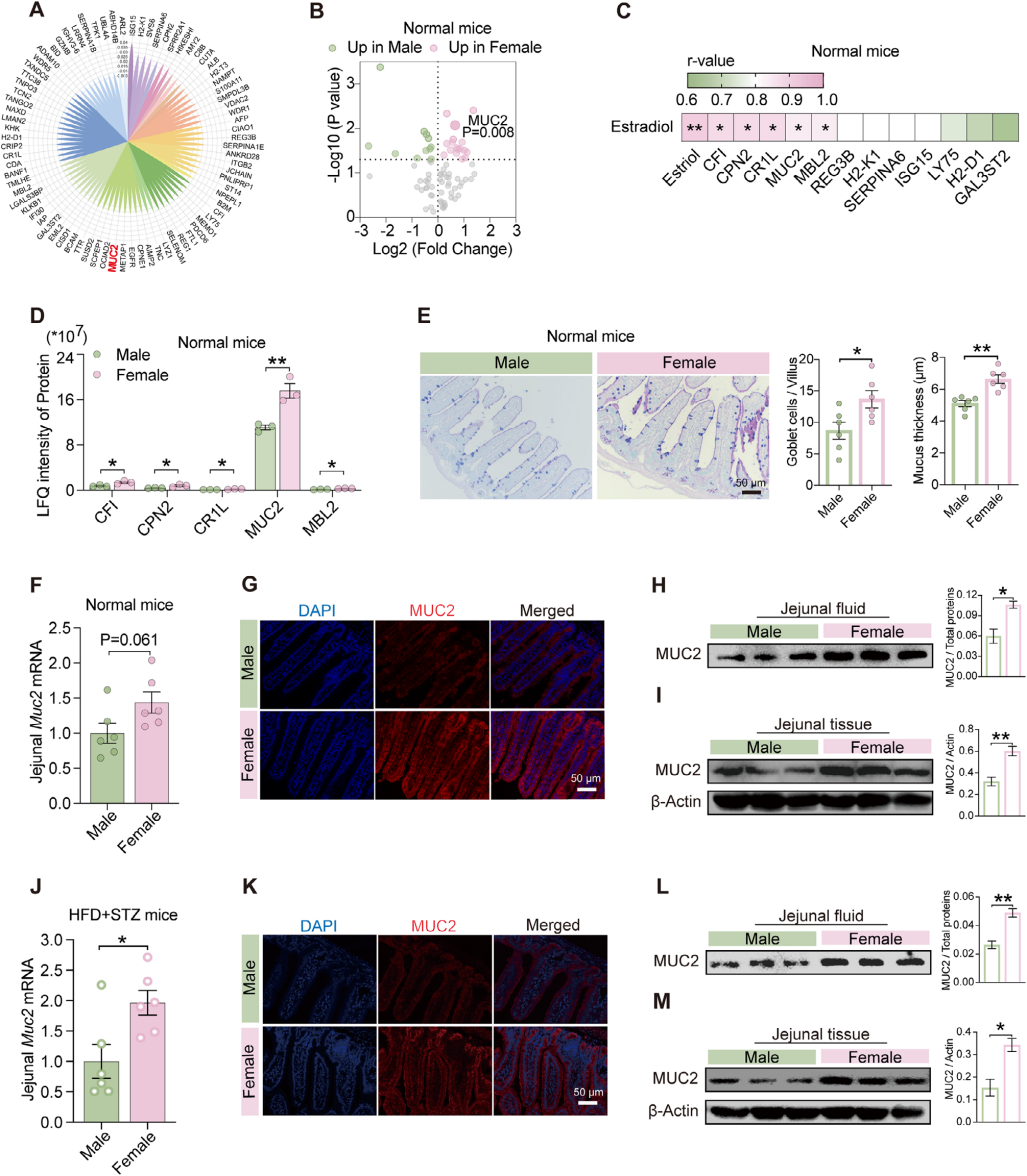
MUC2 was expressed with sex differences in the small intestines of mice. (A) The Petal diagram is based on the results of the Random Forest feature selection. The vertical axis represents the Mean Decrease Gini (MDG) values. (B) Differential proteins from the jejunal fluid in 3 normal male vs 3 female mice, highlighted with a volcano plot. The green dots indicate the proteins are up in the male, and the pink dots indicate the proteins are up in the female. (C) Heatmaps of Spearman correlation coefficients of significantly upregulated proteins and metabolites with estradiol in the female mice. The color of the cells indicates the Spearman correlation coefficients. (D) LFQ intensity of proteins in the jejunal fluid of the normal mice. (E) Alcian blue and periodic acid‐schiff stain (AB‐PAS) showing representative images of each group (left) and statistical analysis (right) of PAS‐positive Goblet cells and mucosal thickness in the normal mice (n = 6/group); scale bars, 50 µm. (F, G) The relative mRNA levels (F) and immunofluorescence staining of MUC2 (G) in the jejunal tissues of the normal mice (n = 3/group). (H, I) The protein expression of MUC2 in the jejunal fluid (H) and tissues (I) of the normal mice (n = 3/group). (J, K) The relative mRNA levels (J) and immunofluorescence staining of MUC2 (K) in the jejunal tissues of the HFD+STZ mice (n = 3/group). (L, M) The protein expression of MUC2 in the jejunal fluid (L) and tissues (M) of the HFD+STZ mice (n = 3/group). Data are presented as the mean ± SEM. ^*^ *p* < 0.05 and ^**^ *p* < 0.01 compared with male groups based on the Mann–Whitney U test.

To investigate the substance regulated by estradiol that may directly impact α‐glucosidase activity in a sex‐dependent manner through TB, we initiated a proteomic analysis of macromolecular substances in the small intestinal mucus of normal mice. This analysis identified 391 extracellularly secreted proteins with Label‐Free Quantitation (LFQ) intensity greater than 10E^6^. Among the top 20% of the most contributive sex differences identified by random forest multivariate analysis, 28 proteins exhibited sex‐based variances (Figure 5A,B). A correlation analysis between estradiol and the significantly upregulated metabolites and proteins in females revealed that estriol and five proteins were significantly positively correlated with estradiol (Figure 5C).

No correlation was found between estriol and decreased PBG levels in NGT individuals. Estriol, either alone (Figure S7A) or in combination with TB (Figure S7B), did not enhance α‐glucosidase activity in vitro, indicating no direct effect of estriol and TB on the improved glucose levels and inhibition of α‐glucosidase. As the LFQ intensity of MUC2 was notably higher than that of the other proteins analyzed (Figure 5D), MUC2 was identified as the most crucial sex‐differentiated protein for further investigation.

MUC2 is a mucin protein produced by intestinal epithelial goblet cells that is secreted into the extracellular space, where it combines with water and inorganic salts to form the mucus layer [31, 32]. Examination of the jejunum in normal female mice revealed an increased number of goblet cells and a thicker mucus layer compared to male mice, as demonstrated by intestinal PAS staining (Figure 5E). These findings were corroborated by the upregulation of MUC2 mRNA and protein expression in the jejunum of normal mice. The expression of MUC2 in jejunal tissues was found to be elevated in females based on qPCR and immunofluorescence analysis (Figure 5F,G). Furthermore, the protein expression of MUC2 in jejunal fluid and tissues was approximately 1.5 times higher in female mice than in male mice (Figure 5H,I; Figure S8A). Similar results were observed in HFD+STZ diabetic mice, indicating that the mRNA and protein levels of MUC2 in the jejunum remained consistent with those observed in normal mice (Figure 5J–M; Figure S8B).

### 2.6 Estradiol‐Regulated MUC2 Enhanced α‐Glucosidase Inhibition by TB

**FIGURE 6:**
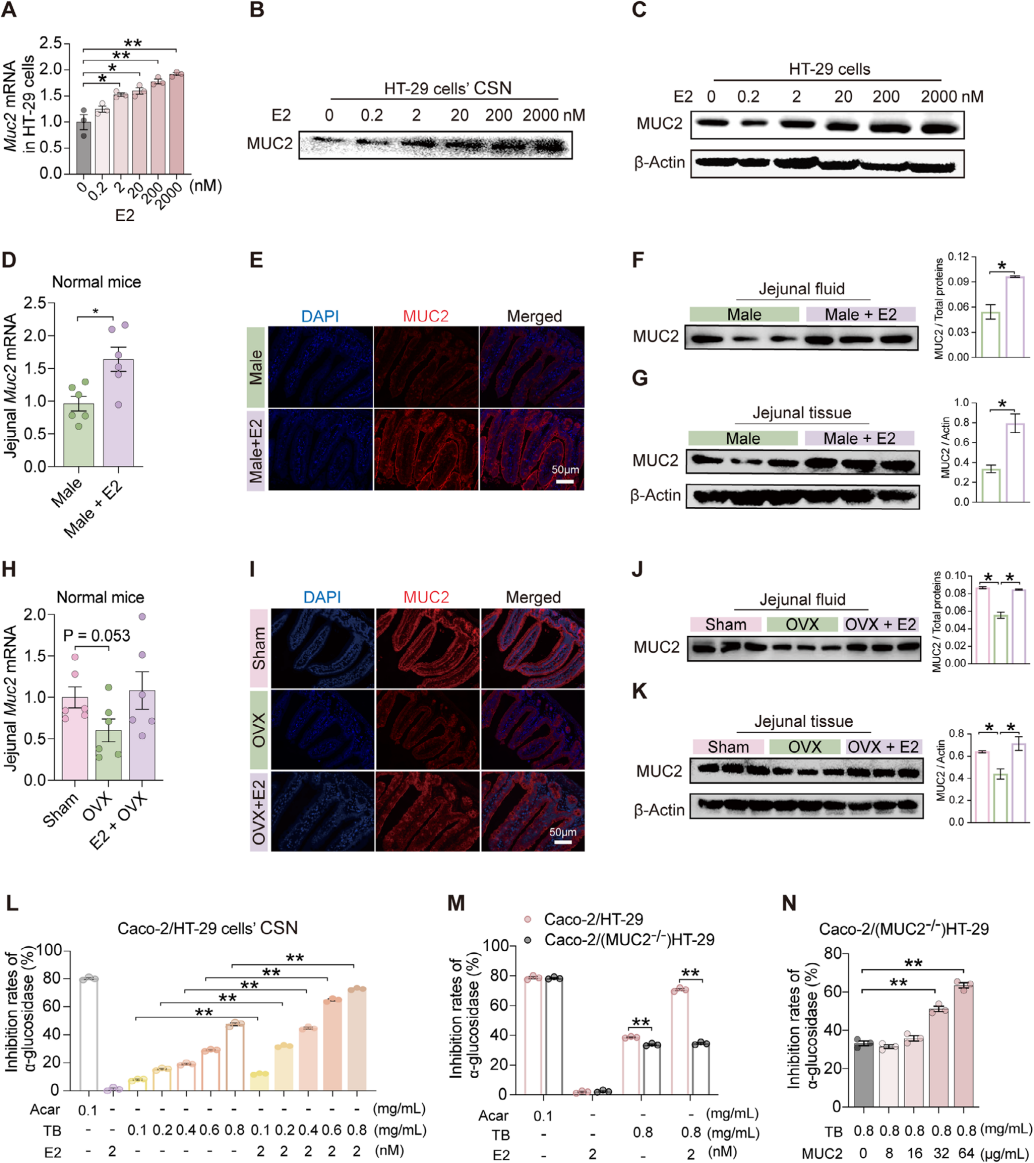
TB combination with estradiol‐regulated MUC2 enhanced α‐glucosidase inhibition. (A) The relative mRNA levels of *Muc2* in HT‐19 cells. (B, C) The protein expression of MUC2 in the cell supernatant (CSN) (B) and in HT‐19 cells (C). (D, E) The relative mRNA levels (D) and immunofluorescence staining (E) of MUC2 in the jejunal tissues of the male normal mice (control and E2 groups). (F, G) The protein expression of MUC2 in the jejunal fluid (F) and tissues (G) of the male normal mice (control and E2 groups). (H, I) The relative mRNA levels (H) and immunofluorescence staining (I) of MUC2 in the jejunal tissues of the female mice (Sham, OVX, and OVX+E2 groups). (J, K) The protein expression of MUC2 in the jejunal fluid (J) and tissues (K) of the female mice (Sham, OVX, and OVX+E2 groups). (L) Inhibitory effects of Acarbose (Acar), E2, TB of different concentration gradients, and TB plus E2 of different concentration gradients on α‐glucosidase in Caco‐2/HT‐29 cells with CSNs. (M) Inhibitory effects of Acarbose (Acar), E2, TB and TB plus E2 on α‐glucosidase in Caco‐2/HT‐29 and Caco‐2/(MUC2‐/‐) HT‐29 cells. (N) Inhibitory effects of TB and TB plus MUC2 of different concentration gradients on α‐glucosidase in Caco‐2/(MUC2‐/‐) HT‐29 cells. Data are presented as the mean ± SEM. ^*^ *p* < 0.05 and ^**^ *p* < 0.01 compared between groups, based on the Kruskal–Wallis test with Benjamini Hochberg adjustment and the Mann–Whitney U test.

To investigate the relationship between estradiol and MUC2 expression, gradient concentrations of estradiol were added to HT‐29 cells to assess MUC2 expression. The results demonstrated an increase in *Muc2* mRNA levels with higher concentrations of estradiol (Figure 6A). Western blot analysis of MUC2 secreted into the cell supernatant showed a significant upregulation with escalating estradiol concentrations (Figure 6B), while intracellular MUC2 protein levels did not exhibit a significant increase with increasing estradiol concentrations (Figure 6C).

To further confirm the regulation of MUC2 expression by estradiol in animal jejunal tissues, MUC2 expression was quantified in male mice supplemented with estradiol. Significant upregulation of MUC2 expression was observed in the jejunal tissues of male mice receiving estradiol supplementation compared to normal male mice, as evidenced by qPCR and immunofluorescence analysis (Figure 6D,E). The protein expression of MUC2 in the jejunal fluid of male mice supplemented with estradiol was also increased (Figure 6F; Figure S8C), with MUC2 protein levels in the jejunal tissues (Figure 6G) consistent with those observed in the jejunal fluid. To further validate the dose‐dependent manner of MUC2 expression in response to estradiol (E2), we administered low‐ (0.6 ng/mouse), medium‐ (3 ng/mouse), and high‐dose (6 ng/mouse) E2 to normal male mice. The jejunal tissues were collected for determining MUC2 qPCR and immunofluorescence staining in the same way. The results demonstrated a dose‐dependent upregulation of MUC2 expression in response to increasing estradiol concentrations (Figure S9A,B).

Subsequent investigations focused on the critical role of estradiol in MUC2 expression. MUC2 expression in jejunal tissues was reduced in ovariectomized (OVX) female mice, as indicated by qPCR and immunofluorescence analysis (Figure 6H,I). Protein expression of MUC2 in the jejunal fluid and tissues was also decreased in OVX female mice, but supplementation with physiological doses of estradiol restored MUC2 expression to levels comparable to those in the control group (Figure 6J,K; Figure S8D). To further investigate whether MUC2 expression correlates with the estrous cycle, we successively stained the vaginal cells with modified Giemsa solution in the mouse to track the female mouse estrous cycle (Figure S9C). We found that vaginal cells displayed obvious characteristics during proestrus, estrus, metestrus, and diestrus, consistent with the report [33]. The jejunal tissues were then collected for qPCR and immunofluorescence staining of MUC2 in the 4 estrus phases. The results revealed that female mice exhibited consistently higher *Muc2* mRNA expression levels across all estrous cycle phases compared to male mice, with statistically significant differences particularly during proestrus, estrus, and metestrus phases (Figure S9D). These findings were consistent with the changes in immunofluorescence staining (Figure S9E).

Further experiments explored whether increased MUC2 levels could enhance TB's ability to inhibit α‐glucosidase in differentiated Caco‐2 cells and Caco‐2 cells cultured with HT‐29 cells. The results showed that estradiol significantly boosted MUC2 protein content in the supernatant of co‐cultured cells, with no detectable secretion of MUC2 in the supernatant of Caco‐2 cells alone (Figure S10A). Gradient concentrations of TB and estradiol were then assessed for their effects on α‐glucosidase inhibition in differentiated Caco‐2 cells, with 100 µg/mL Acarbose serving as a positive control. The highest inhibition rate achieved by TB was approximately 40% with increasing concentrations, while estradiol did not significantly impact α‐glucosidase activity (Figure S10B).

Upon adding gradient concentrations of TB to the supernatants of co‐cultured cells with and without MUC2, the inhibitory effect of TB on α‐glucosidase was significantly enhanced under estradiol intervention, resulting in about 78% inhibition at a TB concentration of 0.8 mg/mL, comparable to the 80% inhibition observed with Acarbose (Figure 6L). However, enzymatic activity remained similar to the group without estradiol intervention, even in the presence of estradiol, when the supernatants were replaced with Phosphate Buffer Saline (Figure S10C).

To confirm the role of MUC2 in enhancing TB's inhibition of α‐glucosidase, comparisons were made between MUC2‐knockout HT‐29 and MUC2‐knockout HT‐29 co‐cultured with Caco‐2 cells (Figure S10D). TB did not exhibit a significant increase in enzyme inhibition rate under estradiol intervention in the MUC2‐knockout HT‐29 co‐cultured with Caco‐2 cells (Figure 6M). However, when MUC2 was reintroduced into the supernatant, a dose‐dependent inhibitory effect was restored to levels comparable to those in the normal co‐cultured cells (Figure 6N).

### 2.7 The Binding of TB to MUC2 Displayed Non‐Competitive, Reversible Inhibition of α‐Glucosidase

**FIGURE 7:**
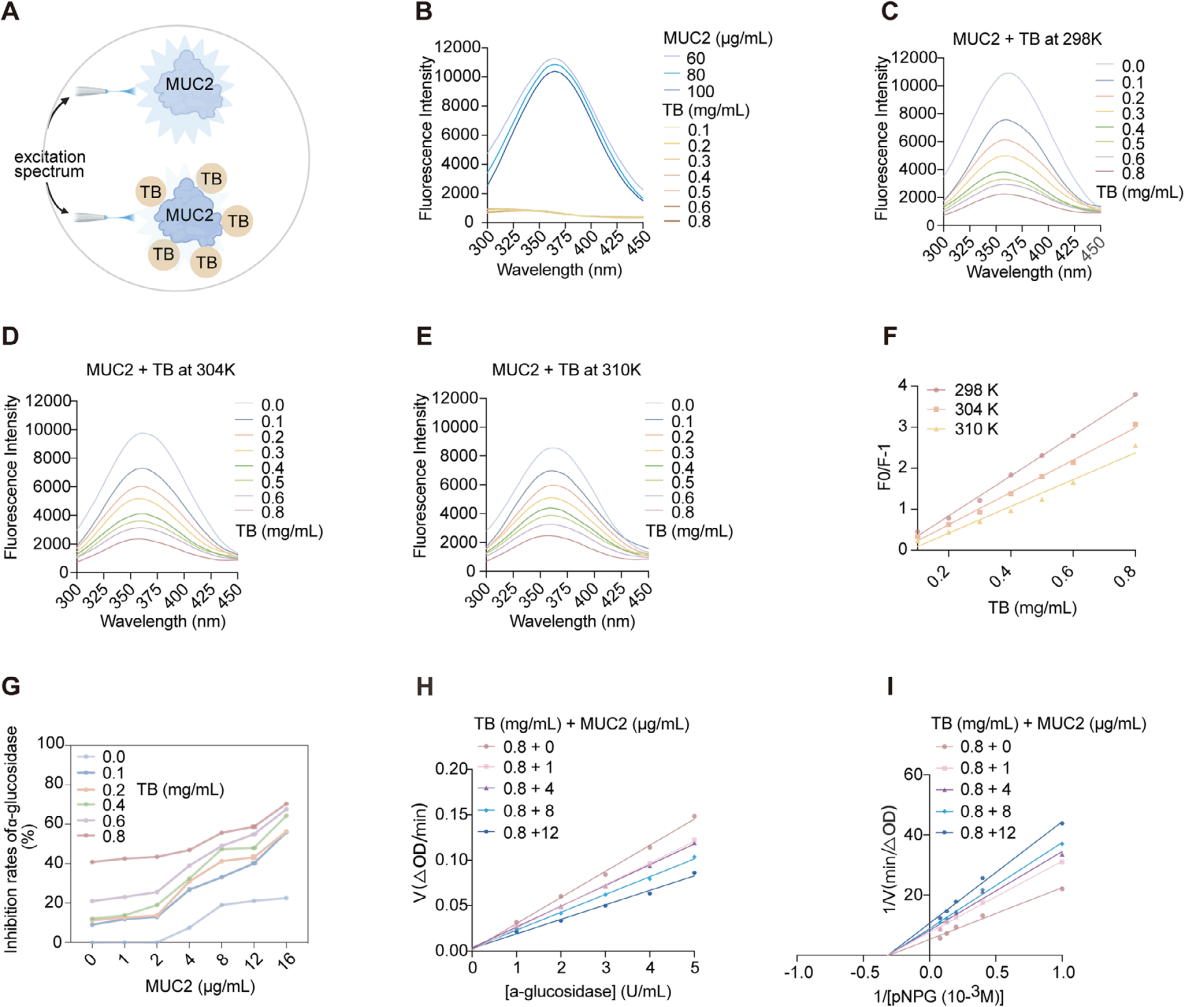
TB‐MUC2 interaction and inhibition kinetics on α‐glucosidase in vitro. (A) Illustration of fluorescence quenching of TB to MUC2. (B) Fluorescence spectra of TB and MUC2 at different concentrations. (C–E) Fluorescence spectra of MUC2 (80 µg/mL) at different concentrations of TB at 298 K (C), 304 K (D), and 310 K (E). (F) The plots of Stern–Volmer for the quenching of MUC2 by TB (0 – 0.8 mg/mL) at 298 K, 304 K, and 310 K, respectively. (G) Inhibitory effects of TB, MUC2, and TB plus MUC2 at different concentrations on α‐glucosidase. (H) Inhibition types of TB (0.8 mg/mL) plus MUC2 of different concentrations on α‐glucosidase. (I) Lineweaver–Burk plots of the reaction of α‐glucosidase in the presence of TB (0.8 mg/mL) plus MUC2 of different concentrations.

To investigate the binding of TB to MUC2, we utilized a fluorescence quenching technique to characterize the hydrogen bond binding between proteins or between proteins and small molecules (Figure 7A). MUC2 exhibited strong fluorescence absorption at various concentrations, while TB displayed negligible fluorescence intensity at concentrations ranging from 0.1 to 0.8 mg/mL (Figure 7B). However, the fluorescence intensities of MUC2 were progressively quenched upon the addition of TB at temperatures of 298, 304, and 310 K (Figure 7C–E).

To determine whether the quenching effect between TB and MUC2 was dynamic or static, we calculated the slope of the Stern‐Volmer equation at three temperatures [34]. The slopes decreased with increasing temperature, indicating that TB induced static quenching of MUC2 (Figure 7F). The enhanced inhibitory effects of the TB‐MUC2 combination on α‐glucosidase were evidenced across a range of TB concentrations with increasing concentrations of MUC2 in vitro (Figure 7G).

To delineate the inhibitory effects of the TB‐MUC2 combination on α‐glucosidase kinetics, we examined the kinetic line of α‐glucosidase inhibition by TB in the presence and absence of MUC2 across the origin. The results indicated that the TB‐MUC2 combination displayed reversible inhibition of α‐glucosidase by the line intersecting the origin, with a decreasing slope as the concentration of the combination increased (Figure 7H). Furthermore, this effect was characterized as non‐competitive inhibition based on the Lineweaver–Burk double reciprocal plot, where all lines intersected on the horizontal axis, and the value of −1/Km (horizontal axis intercept) remained constant. In contrast, the value of Vmax decreased with increasing concentrations of the combination (Figure 7I). These findings confirm that TB binds to MUC2 through non‐covalent bonds, leading to non‐competitive and reversible inhibition of α‐glucosidase.

## 3 Discussion

Pu‐erh tea is increasingly gaining recognition for its potential to improve hypolipidemia and reduce cholesterol levels by modulating gut microbiota and bile acid metabolism [15, 17]. Some studies indicate that Pu‐erh tea reduces insulin resistance and glucose levels in OGTT [35, 36]. In our study, no significant improvements in OGTT glucose levels, insulin sensitivity, or GLP‐1 levels were observed in either IGT individuals or hyperglycemic model mice following Pu‐erh tea intervention, possibly due to the relatively short duration of Pu‐erh tea intervention. However, the significant improvements in the BTT and OMTT glucose levels suggested a correlation with α‐glucosidase inhibition.

Alpha‐glucosidase, located at the brush border of the small intestine, increases PBG levels by breaking down oligosaccharides and disaccharides into glucose molecules [37]. In contrast, AGIs are commonly used to manage hyperglycemia after consuming carbohydrate‐rich diets [38, 39]. Our study demonstrated that TB inhibited α‐glucosidase activity in a female‐dependent manner, which was associated with small intestinal MUC2 levels.

TB, a key bioactive component of Pu‐erh tea, is an oxidative polymerization product of theaflavins, thearubigins, and other related compounds, with a broad molecular weight distribution (3.5–100 kDa) [40]. Despite the recognition of TB's significant α‐glucosidase inhibition activity, the detailed mechanisms and the influence of gut environmental factors on its in vivo efficacy remain unclear. Furthermore, the inherent structural complexity of TB as a macromolecular polymer introduces potential challenges for complete experimental reproducibility. However, our standardized preparation protocol, developed in our prior work [17], allows for the consistent acquisition of a TB fraction that demonstrates stable and reproducible core bioactivity, which is foundational for the mechanistic investigations presented here. Our study assessed α‐glucosidase activity in the small intestinal mucus of mice before and after TB intervention, revealing a female‐dependent inhibitory effect of TB on α‐glucosidase activity, which was associated with small intestinal MUC2 levels.

MUC2, a secretory mucin protein produced by goblet cells, is a major macromolecular component of mucus that extends beyond maintaining the mucus barrier. Dysregulation of MUC2 production has been linked to various intestinal and metabolic diseases [41, 42]. Studies have shown a negative correlation between MUC2 expression in the intestine and conditions such as obesity, abnormal blood glucose levels, and hyperlipidemia [43, 44, 45, 46].

Estradiol plays a crucial role in regulating various biological functions, including promoting MUC2 expression in the gut to restore intestinal barrier function and potentially improve metabolic diseases [47, 48]. Our study reveals that estradiol is the most prominent sex‐differentiated metabolite in the gut, particularly in the regions of the small intestine that are rich in α‐glucosidase. We show that the intestinal mucosal MUC2 layer is significantly thicker in female mice than in male mice under physiological conditions. This finding was further validated through dose‐dependent upregulation of MUC2 expression in HT‐29 cells with increasing estradiol intervention. Transcriptional and protein levels of intestinal MUC2 were significantly reduced in ovariectomized mice, and the effect was rescued by estradiol supplementation. These results collectively demonstrate that intestinal MUC2 expression is regulated by estrogenic hormones. Additionally, because estradiol levels exhibit substantial variations across the female physiological period, as well as before and after menopause. As reported in the literature, there are 5–15 folds fluctuations in estradiol concentrations during the menstrual cycle, and estradiol levels may increase up to 100‐fold during pregnancy. However, estradiol concentrations are significantly reduced after postmenopause [49, 50, 51]. Therefore, we infer that TB‐mediated inhibition of postprandial hyperglycemia through E2‐MUC2 regulation may depend not only on sex but also on female physiological status and age‐related hormonal changes.

MUC2 contains tryptophan and tyrosine residues on its surface that can emit fluorescence [41]. While TB, a polymer of polyphenols, does not emit fluorescence, our study showed that TB gradually quenched the fluorescence of MUC2 with increasing concentrations in the MUC2 solution. This suggests an interaction between TB and MUC2, possibly facilitated by the phenolic hydroxyl and carboxyl groups in TB. Since TB is a structurally complex macromolecular polymer, theaflavins, as their potential precursors, are relevant within the metabolic pathway influencing gut mucosal interactions. Molecular docking simulations and fluorescence quenching assays in this study were also conducted to investigate the interactions between MUC2 and theaflavins. The results showed that MUC2 can interact with theaflavins (Figure S11A–D). Additionally, cell‐based co‐culture experiments showed that estradiol‐induced upregulation of MUC2 enhanced the formation of non‐covalent complexes between mucins and theaflavins, and the inhibitory activity of theaflavins on α‐glucosidase was further enhanced when MUC2 secretion was upregulated by estradiol compared to when estradiol was absent (Figure S11E,F). These findings demonstrate that hormonal modulation of mucin expression influences the gut microenvironment, which in turn affects the bioavailability and activity of theaflavins and their derivatives—representing the key mechanistic basis for sex differences in hypoglycemic efficacy. Collectively, our data support the hypothesis that sex hormones, such as estradiol, enhance mucin production, thereby facilitating interactions with TB and its potential precursors, theaflavins, which likely improve their stability and retention in the gastrointestinal tract. The complex formed by TB and its precursors with MUC2 may act as a “barrier” outside the active site of α‐glucosidase, potentially hindering the breakdown of oligosaccharides or disaccharides from the diet. We thus speculate that natural products rich in polyhydroxy groups, such as polyphenols or polysaccharides, may share similar inhibitory effects on intestinal α‐glucosidase in vivo, although further research is necessary to elucidate the exact mechanisms.

Sex‐specific differences in gastrointestinal physiology, including transporters, mucosal barriers, and metabolic enzyme expression, can impact the effectiveness of oral drugs and nutrients [52, 53]. Emerging evidence suggests that sex‐specific expression of efflux transporters can lead to varied therapeutic outcomes [54, 55]. Additionally, the mucus barrier influences the diffusion of drugs and nutrients in the gut [56]. Given MUC2's role as a primary component of the intestinal mucus layer, variations in MUC2 expression between sexes can result in differences in mucus layer thickness, ultimately affecting the effects of TB on α‐glucosidase inhibition in female and male mice.

There are some limitations in our study. While our study sheds light on the sex‐specific molecular mechanism underlying the glucose‐lowering effects of TB from Pu‐erh tea, further research with larger cohorts is needed to validate these findings. Understanding the comprehensive interaction between MUC2 and TB and exploring the role of MUC2 in enhancing TB's inhibitory effects on α‐glucosidase are critical areas for future investigation. Overall, considering sex‐specific factors in pharmacotherapy is essential for optimizing treatment outcomes.

In summary, our study first revealed the sex‐specific molecular mechanism behind the glucose‐lowering effects of TB from Pu‐erh tea. We found that TB, in conjunction with MUC2 upregulated by estradiol in the small intestine, enhanced the inhibition of α‐glucosidase, particularly reducing PBG levels in females. These findings not only provide an alternative intervention using a popular beverage to lower PBG in patients with IGT or even T2DM but also highlight the importance of considering sex factors in pharmacotherapy.

## 4 Experimental Section

### 4.1 Extraction of Instant Pu‐erh Tea

Instant Pu‐erh tea is produced by a specific, standardized manufacturer's protocol [17]. Briefly, the ripe Pu‐erh tea is extracted with water using a multi‐stage countercurrent extraction (MCEE) process and then spray‐dried to obtain the instant Pu‐erh tea powder for use.

### 4.2 Theabrowin Source

Theabrowin (2022‐1‐10) was obtained from Yunnan Tangren Biotechnology Co., Ltd (produced according to the same standardized procedure as ours [17]).

### 4.3 Human Study

The inclusion criteria were (1) male and female individuals aged between 18 and 60 years; (2) normal glucose tolerance (NGT): FPG < 6.1 mmol L^−1^ and 2hPG < 7.8 mmol L^−1^; impaired glucose tolerance (IGT): FPG < 6.1 mmol L^−1^, 7.8 mmol L^−1^ ≤ 2hPG < 11.1 mmol L^−1^; (3) body mass index (BMI) ranging from 19.0 to 35.0 kg/m^2^, with male body weight ≥ 50 kg and female body weight ≥ 45 kg; (4) no history of taking glucose‐lowering agents; (5) no habit of long‐term drinking tea or coffee.

The exclusion criteria were (1) acute illness or current evidence of acute or chronic inflammatory or infectious diseases; (2) individuals with a history of allergy to tea or its extracts; (3) Female subjects who are pregnant or lactating; (4) Current systemic corticosteroid therapy or medications affecting glucose metabolism.

This study was approved by the ethics committee of Shanghai Sixth People's Hospital Affiliated to Shanghai Jiao Tong University School of Medicine and adhered to the tenets of the Declaration of Helsinki (Approval No. 2023‐KY‐066(K) and 2024‐KY‐082(K)). All subjects provided a full explanation of the study procedures, and written informed consent was obtained from each participant. The study was registered at the Chinese Clinical Trial Register (ChiCTR): ChiCTR2400091051.

### 4.4 Mouse Models

All animal studies were approved by the Institutional Animal Care and Use Committee of the Center for Laboratory Animals, Shanghai Sixth People's Hospital Affiliated to Shanghai Jiao Tong University School of Medicine, Shanghai, China (ethics approval number: No. 2024‐0088). All the mice were maintained under a specific‐pathogen‐free (SPF) environment in controlled conditions with free access to food and water (12 h light/dark cycle at 20°C–22°C, humidity 45 ± 5%). Mice were acclimated in an animal facility with a chow diet ad libitum for one week. The chow diet (TP23522, Trophic Animal Feed High‐tech Co., Ltd, China) and HFD (D12492, Research Diets, Inc) were used in animal experiments. During the experiments, the body weights and food consumption were measured once a week.

### 4.5 Human Study

Study 1: NGT (n = 20) and IGT (n = 20) individuals were recruited and underwent an adaptation period. All individuals received a uniform background diet. The diet provided 25 kcal kg^−1^ ideal body weight daily [57, 58]. They were advised against extra beverages. Based on the recommended dosage of instant Pu‐erh tea (4–6 sachets daily) and the dosages used in our preliminary studies, they orally consumed instant Pu‐erh tea infusion (scented Deepure, Tasly holding group, Tianjin, China) dissolved in 300 mL of boiling water twice daily at dosages of 50 mg/kg/day for 4 weeks. At the beginning and the end of interventions, individuals underwent a bread tolerance test (BTT), an oral maltose tolerance test (OMTT), and an oral glucose tolerance test (OGTT). Other indicators, including fasting blood glucose, fasting insulin, and the serum active GLP‐1, were assessed.

Study 2: NGT (n = 34) and IGT (n = 40) individuals were recruited and underwent an adaptation period. All individuals underwent two OMTTs. The first OMTT was conducted while fasting. One week after the first OMTT, the second OMTT was conducted following a single oral administration of TB at a dose of 25 mg/kg, dissolved in 300 mL of boiling water. During this study, all individuals received the same dietary protocol as implemented in Study 1.

Serum and fecal samples were collected and stored at −80°C until analysis.

### 4.6 Metabolic Assay

To evaluate glucose metabolism in the NGT and IGT individuals, serial blood samples were collected in both fasting and postprandial states for laboratory tests following 1) OBTT: oral administration bread (100 g per person); 2) OMTT: oral administration of maltose (75 g per person); 3) OGTT: oral administration of glucose (75 g per person).

### 4.7 Measurement of Serum Biochemical Indicators

Plasma glucose levels were quantified by the conventional hexokinase method using an automatic biochemical analyzer Cobas 6000 (Roche Diagnostics International, Switzerland) according to the reference [59]. In detail, plasma samples were collected in tubes with EDTA‐containing, centrifuged at 3000 × g for 10 min at 4°C, and then analyzed within 2 h to minimize glycolysis. Glucose‐derived products, NADPH or NADH generated in serum, are measured by recording the absorbance at 340 nm, which is proportional to the glucose concentration in the sample.

Serum insulin levels were assayed by a common radioimmunoassay (Linco Research, St. Charles, MO) [60]. Insulin in serum samples competes with a fixed amount of iodine‐125 (^125^I)‐labeled insulin for binding sites on anti‐insulin polyclonal antibodies. After incubation at 4°C for 24 h, bound and free fractions were separated by polyethylene glycol (PEG) precipitation. Radioactivity of the antibody‐bound fraction was measured using a gamma counter, and insulin concentrations were calculated against a standard curve (0–200 µU/mL) generated with human insulin calibrators. The results were adjusted for nonspecific binding using blank tubes containing no antibody.

$$\mathrm{HOMA}-\mathrm{IR}=\mathrm{FINS}\;\left(\mathrm{mU}\;L^{-1}\right)\times \mathrm{FPG}\;\left(\mathrm{mmol}\;L^{-1}\right)/\;22.5$$

Serum active GLP‐1 levels were determined using an active GLP‐1 assay kit (ml022729A, Mlbio).

### 4.8 Animal Experiments

6 weeks old, and sex matched C57BL/6J mice were used in all studies.

Animal experiment 1: Pu‐erh Tea intervention in HFD mice

Mice were randomly divided into two groups with 8 mice of each sex in each group: (1) HFD group: mice were fed with HFD; (2) HFD + Pu‐erh tea group: mice were fed HFD with 3 mg/mL instant Pu‐erh tea infusion at a dose of 450 mg/kg/day. Mice were euthanized after 8 weeks of intervention to collect serum, intestinal tissues, contents, and small intestinal mucosa.

Animal experiment 2: Pu‐erh Tea intervention in HFD+STZ mice

After mice were raised with HFD for 8 weeks, all the mice were fasted for 5 h and then were intraperitoneally injected with a single dosage of 75 mg/kg streptozotocin (STZ; V900890, Sigma‐Aldrich). Mice with glucose levels above 11.1 mmol/L 72 h after STZ injection were used in the study. The HFD+STZ mice were randomly divided into two groups with 8 mice of each sex in each group: (1) Control group: mice were fed with HFD; (2) Pu‐erh tea group: mice were fed with HFD with 3 mg/mL instant Pu‐erh tea infusion at a dose of 450 mg/kg/day. Mice were euthanized after 4 weeks of intervention to collect serum, intestinal tissues, contents, and small intestinal mucosa.

Animal experiment 3: TB intervention in normal mice

Mice were randomly divided into five groups with 6 mice of each sex in each group: (1) Control group: mice were administered intragastrically with phosphate‐buffered saline (PBS); (2) Acarbose group: mice were administered with Acarbose by gavage at a dose of 5 mg/kg as positive control; (3) TB‐Low dose group: mice were orally administered with TB at a dose of 112.5 mg/kg; (4) TB‐Middle dose group: mice were orally administered with TB at a dose of 225 mg/kg; (5) TB‐High dose group: mice were orally administered with TB at a dose of 450 mg/kg. After an overnight fast, mouse blood glucose levels were measured following orally administered maltose at a dose of 2 g/kg, along with either PBS or acarbose, or varying doses of TB (low, middle, or high). After a 1‐week interval, each group of mice was fasted overnight and then orally administered either PBS or acarbose or varying doses of TB (low, middle, or high) or PBS. 45 min later, all mice were euthanized to collect serum, intestinal tissues, contents, and small intestinal mucosa.

Animal experiment 4: TB intervention in HFD+STZ mice

The HFD+STZ diabetic model mice are established as previously described. The HFD+STZ mice were randomly divided into three groups, with 6 mice of each sex in each group: (1) Control group: mice were administered with PBS intragastrically; (2) Acarbose group: mice were administered with Acarbose by gavage at a dose of 5 mg/kg; (3) TB group: mice were administered with TB by gavage at a dose of 225 mg/kg. After an overnight fast, mouse blood glucose levels were measured following orally administered maltose at a dose of 1 g/kg, along with either PBS, Acarbose, or TB. After a 1‐week interval, each group of mice was fasted overnight and then orally administered either PBS, acarbose, or TB. 45 min later, all mice were euthanized to collect serum, intestinal tissues, contents, and small intestinal mucosa.

Animal experiment 5: TB with estradiol (E2) intervention in normal male mice

Mice were randomly divided into five groups with 6 in each group: (1) Male control group: male mice were administered intragastrically with PBS; (2) Male TB group: male mice were administered with TB by gavage at a dose of 225 mg/kg; (3) Male E2 group: male mice were administered with E2 by gavage at a dose of 0.6 ng per mouse; (4) Male E2+TB group: male mice were administered with E2 at a dose of 0.6 ng per mouse, and TB at a dose of 225 mg/kg 10 min later; (5) Female TB group: female mice were administered with TB by gavage at a dose of 225 mg/kg; After the mice fasted an overnight, mouse blood glucose levels of group (1), (2) and (5) were measured following orally administered at a dose of maltose (2 g/kg) along with TB or PBS. For the rest two groups, blood glucose levels were measured after oral gavage with E2 at a dose of 0.6 ng per mouse, followed by oral administration of maltose (2 g/kg) along with either PBS or TB (225 mg/kg) 10 min later. After a 1‐week interval, mice were fasted overnight and then orally administered similarly as above without maltose. The mice were euthanized for collecting serum, intestinal tissues, intestinal contents 45 min later, and small intestinal mucosa.

Animal experiments 6: TB with E2 intervention in male HFD+STZ mice

The HFD+STZ diabetic model mice are established as previously described. The male HFD+STZ mice were randomly divided into five groups with 6 in each group: (1) Control group: mice were administered intragastrically with PBS; (2) Acarbose group: mice were administered with Acarbose by gavage at a dose of 5 mg/kg; (3) TB group: mice were administered with TB by gavage at a dose of 225 mg/kg; (4) E2 group: mice were administered with E2 by gavage at a dose of 0.6 ng per mouse; (5) E2+TB group: mice were administered with E2 at a dose of 0.6 ng per mouse, followed with TB by gavage at a dose of 225 mg/kg 10 min later. After the mice fasted overnight, the mouse blood glucose levels of groups (1), (2), and (5) were measured following oral administration at a dose of maltose (1 g/kg) along with TB or PBS. For the rest two groups, blood glucose levels were measured after oral gavage with E2 at a dose of 0.6 ng per mouse, followed by oral administration of maltose (1 g/kg) along with either PBS or TB (225 mg/kg) 10 min later. After a 1‐week interval, mice were fasted overnight and then orally administered similarly as above without maltose. The mice were euthanized for collecting serum, intestinal tissues, intestinal contents 45 min later, and small intestinal mucosa.

Animal experiment 7: TB Intervention in ovariectomized female mice

Mice underwent ovariectomy (OVX) at 4 weeks of age. All mice were anesthetized via intraperitoneal injection of xylazine (100 mg/kg) and ketamine (12.5 mg/kg). For the OVX group, mice received bilateral dorsolateral incisions through the skin, subcutaneous fat, and muscle to expose the peritoneal cavity. The ovaries were separated from the visceral fat, ligated, and removed. For the sham group, mice received only bilateral dorsolateral skin incisions, which were then closed in the same manner as those in the OVX group. The incisions were closed using dissolvable Vicryl sutures. Sham groups were randomly divided into two groups with 6 in each group. (1) Sham control group: mice were administered intragastrically with PBS; (2) Sham TB group: mice were administered with TB by gavage at a dose of 225 mg/kg. OVX groups were randomly divided into four groups with 6 in each group. (1) OVX control group: mice were administered intragastrically with PBS; (2) OVX TB group: mice were administered with TB by gavage at a dose of 225 mg/kg; (3) OVX E2 group: male mice were administered with E2 by gavage at a dose of 0.6 ng per mouse; (4) OVX E2+TB group: male mice were administered with E2 at a dose of 0.6 ng per mouse, followed with TB by gavage at a dose of 225 mg/kg 10 min later. After an overnight fast, maltose (2 g/kg) was orally administered to the mice, along with either TB (225 mg/kg) or PBS. For the other two groups, E2 was administered by gavage at a dose of 0.6 ng per mouse, followed 10 min later by oral administration of maltose (2 g/kg) along with either PBS or TB (225 mg/kg). Blood glucose levels were measured using a glucometer. After a 1‐week interval, the mice were fasted overnight again and then orally administered according to the same method as previously described for each group. These mice were euthanized 45 min later for the collection of serum, intestinal tissues, contents, and small intestinal mucosa at the end of the experiment.

### 4.9 Metabolic Assay

For the OMTT, mice were fasted overnight from 8:00 p.m. to 8:00 a.m. Glucose levels in tail vein blood samples were measured using a glucose glucometer (OneTouch Ultra, Lifescan, Johnson & Johnson, Milpitas, CA) at 0, 15, 30, 60, and 120 min after oral administration of maltose. The dosage was 1 g/kg for HFD mice and HFD+STZ mice, and 2 g/kg for normal mice.

For the OGTT, mice were fasted overnight from 8:00 p.m. to 8:00 a.m. Glucose levels in tail vein blood samples were measured using a glucometer (OneTouch Ultra, LifeScan, Johnson & Johnson, Milpitas, CA) at 0, 15, 30, 60, and 120 min after oral administration of glucose. The dosage was 1 g/kg for HFD mice and HFD+STZ mice.

For the ITT, insulin (2 U/kg) was administered via intraperitoneal injection to HFD mice and HFD+STZ mice after a 4‐h fast (from 6:00 a.m. to 10:00 a.m.). Glucose levels in tail vein blood samples were measured at 0, 15, 30, 60, and 90 min following insulin administration

### 4.10 Measurement of GLP‐1 and Insulin in Mice

Mice were fasted overnight, 1 h before the administration of a liquid diet (Ensure Plus, 10 mL/kg; Abbott), and then gavaged with the DPP‐4 inhibitor sitagliptin (MK0431, MCE; 3 mg/kg). Fifteen minutes after the food gavage, retro‐orbital blood samples (approximately 100 µL) were collected. Active GLP‐1 levels were measured using an active GLP‐1 assay kit (m1201801, Mlbio). At the time of sacrifice, after an overnight fast, retro‐orbital blood samples for serum analysis were collected. All samples were stored at −80°C until analysis. Insulin levels were measured using a mouse insulin ELISA kit (mi001983, Mlbio).

### 4.11 Assay of Activity of Small Intestinal Mucosa on α‐Glucosidase

After removing impurities from the fresh mouse small intestine contents, the intestines were gently rinsed with PBS (pH 6.8) at 4°C. Under ice‐cold conditions, the intestines were cut open, and the mucosa and surface mucus from different sections of the small intestine (duodenum, jejunum, ileum) were scraped onto clean slides [61]. An α‐glucosidase activity assay kit (BC2550, Solarbio) was used to measure active α‐glucosidase. The inhibition percentage was calculated using the following formula:Inhibition rate (%)  =  [(C − I) /C]  ×  100, where C represents the α‐glucosidase activity of the control group, and I represents the α‐glucosidase activity of the intervention group.

### 4.12 Measurement of the Components of Pu‐erh Tea

Tea components, including tea polyphenols and tea pigments, were quantified in the small intestinal contents of mice using ultra‐performance liquid chromatography tandem quadrupole‐time‐of‐flight mass spectrometry (UPLC/QTOFMS), as described in our previous study [17].

### 4.13 Assay of α‐Glucosidase Activity in Vitro

The α‐glucosidase inhibitory effect was determined using a previously described method with minor modifications [62]. The reaction mixture consisted of 50 µL of phosphate buffer (0.1 M, pH 6.9), 20 µL of test sample solution, and 20 µL of 12.5 mM pNPG. The mixture was incubated at 37°C for 10 min, followed by the addition of 10 µL of 2.5 U/mL enzyme solution as a substrate. After an additional 20‐min incubation at 37°C, the reaction was stopped by adding 100 µL of 0.2 M Na2CO3 solution. Absorbance was then measured at 405 nm using a microplate reader (SpectraMax i3x, Molecular Devices, China). The inhibition rate of the reagents on α‐glucosidase was calculated using the following equation:

$$\mathrm{Inhibition}\;\mathrm{rate}\;\left(\%\right)=\left[\left(A_{1}-A_{2}\right)-\left(A_{3}-A_{4}\right)\right]/\left(A_{1}-A_{2}\right)$$

where A_1_ is the absorbance without reagents and with α‐glucosidase, A_2_ is the absorbance without reagents and without α‐glucosidase, A_3_ is the absorbance with reagents and with α‐glucosidase, and A_4_ is the absorbance with reagents and without α‐glucosidase.

### 4.14 Determination of Kinetic Characterization of α‐Glucosidase

To determine the inhibition kinetic characteristics of α‐glucosidase, absorbance values of the reaction solution were measured by fixing the concentrations of the sample and substrate while varying the enzyme concentration (1, 2, 3, 4, 5 U/mL) [63]. Deionized water was used as the blank in place of the sample solution to ascertain whether the inhibition by the sample on α‐glucosidase was reversible or irreversible. Furthermore, absorbance values were measured under varying substrate concentrations (1, 2.5, 5, 7.5, 12.5 mmol/L) while maintaining constant concentrations of the sample and enzyme. Deionized water served as the blank solution. A Lineweaver‐Burk plot was used to determine competitive or non‐competitive reversible inhibition exerted by the sample on the enzyme reaction [64, 65].

### 4.15 Determination of Fluorescence Intensity

Intrinsic fluorescence spectra were obtained using a microplate reader (SpectraMax i3x, Molecular Devices, China) following a previously described method with minor modifications [66]. Emission wavelengths were recorded within the range of 300–450 nm, with the excitation wavelength set at 280 nm for temperatures of 298 K, 304 K, and 310 K. Both the excitation and emission slits were set to 2.5 nm. The concentration of α‐glucosidase was maintained at 80 µg/mL. The solution was prepared with phosphate buffer (0.1 M, pH 6.8) mixed with varying concentrations of TB solution, and equal volumes of MUC2 were added and measured. The Stern‐Volmer equation was applied to evaluate the quenching mechanism:

$$F_{0}/F\;=1+K_{q}\tau_{0}\left[\mathrm{TB}\right]=1+K_{\mathrm{sv}}\left[\mathrm{TB}\right]$$

F_0_ and F represent the fluorescence intensities of MUC2 in the absence and presence of TB, respectively, denotes the concentration of TB (quencher), K_SV_ is the Stern‐Volmer quenching constant, Kq (K_SV_/τ_0_) represents the bimolecular quenching rate constant, and τ_0_ (10^−8^ s) represents the average lifetime of the fluorophore in the absence of a quencher.

### 4.16 Sex Hormones Measurement

Six sex hormones, including estrogens (estrone, estradiol, estriol) and androgens (androstenedione, testosterone, dihydrotestosterone), were quantified in human fecal and mouse small intestinal contents according to the reference [67, 68]. A 20 mg sample was weighed, followed by the addition of 20 µL of water and 600 µL of Methyl tert‐Butyl Ether (MTBE) containing internal standards (estrone‐d4, testosterone‐d4). The mixture was ground for extraction and then vortexed thoroughly. After centrifugation at 12 700 rpm for 10 min, 400 µL of the MTBE layer was transferred to a new centrifuge tube and dried for 2 h. Subsequently, 50 µL of methanol was added for reconstitution, followed by the addition of 50 µL of water. Each mixture was centrifuged at 12 700 rpm for 10 min, and the supernatant was used for UPLC/TQ‐MS analysis. Raw data from UPLC‐MS were analyzed and quantified using TargetLynx version 4.1 applications manager (Waters Corp., Milford, MA).

### 4.17 Metabolites Measurement

Targeted metabolomics analysis of small intestinal contents samples was performed using the Q300 Metabolite Assay Kit (Human Metabolomics Institute, Inc., Shenzhen, Guangdong, China) according to the recommended SOP, following our previously published method [69, 70]. The fold change > 2 with p values < 0.05 was considered a cutoff for differential metabolites.

### 4.18 Proteomics Analysis

The jejunal mucus from mice was mixed with pre‐cooled PBS and lysed with SDT buffer to extract proteins. Protein concentrations were determined using the BCA method, and samples were trypsin‐digested via FASP. The resulting peptides were desalted, lyophilized, and solubilized in 0.1% formic acid before quantification at OD280 [71].

For separation, samples were loaded onto a NanoElute HPLC system with a C18 column at a flow rate of 300 nL/min. Mass spectrometry was conducted using a time TOF Pro mass spectrometer in positive ion mode with a source voltage of 1.5 kV. MS and MS/MS data were collected in the range of 100–1700 m/z using PASEF mode, with specific ion mobility settings and a ratio of 10 secondary spectra per primary spectrum.

Raw mass spectrometry data were analyzed using MaxQuant software (v1.6.14) [72], with a cutoff for differential proteins set at fold changes > 1.2 and p values < 0.05.

### 4.19 Alcian Blue and Periodic Acid‐Schiff Stain (AB‐PAS)

Paraffin‐embedded jejunal sections were deparaffinized in xylene and rehydrated through gradient alcohols. After staining goblet cells with Alcian blue, the sections were rinsed with pure water, then oxidized with periodic acid, and rinsed again with pure water. The sections were then dehydrated and sealed [73]. Finally, the digital sections were prepared using a slice scanner (Pannoramic MIDI).

### 4.20 Immunofluorescence Staining

Jejunal tissue was fixed in a 4% paraformaldehyde solution and then embedded in paraffin according to standard procedures [74]. The samples embedded in paraffin were sectioned and further analysis. The slides were deparaffinized in xylene, rehydrated through gradient alcohols, and stained with anti‐MUC2 antibody (1:1000, 27675‐1‐AP, RRID: AB_2880943, Proteintech). Subsequently, the slides were incubated with Alexa Fluor 594‐conjugated goat anti‐rabbit IgG (1:1000, ab150080; RRID: AB_2650602, Abcam), and immunostaining signals and DAPI‐stained nuclei were visualized.

Images were acquired using a confocal microscope camera (Zeiss LSM 900, Zeiss, Germany) and further analyzed using ImageJ or ZEN software.

### 4.21 Real‐Time Quantitative PCR

Total RNA was isolated from cells or tissue samples using a total RNA isolation kit using FastPure Cell/Tissue Total RNA Isolation Kit V2 (Cat: RC112‐01, Vazyme Biotech Co., Ltd., China) according to the manufacturer's instructions. The concentration of total RNA was measured using a NanoDrop 2000C spectrophotometer (Thermo Fisher Scientific, Waltham, MA, USA). Purified, 500 ng per sample RNA was reversed by PrimerScript RT Reagent Kit with gDNA eraser (RR047Q, Takara, Kusatsu, Japan). The primers for qPCR analysis were designed and synthesized in Sangon Biotech (Shanghai, China) (Table S3). The quantitative real‐time PCR reaction was conducted by a Taq Pro Universal SYBR qPCR Master Mix (Cat: Q712‐02, Vazyme Biotech Co., Ltd., China), and the reaction was finished by using an ABI Q7 PCR System (Applied Biosystems Instruments, Thermo Fisher Scientific, USA). The values of the target genes were normalized to GAPDH, and the relative expression levels were shown as fold changes relative to the control group.

### 4.22 Western Blot Analysis

Mouse small intestine, intestinal fluid, cell, and cell supernatant samples were lysed with RIPA buffer (Beyotime Technology, Shanghai, China) containing 1 mM phenylmethylsulfonyl fluoride (PMSF) (Beyotime Technology, Shanghai, China) on ice, then centrifuged at 14 000 g for 5 min. The supernatants were collected, and the concentration of total protein was quantified using a BCA Protein Assay Kit (Thermo, California, USA). A 5 µg/µL protein extract was added to the loading buffer (Beyotime Technology, Shanghai, China) and denatured by boiling at 100°C for 10 min. Equal amounts of protein were electrophoresed on 10% SDS‐PAGE gels and transferred to a PVDF membrane. The membrane was blocked with 5% non‐fat milk and then incubated with antibodies against MUC2 (1:1000, 27675‐1‐AP, RRID: AB_2880943, Proteintech), β‐actin (1:1000, 4970, RRID: AB_2223172, Cell Signaling Technology) at 4°C overnight. The membranes were washed three times with tris‐buffered saline +Tween 20 (TBST) buffer and again following a 2‐h incubation with HRP‐conjugated secondary anti‐rabbit (1:1000, 7074, RRID: AB_2099233, Cell Signaling Technology). The blots were visualized using an ECL kit (Bio‐Rad, CA).

### 4.23 Cell Culture and Cell Co‐Culture

The Caco‐2 (BTCC‐1033, BTCC, China) and HT‐29 (BTCC‐1075, BTCC, China) cells were cultured in Dulbecco's modified Eagle's medium (DMEM, Gibico). Caco‐2 cells producing α‐glucosidase were used to evaluate α‐glucosidase activity. HT‐29 cells producing MUC2 were used to assess MUC2 expression. 10% fetal bovine serum (FBS) (Gibico, qualified, Australia origin) and 1% Penicillin / Streptomycin mix were supplemented in the basic medium. All cells were incubated at 37°C in an atmosphere containing 5% CO_2_ in air and were refreshed every 2 days [75].

Caco‐2 and HT‐29 cells were counted using an automated cell counter (Cellometer Auto T4, Nexcelom Bioscience), mixed in a 3:1 ratio (Caco‐2 to HT‐29), and then seeded into 24‐well plates at a final density of 0.8 × 10^4^ cells/cm^2^. The cells were cultured under the same conditions as previously described and allowed to grow for 21 days, with the medium being refreshed every 2 days.

### 4.24 *MUC2* Knocks Out

We used Dharmacon Edit‐R All‐in‐one Lentiviral and Edit‐RCRISPRa Lentiviral systems to create gene knock‐out (KO) cell lines. The sgRNA sequences are as follows: *MUC2*‐1 forward, 5’‐CACCGAACTTCGCCTCCGACTGCCG‐3’ and reverse, AAACCGGCAGTCGGAGGCGAAGTTC‐3; *MUC2*‐2 forward, 5’‐CACCGGAAGATCAACCAGCCCGATG‐3’ and AAACCATCGGGCTGGTTGATCTTCC‐3’; Retroviral particles were produced by co‐transfection of 293T cells with PMD, and PsPAX, packaging plasmids using polyethyleneimine (PEl) as a transfection reagent (3:1 mass ratio of PEI: DNA) [76]. Stable cell lines were created by infecting the transfected cells according to the manufacturer's instructions. The knockdown of target proteins was verified by Western blot.

### 4.25 Assay of α‐Glucosidase Activity in Cells

Caco‐2 was cultured for 21 days, after which the medium was replaced with 300 µL of phenol red and FBS‐free DMEM medium and cultured for an additional 24 h. E2 and different concentrations of Acarbose and TB were added to the culture medium in the intervention groups, while the control group received the corresponding amount of PBS.

Caco‐2/HT‐29 or Caco‐2/ (MUC2‐/‐) HT‐29 cells were cultured for 21 days and then divided into two groups. (1) MUC2 group: The medium was replaced with 300 µL of phenol red and FBS‐free DMEM medium and cultured for an additional 24 h. E2 and varying concentrations of Acarbose and TB were added to the culture medium. In the E2+TB groups, E2 was added 5 min before the other reagents and incubated at 37°C, while the control group received an equivalent amount of PBS. (2) Without MUC2 group: Before adding the intervention reagents, the medium was replaced with 300 µL of PBS. The other processes were the same as those for the MUC2 group.

All cells were incubated at 37°C for 20 min. Following incubation, the cells were collected under ice‐cold conditions and counted. The α‐glucosidase activity in the cells was measured using an α‐glucosidase activity assay kit (m1201801, Solarbio). The inhibition percentage was calculated using the following equation:

$$\mathrm{Inhibition}\;\mathrm{rate}\;\left(\%\right)=\left[\left(C-I\right)/C\right]\times 100$$

where C represents the α‐glucosidase activity of the control group, and I represents the α‐glucosidase activity of the intervention group.

### 4.26 Statistical Analysis

Results were presented as mean ± SEM. Data analysis and graphical presentation in this study were generated by GraphPad Prism 9.0 (GraphPad Software, La Jolla, CA, USA). All tests applied were 2‐tailed. The sample distribution was determined using a Kolmogorov‐Smirnov normality test. For statistical comparisons, one‐way ANOVA tests were used to compare normally distributed variables. Non‐normal distributed data were compared by the Kruskal‐Wallis test with the Benjamini‐Hochberg false discovery rate test performed by SPSS 26.0 (IBM SPSS, Chicago, IL, USA). Spearman or Pearson's correlation analysis was visualized via R studio (RStudio, Boston, MA, USA). p < 0.05 was considered statistically significant.

## Author Contributions

W.J. conceptualized the study. W.J., A.Z., and G.X. designed the research. A.Z. and G.X. organized all the in vivo studies and critical discussions of the results. Y.L. and A.Z. performed the experiments and the overall analysis. Y.L., J.K., and D.Z. performed the assay of α‐glucosidase activity and immunofluorescence staining. Y.L., J.K., J.W., X.Z., Z.R., F.H., and M.Z. contributed to the animal experiments. D.Z., K.G., and C.Q. performed metabolite measurements. Y.L., D.Z., J.W., K.G., X.Z., Z.R., F.H., M.Z., L.L., X.M., and Y.X. contributed to data analysis. Y.L., J.K., W.J., Y.S., H.Z., K.D., X.X., Y.T., J.Z., and F.G. were responsible for the human sample collection and explanation. W.J. and A.Z. provided funding. Y.L. and A.Z. drafted the manuscript and all the figures. W.J., A.Z., and G.X. critically revised the paper. All authors have read and agreed to the submitted version of the manuscript.

## Conflicts of Interest

The authors declare no conflicts of interest.

## Supporting information




**Supporting File**: advs75314‐sup‐0001‐SuppMat.docx.

**Supporting File**: advs75314‐sup‐0002‐RawData.zip.

## Data Availability

The data that support the findings of this study are available from the corresponding author upon reasonable request.
