# Supplementary material for: Sex‐Specific Regulation of Glycemic Homeostasis by Theabrownin from Pu‐erh Tea
Source: Adv Sci (Weinh). 2026 Apr 16;13(38):e19337. doi: 10.1002/advs.202519337 (PMC13335617; doi:10.1002/advs.202519337)
Supplement: Supplementary file 1 — Supporting File: advs75314‐sup‐0001‐SuppMat.docx. [file ADVS-13-e19337-s002.docx]

**Supplementary**

**Table S1 Clinical parameters of the NGT and IGT individuals before 4-week Pu-erh tea intervention**

|  | **NGT (n = 20)** | **Male (n = 10)** | **Female**  **(n = 10)** | **P Value (**Male vs Female） | **IGT  (n = 20)** | **Male (n = 10)** | **Female  (n = 10)** | **P Value (Male vs Female**） |
| --- | --- | --- | --- | --- | --- | --- | --- | --- |
| Age (year) | 28 ± 4 | 28 ± 3 | 28 ± 6 | 0.9598 | 37 ± 7 | 37 ± 8 | 37 ± 7 | 0.9771 |
| BMI (kg/m^2^) | 21.04 ± 2.24 | 21.73 ± 2.80 | 20.34 ± 1.31 | 0.1726 | 27.87 ± 3.89 | 27.75 ± 4.51 | 28.00 ± 3.40 | 0.8870 |
| FPG (mmol/L) | 5.03 ± 0.36 | 4.91 ± 0.39 | 5.15 ± 0.30 | 0.1417 | 5.12 ± 0.53 | 517 ± 0.51 | 5.07 ± 0.58 | 0.6730 |
| 2hPG (mmol/L) | 6.18 ± 0.43 | 6.09 ± 0.54 | 6.27 ± 0.28 | 0.3449 | 8.95 ± 0.83 | 9.14 ± 0.94 | 8.77 ± 0.70 | 0.3311 |
| TC (mmol/L) | 4.52 ± 0.81 | 4.50 ± 0.97 | 4.54 ± 0.66 | 0.9193 | 4.89 ± 0.78 | 4.78 ± 0.69 | 4.99 ± 0.88 | 0.5646 |
| TG (mmol/L) | 0.99 ± 0.43 | 1.07 ± 0.50 | 0.91 ± 0.36 | 0.4293 | 1.58 ± 0.71 | 1.71 ± 0.66 | 1.46 ± 0.77 | 0.4474 |
| HDL-C (mmol/L) | 1.49 ± 0.18 | 1.46 ± 0.25 | 1.52 ± 0.10 | 0.5196 | 1.23 ± 0.24 | 1.14 ± 0.20 | 1.33 ± 0.26 | 0.0948 |
| LDL-C (mmol/L) | 2.56 ± 0.72 | 2.65 ± 0.89 | 2.47 ± 0.54 | 0.5945 | 3.05 ± 0.57 | 3.01 ± 0.45 | 3.10 ± 0.68 | 0.7471 |
| ALT (U/L) | 20.45 ± 6.42 | 21.00 ± 11.95 | 19.90 ± 7.00 | 0.7125 | 34.60 ± 14.31 | 36.80 ± 17.11 | 32.40 ± 11.33 | 0.5065 |
| AST (U/L) | 25.33 ± 7.29 | 22.1 ± 6.21 | 21.1 ± 6.98 | 0.7389 | 29.30 ± 7.76 | 28.60 ± 6.79 | 30.00 ± 8.94 | 0.6980 |
| HbA1c (%) | 5.26 ± 0.19 | 5.32 ± 0.20 | 5.20 ± 0.19 | 0.1831 | 5.55 ± 0.32 | 5.60 ± 0.36 | 5.49 ± 0.29 | 0.4598 |

BMI, body mass index; FPG, fasting plasma glucose; 2hPG, 2-h plasma glucose during OGTT; TC, Total Cholesterol; TG, Triglycerides; HDL-C, high-density lipoprotein cholesterol; LDL-C, low-density lipoprotein cholesterol; ALT, alanine transaminase; AST, aspartate transaminase; Data are shown as mean ± SEM.

**Table S2 Clinical parameters of the NGT and IGT individuals prior to a single TB intervention**

|  | **NGT (n = 34)** | **Male (n = 17)** | **Female**  **(n = 17)** | **P Value (**Male vs Female） | **IGT  (n = 40)** | **Male (n = 20)** | **Female  (n = 20)** | **P Value (Male vs Female**） |
| --- | --- | --- | --- | --- | --- | --- | --- | --- |
| Age (year) | 34 ± 10 | 35 ± 8 | 34 ± 12 | 0.8714 | 39 ± 10 | 39 ± 9 | 37 ± 10 | 0.6025 |
| BMI (kg/m^2^) | 21.67 ± 2.08 | 22.25 ± 1.87 | 21.09 ± 2.11 | 0.1017 | 25.28 ± 2.69 | 25.17 ± 2.17 | 25.40 ± 3.19 | 0.7916 |
| FBG (mmol/L) | 4.85 ± 0.41 | 4.86 ± 0.44 | 4.85 ± 0.36 | 0.9704 | 5.32 ± 0.50 | 5.40 ± 0.49 | 5.23 ± 0.52 | 0.3515 |
| 2hPBG (mmol/L) | 6.14 ± 0.70 | 6.06 ± 0.74 | 6.21 ± 0.65 | 0.5493 | 9.29 ± 0.85 | 9.51 ± 0.89 | 9.08 ± 0.78 | 0.1167 |
| TC (mmol/L) | 4.30 ± 0.45 | 4.30 ± 0.49 | 4.29 ± 0.34 | 0.9645 | 4.91 ± 1.12 | 4.93 ± 1.13 | 4.90 ± 1.14 | 0.9355 |
| TG (mmol/L) | 1.04 ± 0.40 | 1.09 ± 0.46 | 0.97 ± 0.28 | 0.4313 | 1.75 ± 0.91 | 1.93 ± 1.10 | 1.55 ± 0.63 | 0.1825 |
| HDL-C (mmol/L) | 1.51 ± 0.22 | 1.48 ± 0.28 | 1.54 ± 0.14 | 0.4230 | 1.32 ± 0.26 | 1.25 ± 0.25 | 1.37 ± 0.18 | 0.1048 |
| LDL-C (mmol/L) | 2.69 ± 0.42 | 2.78 ± 0.47 | 2.60 ± 0.33 | 0.2334 | 3.17 ± 0.73 | 3.28 ± 0.73 | 3.06 ± 0.74 | 0.3465 |
| ALT (U/L) | 29.15 ± 11.16 | 30.47 ± 13.56 | 27.82 ± 7.58 | 0.4976 | 34.08 ± 13.79 | 37.55 ± 11.72 | 30.8 ± 13.89 | 0.1052 |
| AST (U/L) | 25.23 ± 7.76 | 25.29 ± 6.90 | 25.18 ± 8.57 | 0.9655 | 29.20 ± 9.43 | 30.90 ± 8.61 | 27.50 ± 9.06 | 0.2315 |
| HbA1c (%) | 5.31 ± 0.24 | 5.34 ± 0.23 | 5.28 ± 0.23 | 0.4780 | 5.62 ± 0.36 | 5.67 ± 0.34 | 5.54 ± 0.29 | 0.2034 |

BMI, body mass index; FPG, fasting plasma glucose; 2hPG, 2-h plasma glucose during OGTT; TC, Total Cholesterol; TG, Triglycerides; HDL-C, high-density lipoprotein cholesterol; LDL-C, low-density lipoprotein cholesterol; ALT, alanine transaminase; AST, aspartate transaminase; Data are shown as mean ± SEM.

**Table S3 Primer sequences for qRT-PCR**

| Gene Name | Species | Forward Sequence | Reverse Sequence |
| --- | --- | --- | --- |
| *GAPDH* | Mouse | AGGTCGGTGTGAACGGATTTG | GGGGTCGTTGATGGCAACA |
| *SI* | Mouse | CGTTTCCGGTTCAAGCTCACA | CCTGATGACTTTGATGCTGAACG |
| *MGAM* | Mouse | GCCACCTGGTACGACTATGAA | TTGTGTGGGAAAGATGTAGCC |
| *MUC2* | Mouse | TTTCAAGCACCCCTGTAACC | AGGTCCTGGTGTTGAACCTG |
| *GAPDH* | Human | CCACCCATGGCAAATTCC | TGGGATTTCCATTGATGACAAG |
| *SI* | Human | CGACGGAGAAGCAGTGGAAG | GGGTTGGTCTCTTGTGAACATTC |
| *MUC2* | Human | CAGCACCGATTGCTGAGTTG | GCTGGTCATCTCAATGGCAG |

**Supplementary Figures**

**
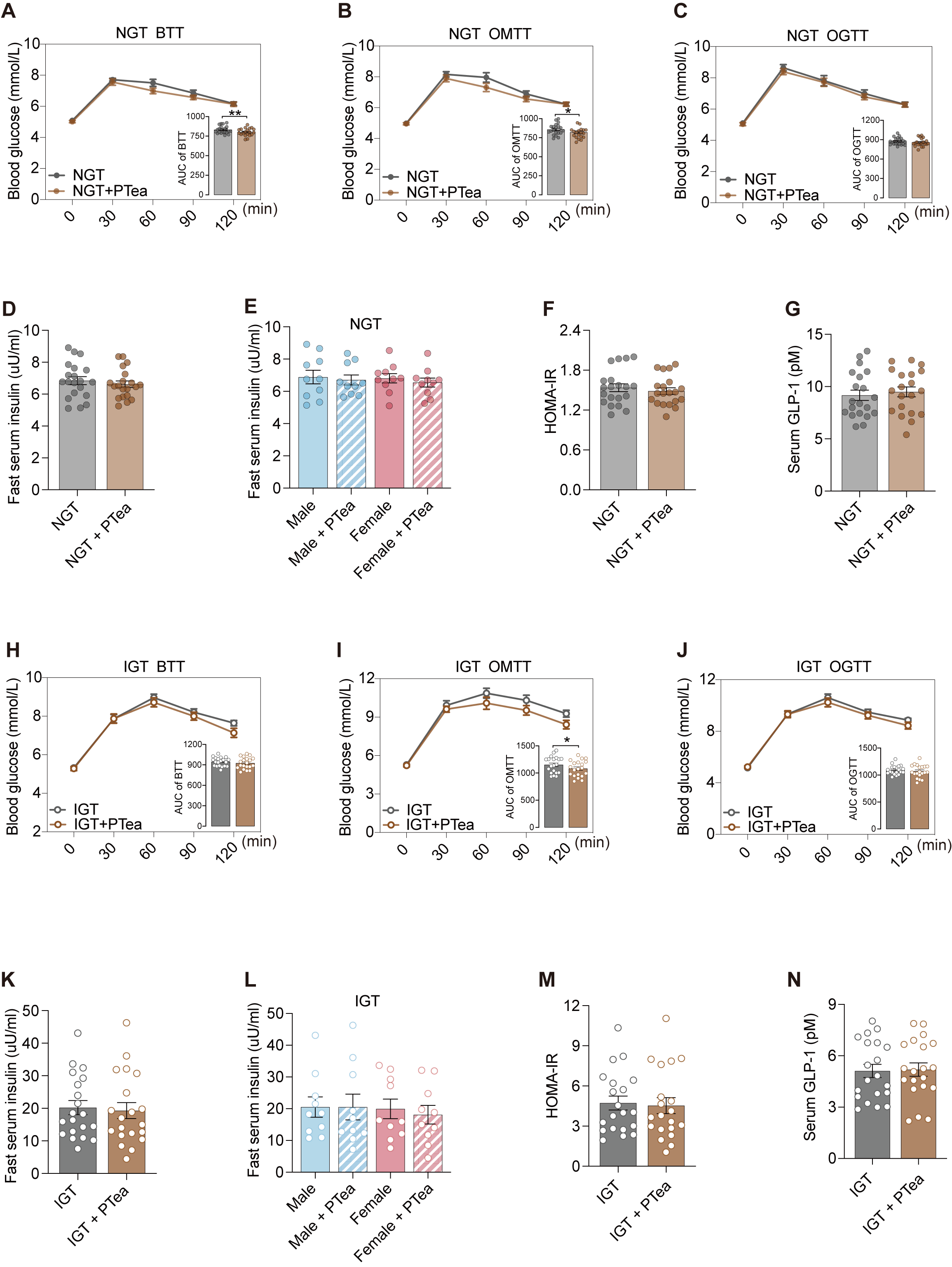
**

**FIGURE S1.** Effects of Pu-erh tea on the regulation of glucose homeostasis in the NGT and IGT individuals. (A-G) (A) Blood glucose levels and AUC of BTT, (B) blood glucose levels and AUC of OMTT, (C) blood glucose levels and AUC of OGTT, (D-E) Fast serum insulin (FINS), (F) HOMA-IR and (G) serum active GLP-1 levels in the NGT individuals (n = 20). (H-N) (H) Blood glucose levels and AUC of BTT, (I) blood glucose levels and AUC of OMTT, (J) blood glucose levels and AUC of OGTT, (K-L) Fast serum insulin (FINS), (M) HOMA-IR and (N) serum active GLP-1 levels in the IGT individuals (n = 20). Data are presented as mean ± SEM. *p < 0.05, **p < 0.01 compared with the Pu-erh tea groups, based on the Wilcoxon matched-pairs signed rank test.


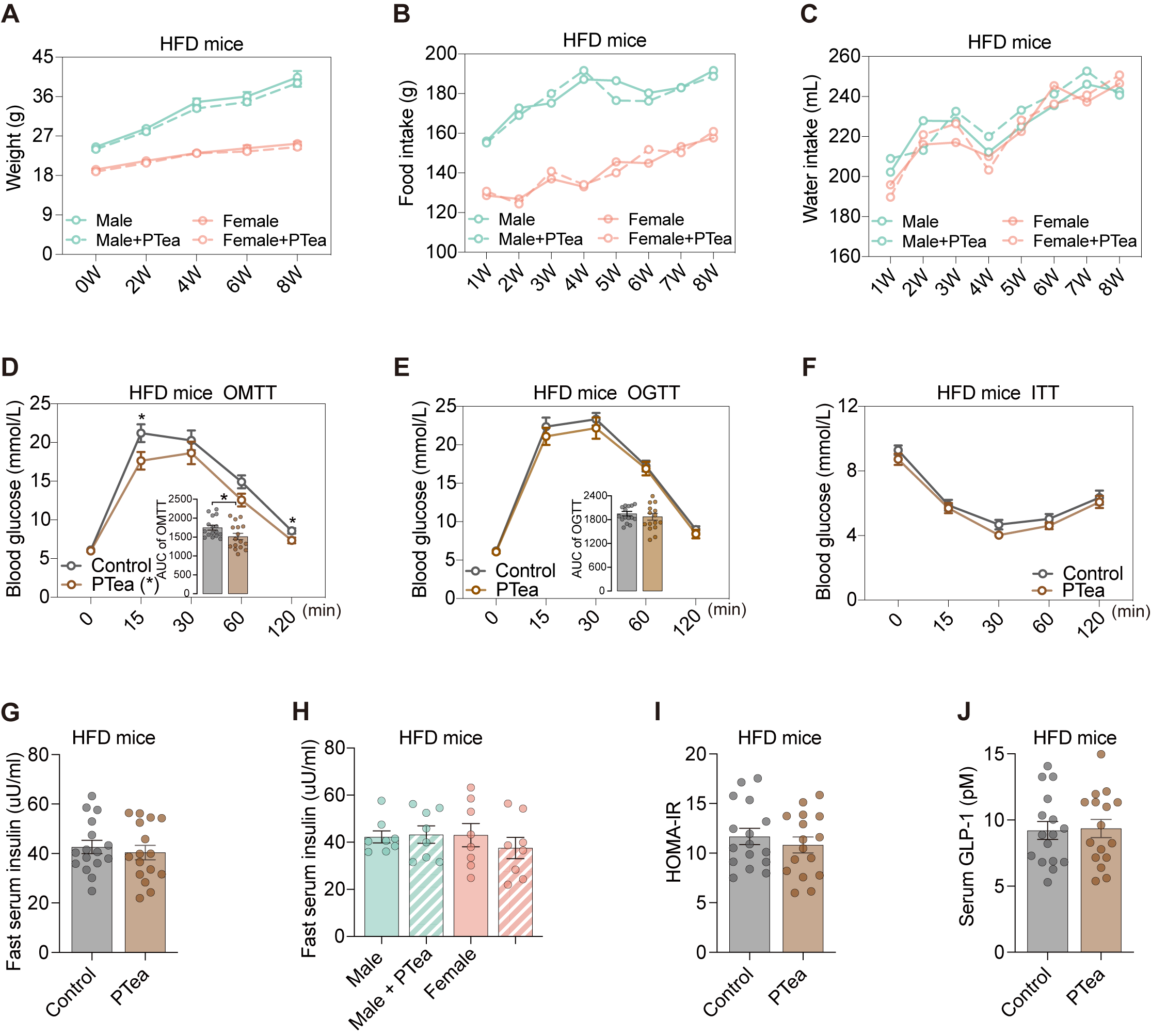


**FIGURE S2.** Effects of Pu-erh tea on blood glucose levels in the HFD. (A-C) Parameters measured in the four groups after 8 weeks of treatment: body weight (A), food intake (B) and water intake (C). (D-J) (D) Blood glucose levels and AUC of OMTT, (E) blood glucose levels and AUC of OGTT, (F) blood glucose levels of ITT, (G-H) Fast serum insulin (FINS), (I) HOMA-IR and (J) serum active GLP-1 levels in the HFD mice (n = 16/group). Data are shown as mean ± SEM. *p < 0.05 and **p < 0.01 compared between groups, based on the Mann-Whitney U test.


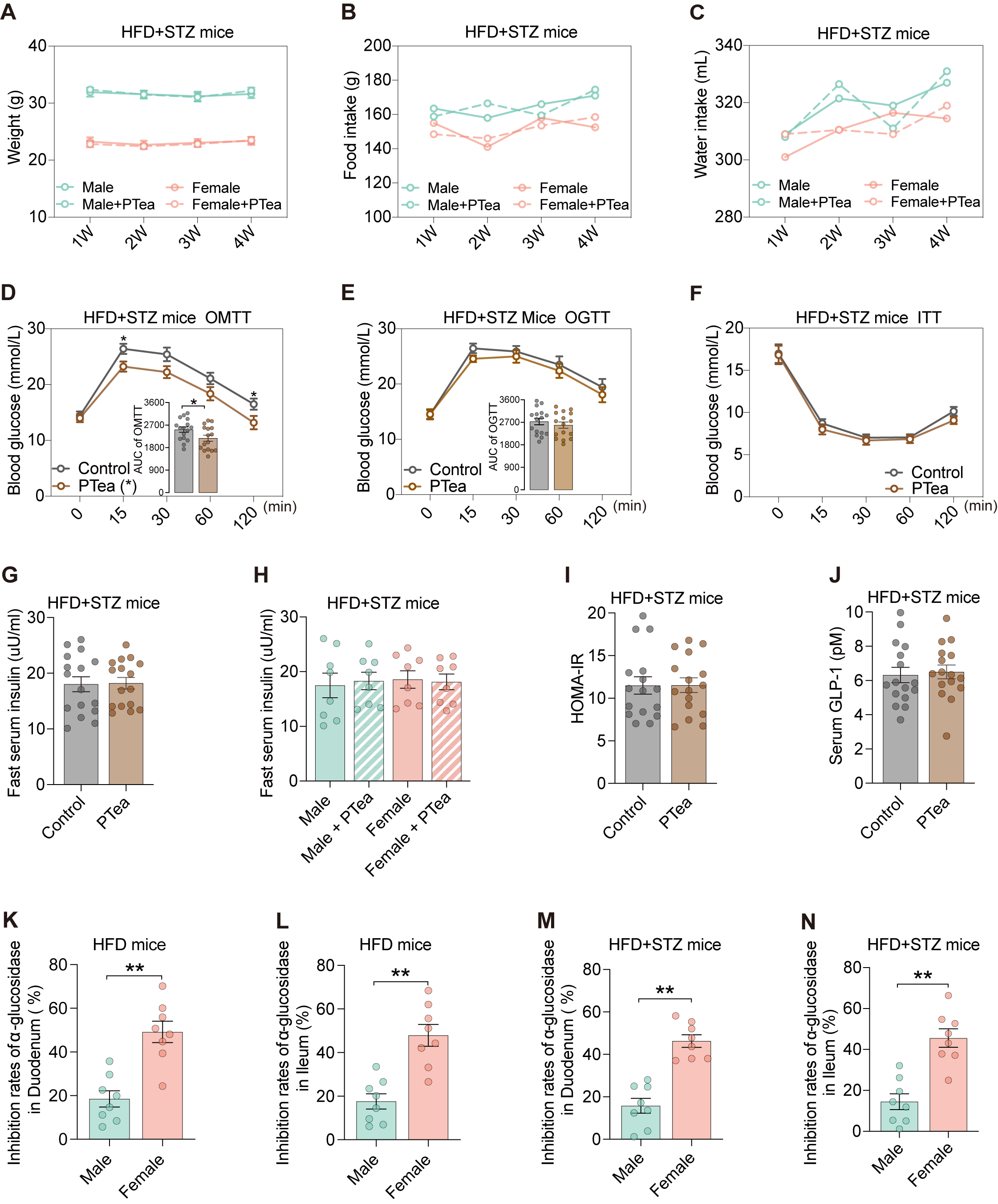


**FIGURE S3.** Effects of Pu-erh tea on blood glucose levels in the HFD+STZ. (A-C) Parameters measured in the four groups after 8 weeks of treatment: body weight (A), food intake (B) and water intake (C). (D-J) (D) Blood glucose levels and AUC of OMTT, (E) blood glucose levels and AUC of OGTT, (F) blood glucose levels of ITT, (G-H) fast serum insulin (FINS), (I) HOMA-IR and (J) serum active GLP-1 levels in the HFD+STZ mice. (K-L) Inhibition rates of α-glucosidase in the duodenum (K) and ileum (L) of the HFD mice (n = 16/group). (M-N) Inhibition rates of α-glucosidase in the duodenum (M) and ileum (N) of the HFD+STZ mice. Data are shown as mean ± SEM. *p < 0.05 and **p < 0.01 compared between groups, based on the Mann-Whitney U test.


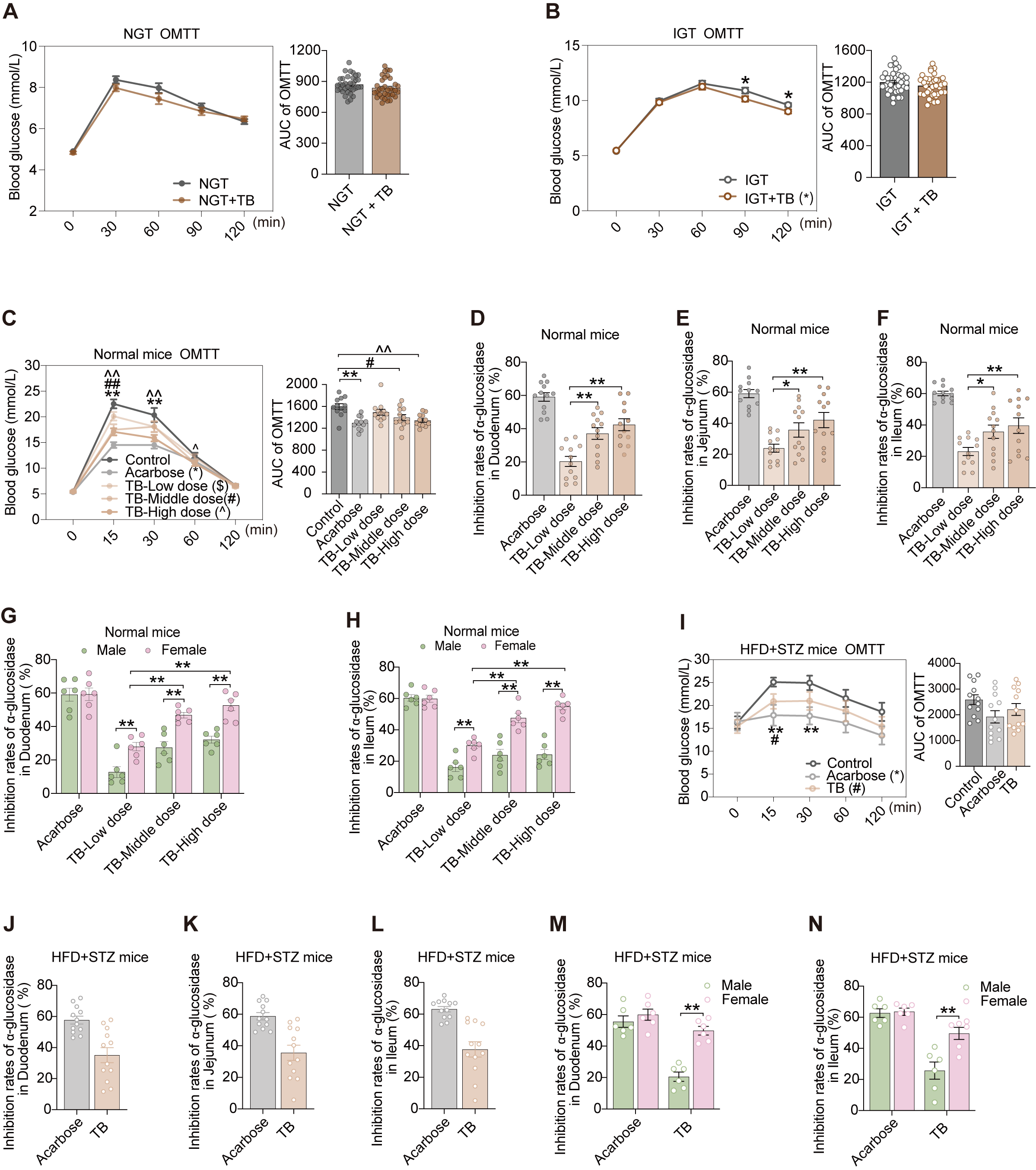


**FIGURE S4.** Effects of TB on the regulation of α-glucosidase *in vivo.* (A-B) Blood glucose levels and AUC of OMTT in the NGT individuals (A) and in the IGT individuals (B). (C-H) (C) Blood glucose levels and AUC of OMTT, (D-H) Inhibition rates of α-glucosidase in the duodenum, jejunum and ileum of the normal mice (n = 12/group). (I-N) (I) Blood glucose levels and AUC of OMTT, (J-N) inhibition rates of α-glucosidase in the duodenum, jejunum and ileum of the HFD+STZ mice (n = 12/group). Data are shown as mean ± SEM. #p < 0.05 and **, ##,^^p < 0.01 in (C) and (I) compared between groups, based on the Kruskal-Wallis test with Benjamini Hochberg adjustment; *p < 0.05 and **p < 0.01 in (D-H), (K) and (N) compared between groups, based on the Mann-Whitney U test and the Kruskal-Wallis test with Benjamini Hochberg adjustment.


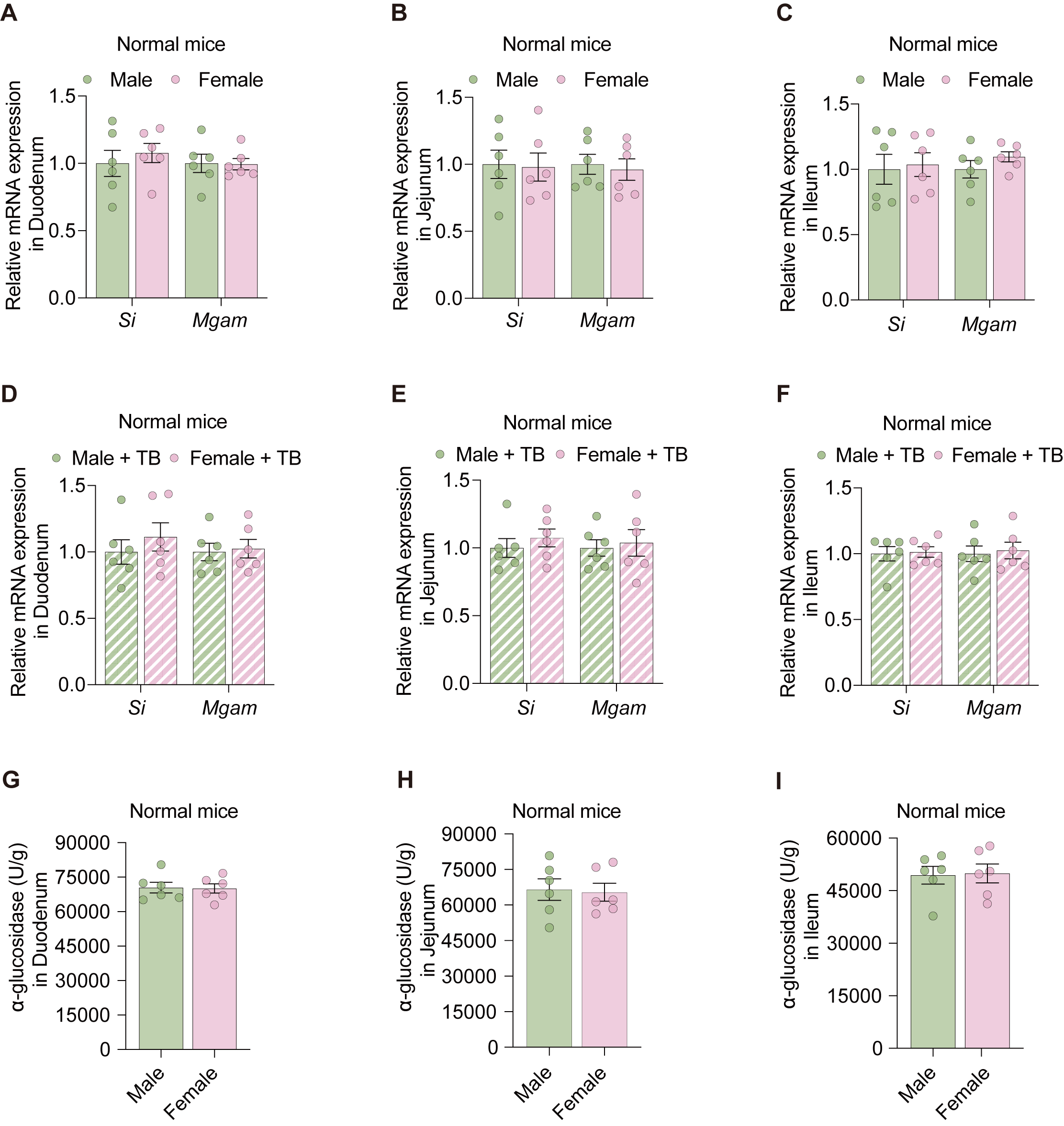


**FIGURE S5.** Gene expression and activity of α-glucosidase showed no sex differences. (A-C) The relative mRNA levels of *Si* and *Mgam* in the duodenum (A), jejunum (B), and ileum (C) tissues of the male and female normal mice (n = 6/group). (D-F) The relative mRNA levels of *SI* and *MGAM* in the duodenum (D), jejunum (E), and ileum (F) tissues of the male and female normal mice with TB (n = 6/group). (G-I) The levels of α-glucosidase in the duodenum (G), jejunum (H), and ileum (I) tissues of the male and female normal mice with TB (n = 6/group).

**
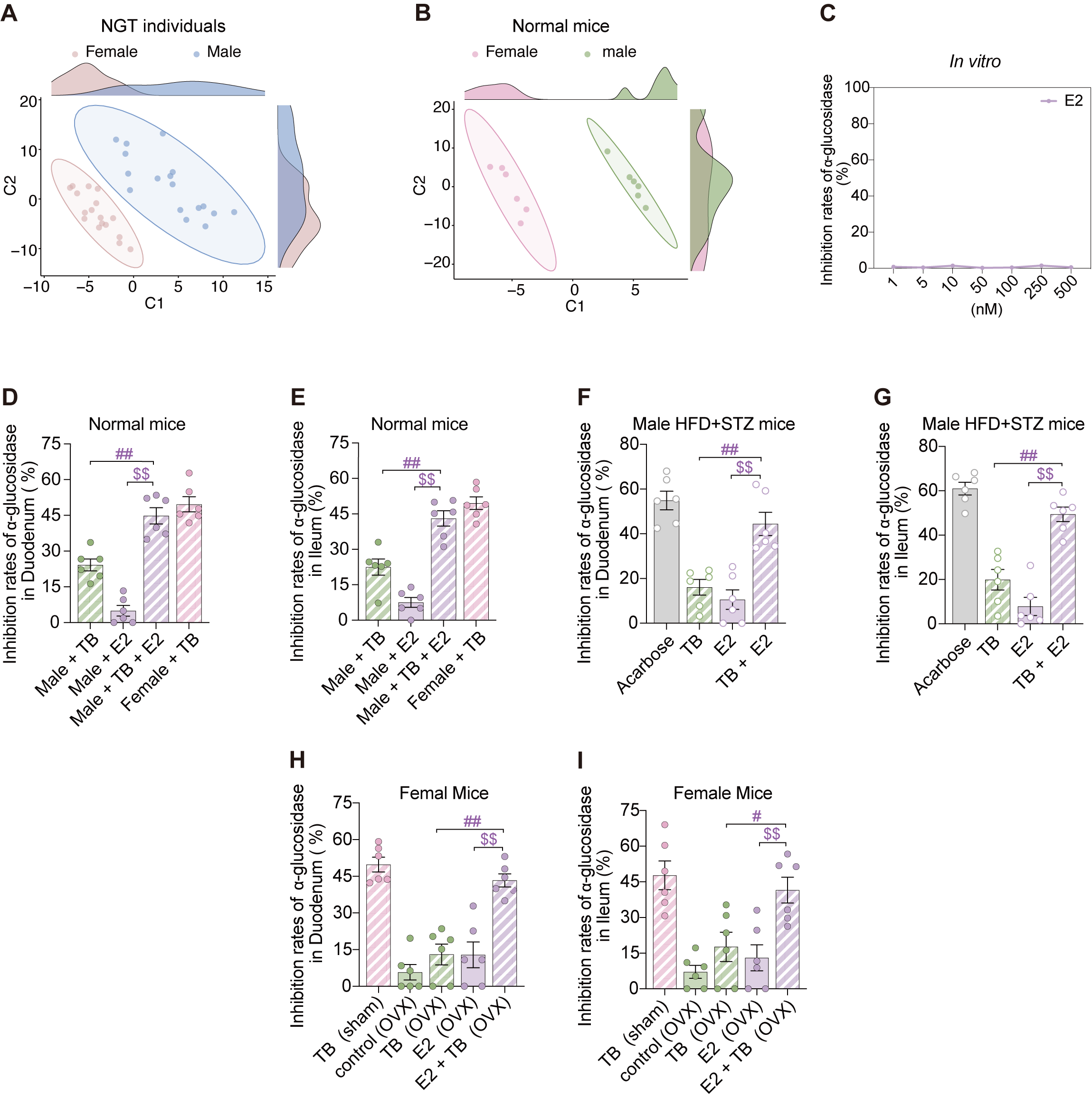
FIGURE S6.** Estradiol facilitated TB's Inhibition of α-glucosidase in the duodenum and ileum of mice. (A) PLS-DA scores plot based on fecal metabolite abundance in 17 male vs 17 female NGT individuals. (B) PLS-DA scores plot based on metabolite abundance from feces in 6 normal male vs 6 female mice. (C) Inhibitory effects of E2 of different concentration gradients on α-glucosidase *in vitro*. (D-E) Inhibition rates of α-glucosidase in the duodenum (D) and ileum (E) of the male mice plus E2. (F-G) Inhibition rates of α-glucosidase in the duodenum (F) and ileum (G) of the male HFD+STZ mice. (H-I) Inhibition rates of α-glucosidase in the duodenum (H) and ileum (I) of the ovariectomized female mice. Data are shown as mean ± SEM. #p < 0.05 and ##p < 0.01 compared with male TB and OVX TB groups, respectively; $p < 0.05 and $$p < 0.01 and compared with male E2 and OVX E2 groups, respectively, based on the Kruskal-Wallis test with Benjamini Hochberg adjustment.


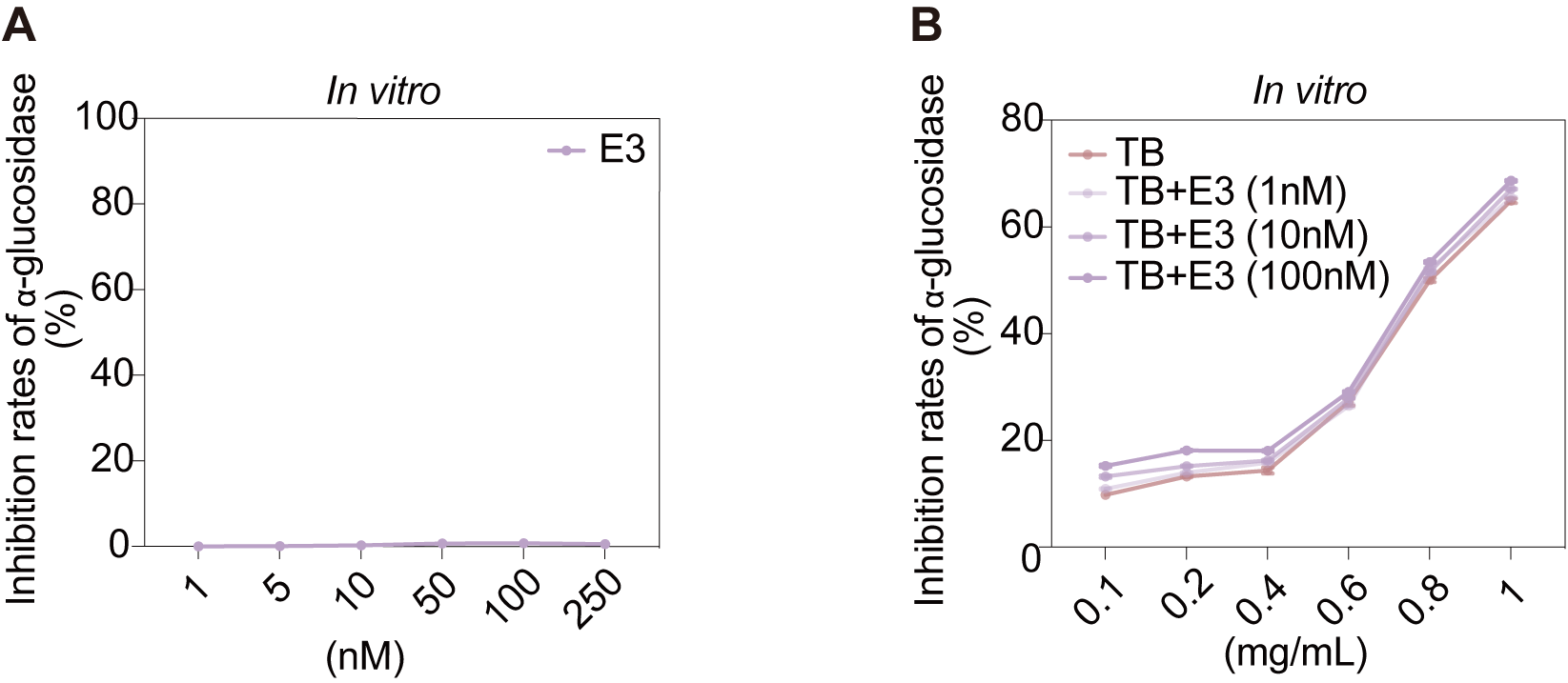


**FIGURE S7.** Inhibition of α-glucosidase by the combination of TB and estriol *in vitro.* (A) Inhibitory effects of estriol (E3) of different concentration gradients on α-glucosidase *in vitro*. (B) Inhibitory effects of TB, TB+E3 (1 nM), TB+E3 (10 nM), and TB+E3 (100 nM) on α-glucosidase *in vitro*.

**
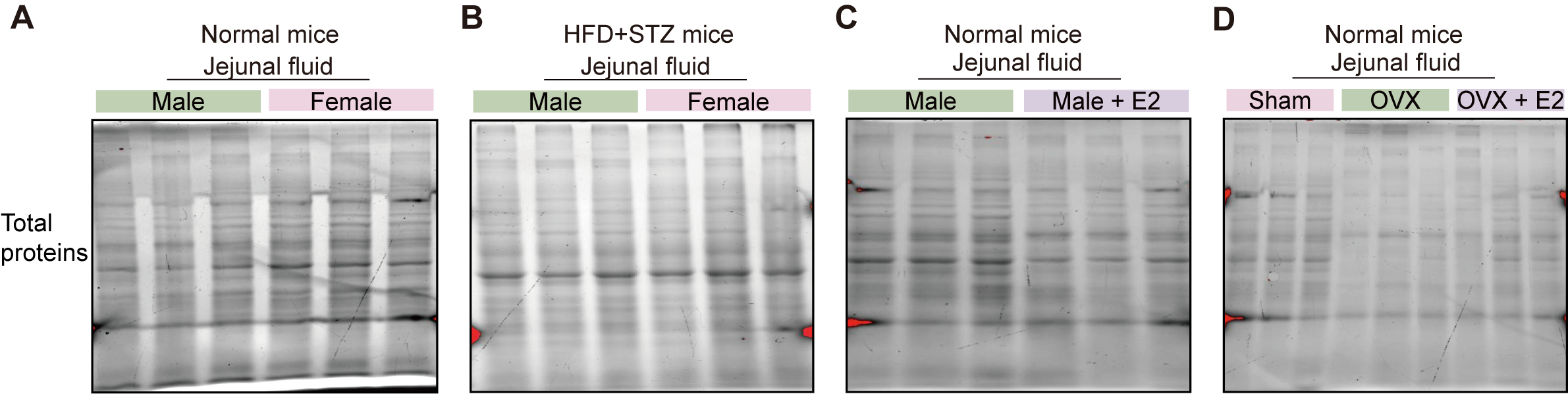
FIGURE S8.** The total protein expression of MUC2 in the jejunal fluid of the mice. (A-D) The total protein expression of MUC2 in the jejunal fluid of the normal mice (A), the HFD+STZ mice (B), the male normal mice plus E2 (C), and the female mice of ovariectomy (D) (n = 3/group)

**
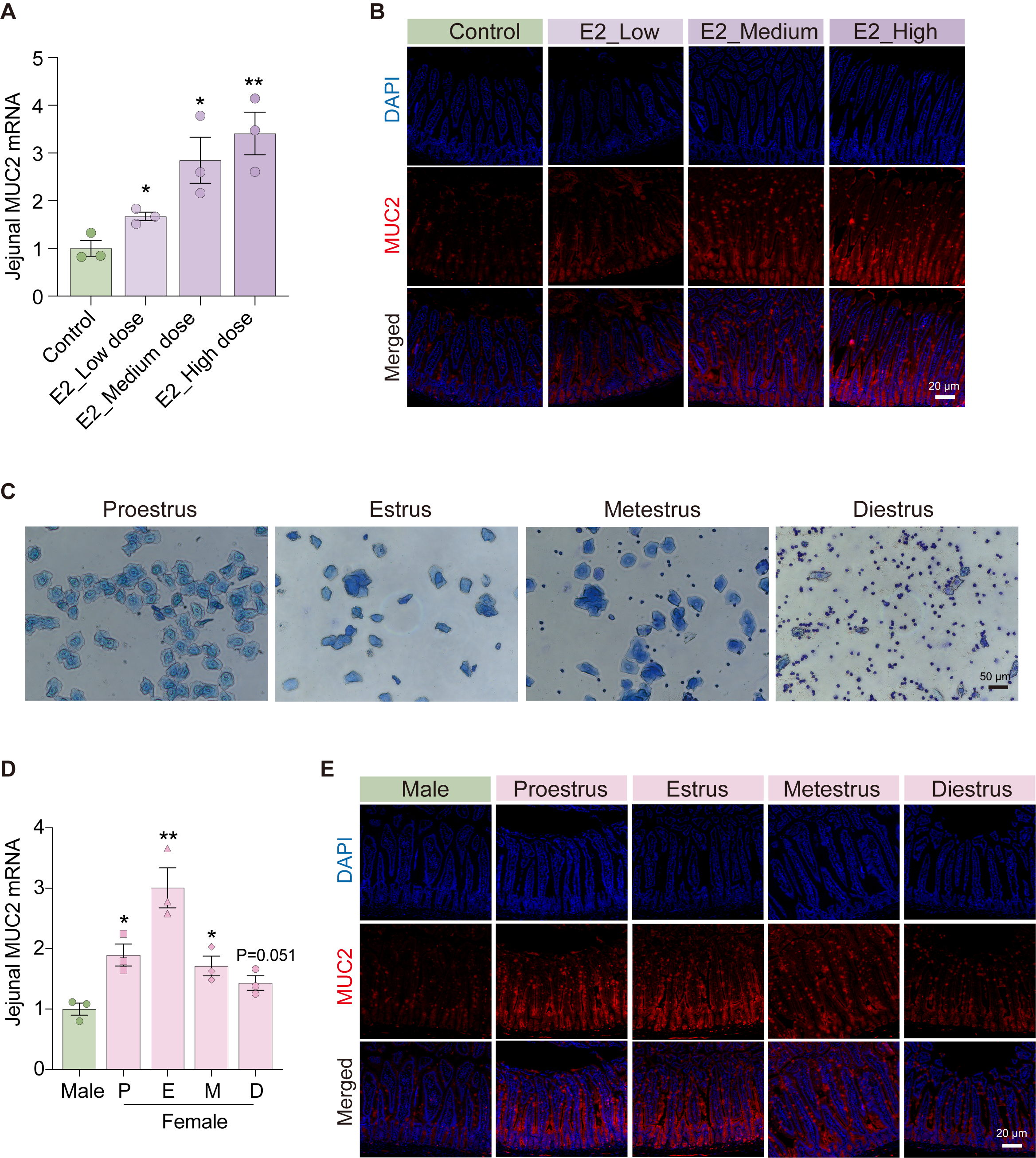
FIGURE S9.** The expression of MUC2 depended on the E2 dose and the estrous phase in the mice. (A-B) The relative mRNA levels (A) and immunofluorescence staining of MUC2 (B) in the jejunal tissues of the normal male mice with different doses of estradiol (E2) (n = 3/group). (C) Vaginal cytology of female mice (n = 3/group). (D-E) The relative mRNA levels (P: Proestrus; E: Estrus; M: Metestrus; D: Diestrus) (D) and immunofluorescence staining of MUC2 (E) in the jejunal tissues of the normal male and female mice (n = 3/group). Data are shown as mean ± SEM. *p < 0.05 and **p < 0.01 compared with control or male groups based on the Kruskal-Wallis test with Benjamini Hochberg adjustment.

Animal experiment S1: E2 intervention in normal male mice

Mice were randomly divided into four groups with 3 in each group : (1) Control group: mice were administered intragastrically with phosphate-buffered saline (PBS); (2) E2-Low dose group: mice were orally administered with E2 at a dose of 0.6 ng/mouse; (3) E2-Middle dose group: mice were orally administered with E2 at a dose of 3 ng/mouse; (4) E2-High dose group: mice were orally administered with E2 at a dose of 6 ng/mouse. After a 1-week interval, each group of mice was fasted overnight and then orally administered either PBS or acarbose or doses of E2 (low, middle, or high), or PBS. The mice were euthanized for collecting serum, intestinal tissues and contents 45 minutes later.

Animal experiment S2: Estrous cycle in normal female mice

The vaginal cells were collected from female mice via saline lavage and stained with modified Giemsa solution to track the estrous cycles of all twelve mice. Images were captured at 20× and analyzed. These female mice were stratified into four groups with 3 in each group according to distinct estrous cycle phases: proestrus, estrus, metestrus and diestrus. Three normal male mice were included as the control group. The mice were euthanized for collecting serum, intestinal tissues and contents.

**
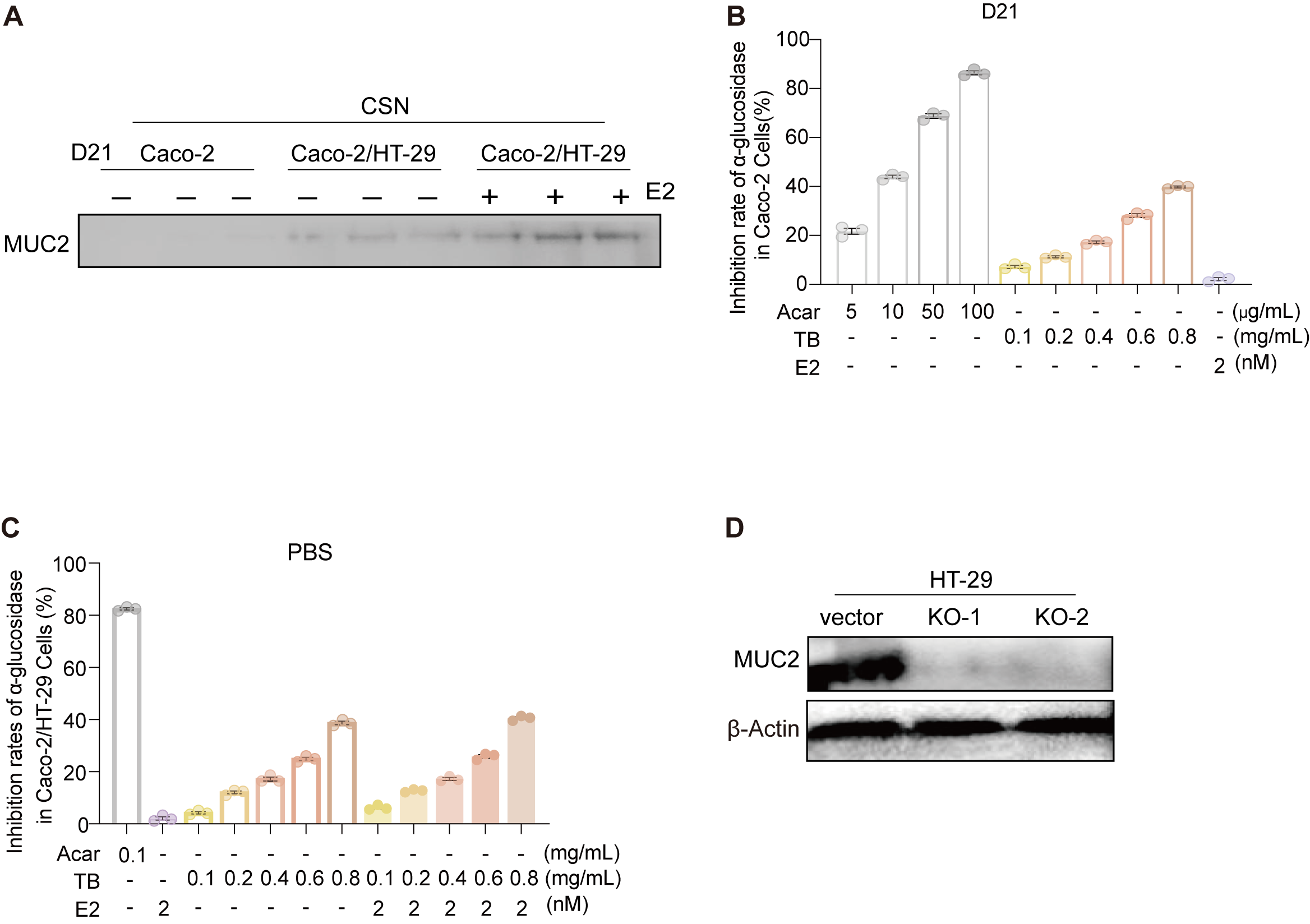
FIGURE S10.** Gene expression and activity of α-glucosidase were examined in Caco-2 and Caco-2/HT-29 cells at different times. (A) The protein expressions of MUC2 in the cell supernatants (CSNs) of Caco-2, the Caco-2/HT-29 and Caco-2/HT-29 plus E2. (B) Inhibitory effects of TB and TB plus MUC2 of different concentration gradients on α-glucosidase in Caco-2 cells on the 21st day. (C) Inhibitory effects of Acarbose, E2, TB of different concentration gradients, and TB plus E2 of different concentrations on α-glucosidase in Caco-2/HT-29 cells with PBS. (D) The MUC2 expression in (MUC2-/-) HT-29. Data are shown as mean ± SEM.


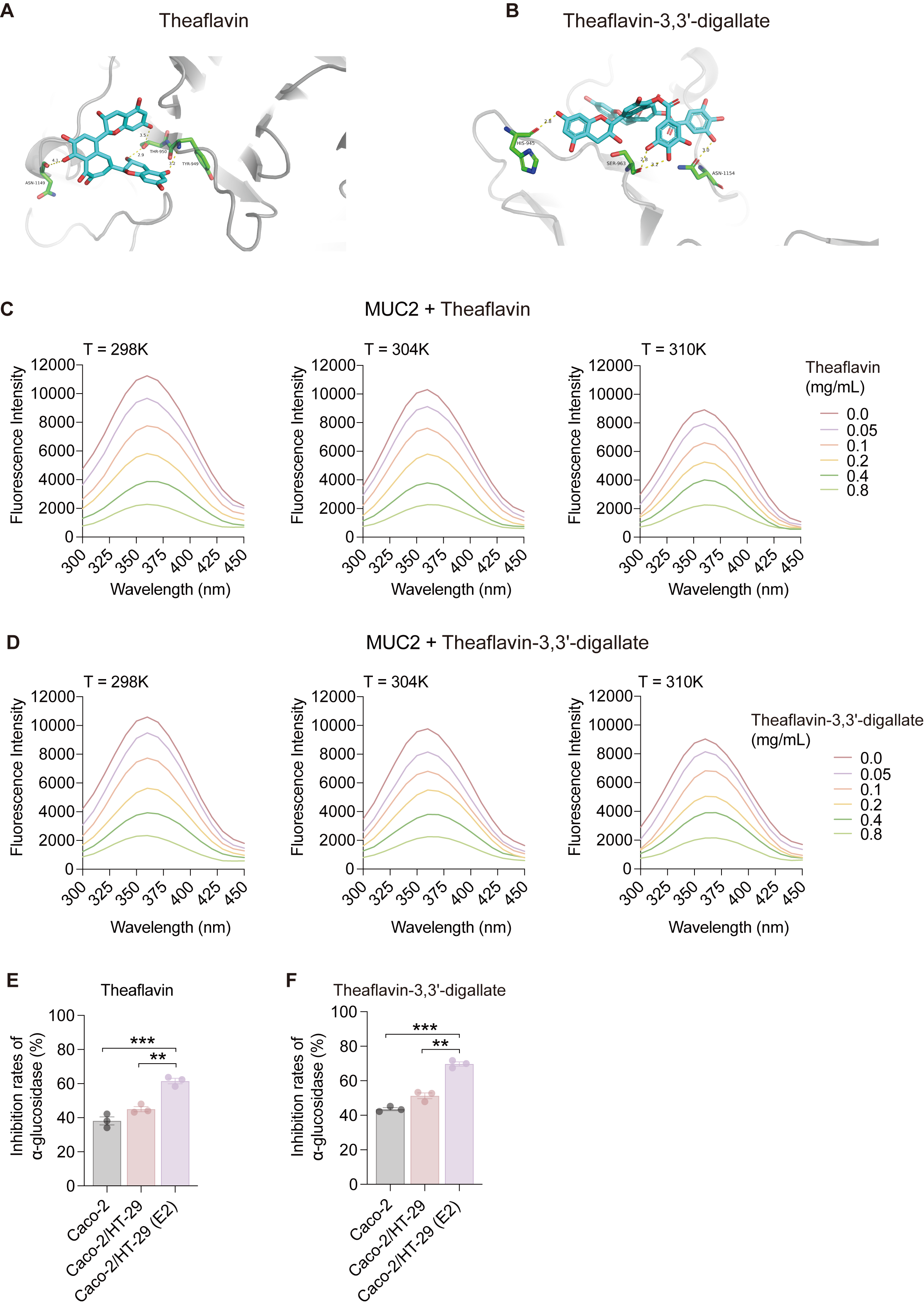


**FIGURE S11.** Theaflavins-MUC2 interaction *in vitro* and in cells. (A-B) Molecular docking of MUC2 to theaflavin (A) and theaflavin-3,3'-digallate (B), respectively. (C) Fluorescence spectra of MUC2 (80 μg/mL) at different concentrations of theaflavin at 298 K, 304 K, and 310 K. (D) Fluorescence spectra of MUC2 (80 μg/mL) at different concentrations of theaflavin-3,3'-digallate at 298 K, 304 K, and 310 K. (E) Inhibitory effects of theaflavin (0.8 mg/mL) and theaflavin (0.8 mg/mL) plus E2 (2nM) on α-glucosidase in Caco-2 and Caco-2/HT-29 cells. (F) Inhibitory effects of theaflavin-3,3'-digallate (0.8 mg/mL) and theaflavin-3,3'-digallate (0.8 mg/mL) plus E2 (2nM) on α-glucosidase in Caco-2 and Caco-2/HT-29 cells.

Molecular docking analysis procedures

We utilized Schrödinger software, a computational tool for quantifying ligand binding

free energy and predicting ligand orientation and conformation within the target binding site to perform molecular docking analysis.
