# Supplementary figures and images for: Sex‐Specific Regulation of Glycemic Homeostasis by Theabrownin from Pu‐erh Tea
Source: Adv Sci (Weinh). 2026 Apr 16;13(38):e19337. doi: 10.1002/advs.202519337 (PMC13335617; doi:10.1002/advs.202519337)

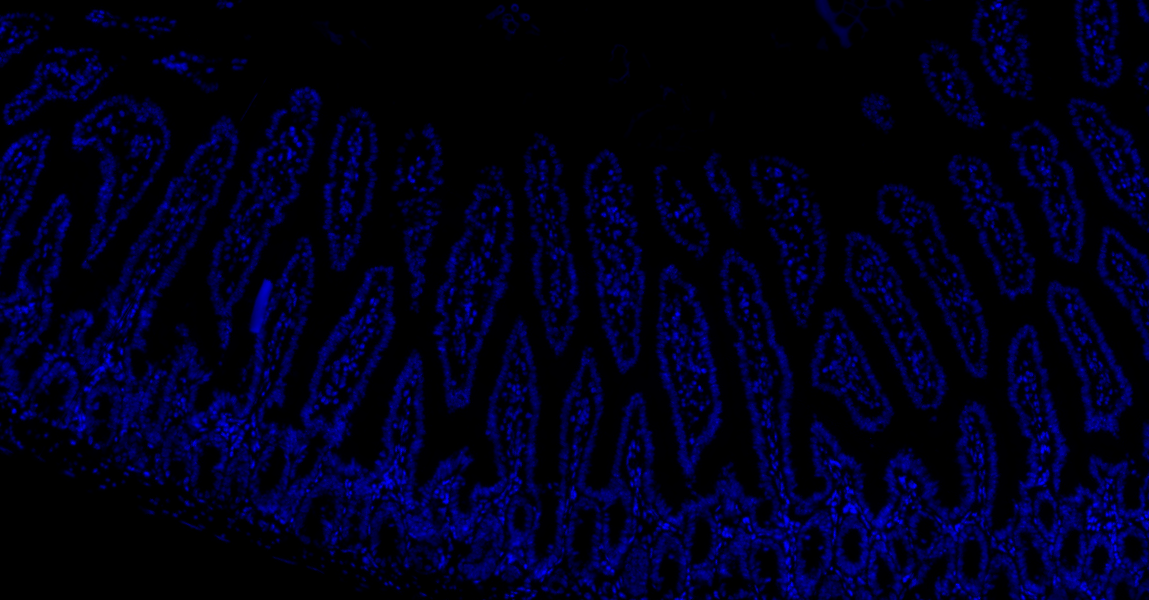

Supplement: Supplementary file 2 — Supporting File: advs75314‐sup‐0002‐RawData.zip. [file ADVS-13-e19337-s001.zip › Control DAPI.tif]

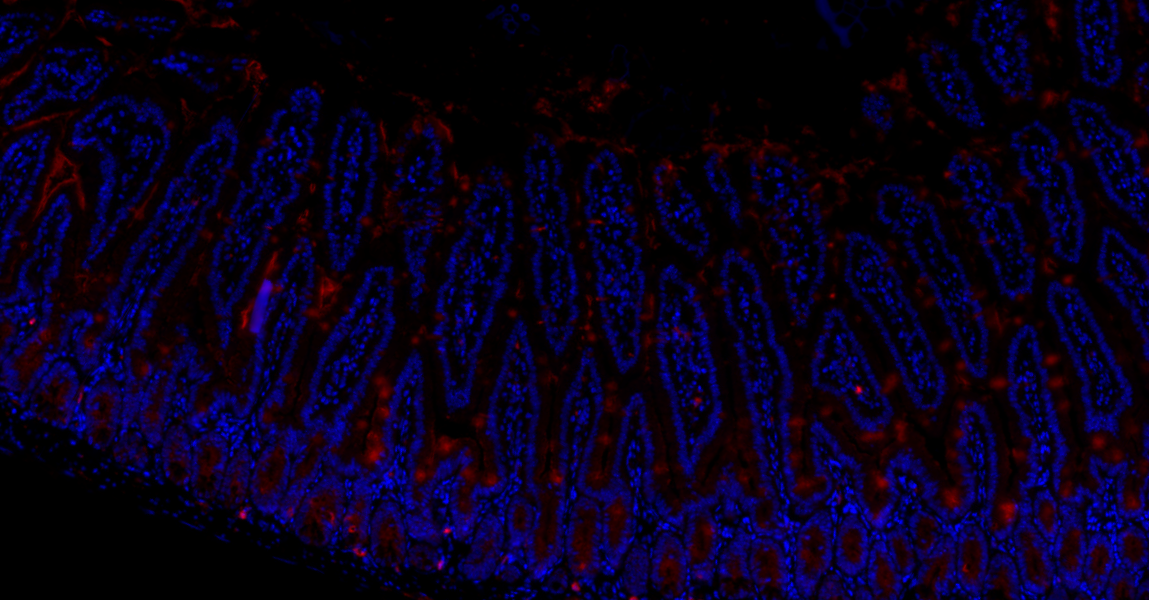

Supplement: Supplementary file 2 — Supporting File: advs75314‐sup‐0002‐RawData.zip. [file ADVS-13-e19337-s001.zip › Control Merged.tif]

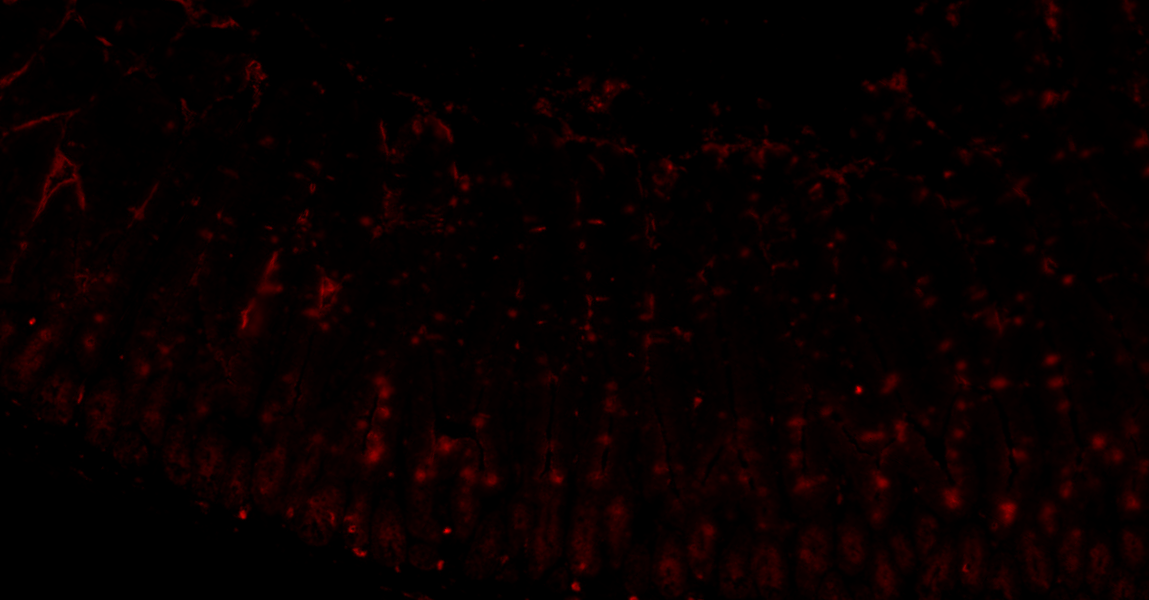

Supplement: Supplementary file 2 — Supporting File: advs75314‐sup‐0002‐RawData.zip. [file ADVS-13-e19337-s001.zip › Control MUC2.tif]

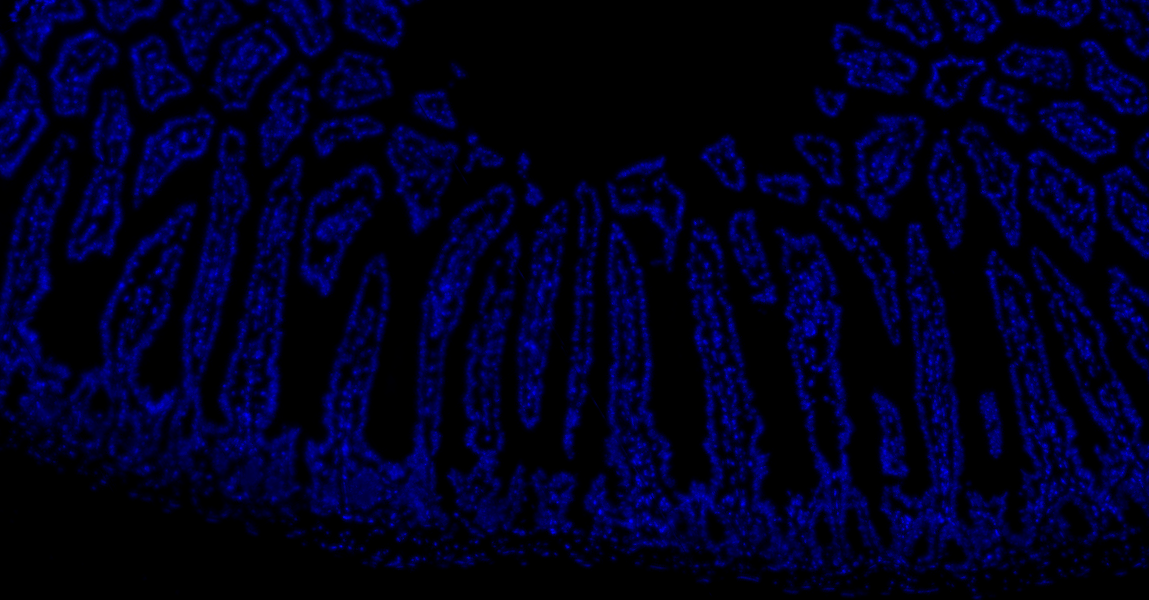

Supplement: Supplementary file 2 — Supporting File: advs75314‐sup‐0002‐RawData.zip. [file ADVS-13-e19337-s001.zip › Diestrus DAPI.tif]

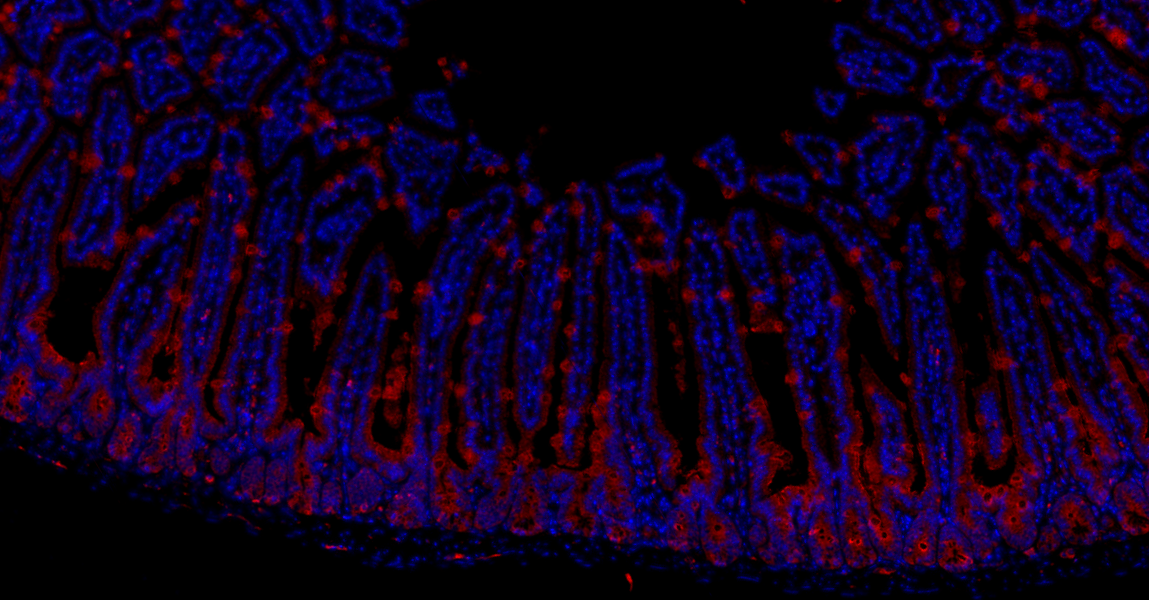

Supplement: Supplementary file 2 — Supporting File: advs75314‐sup‐0002‐RawData.zip. [file ADVS-13-e19337-s001.zip › Diestrus Merged.tif]

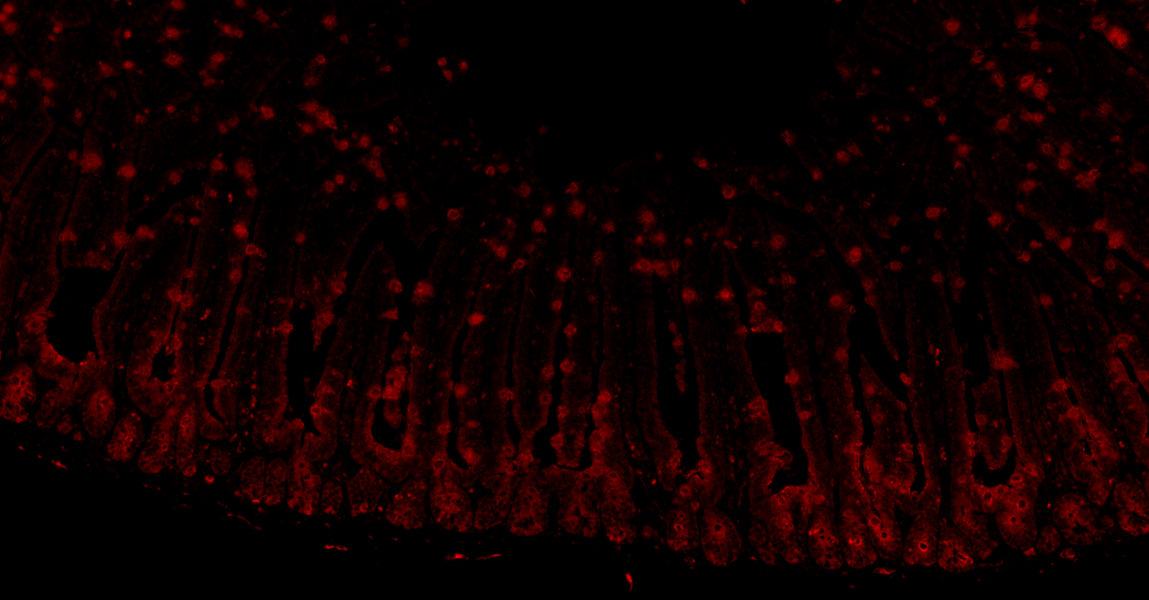

Supplement: Supplementary file 2 — Supporting File: advs75314‐sup‐0002‐RawData.zip. [file ADVS-13-e19337-s001.zip › Diestrus MUC2.tif]

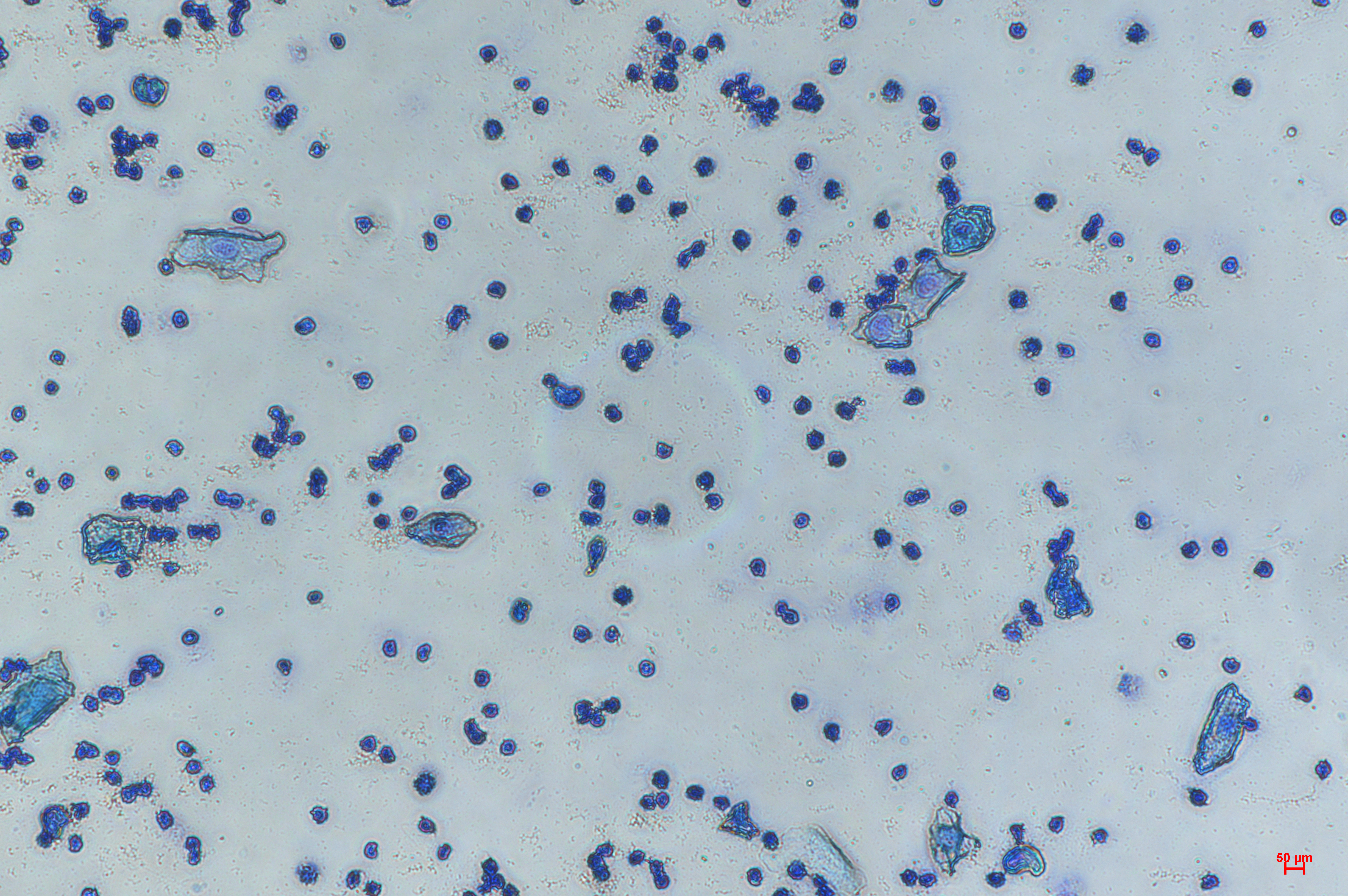

Supplement: Supplementary file 2 — Supporting File: advs75314‐sup‐0002‐RawData.zip. [file ADVS-13-e19337-s001.zip › Diestrus.tif]

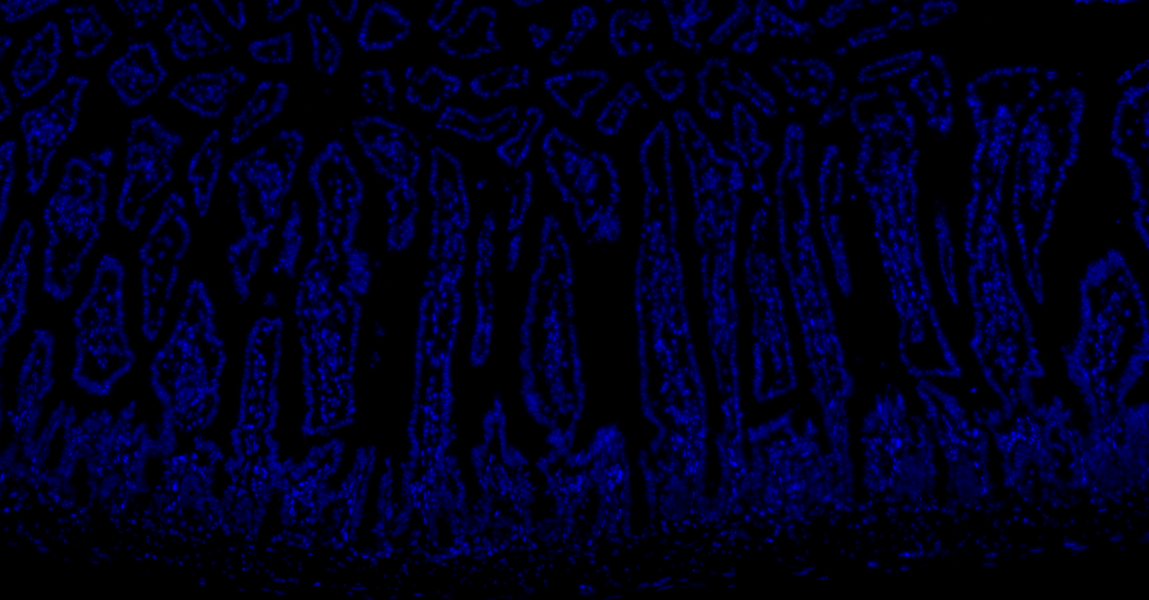

Supplement: Supplementary file 2 — Supporting File: advs75314‐sup‐0002‐RawData.zip. [file ADVS-13-e19337-s001.zip › Estrus DAPI.tif]

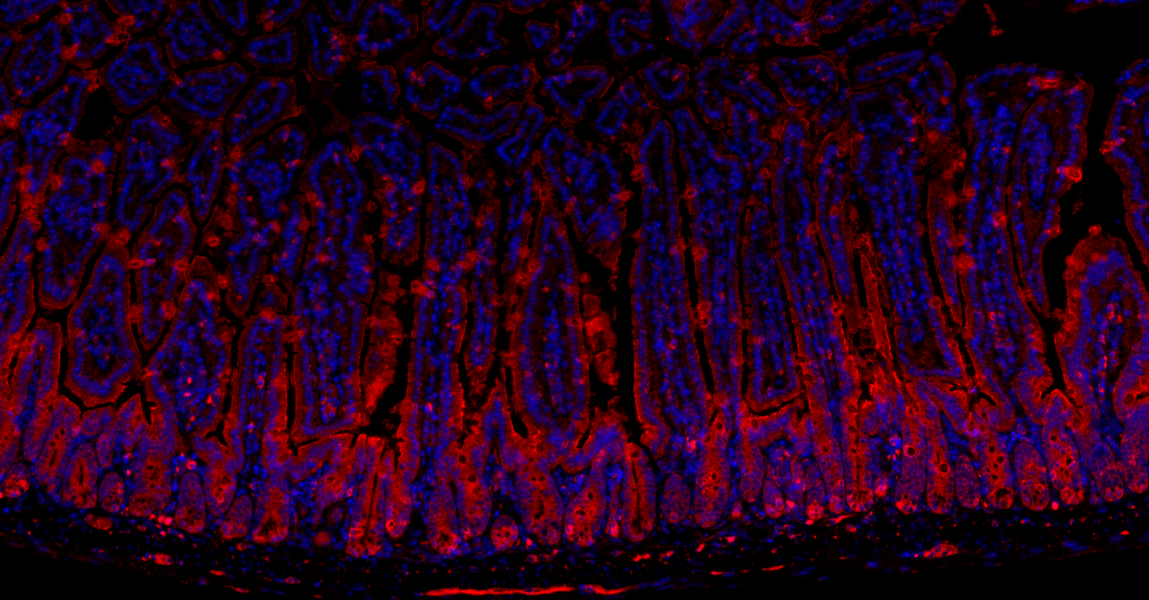

Supplement: Supplementary file 2 — Supporting File: advs75314‐sup‐0002‐RawData.zip. [file ADVS-13-e19337-s001.zip › Estrus Merged.tif]

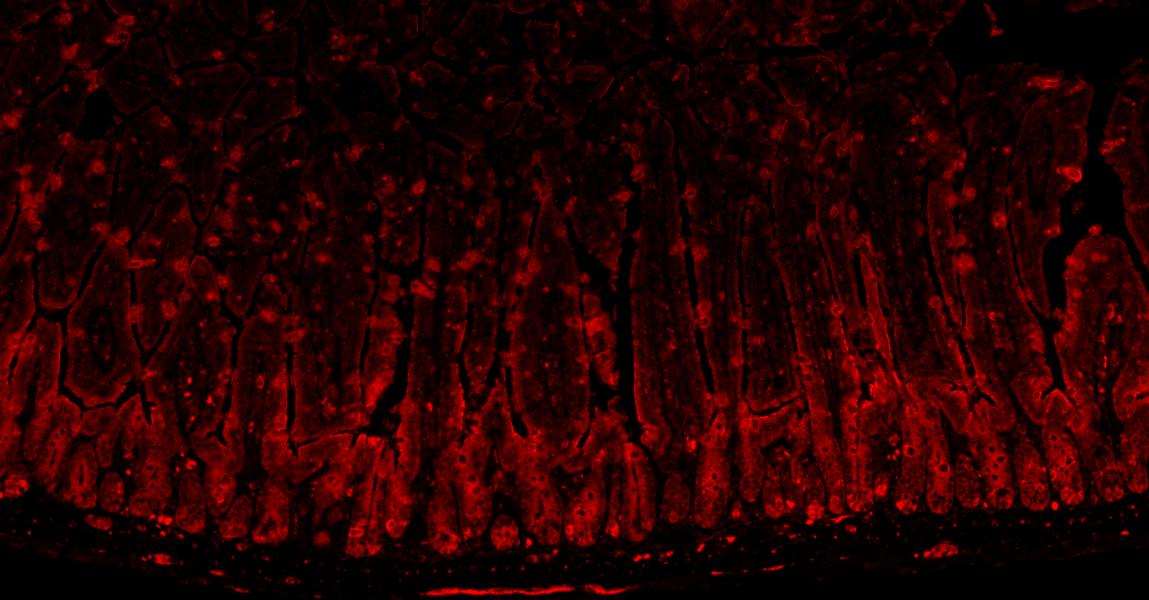

Supplement: Supplementary file 2 — Supporting File: advs75314‐sup‐0002‐RawData.zip. [file ADVS-13-e19337-s001.zip › Estrus MUC2.tif]

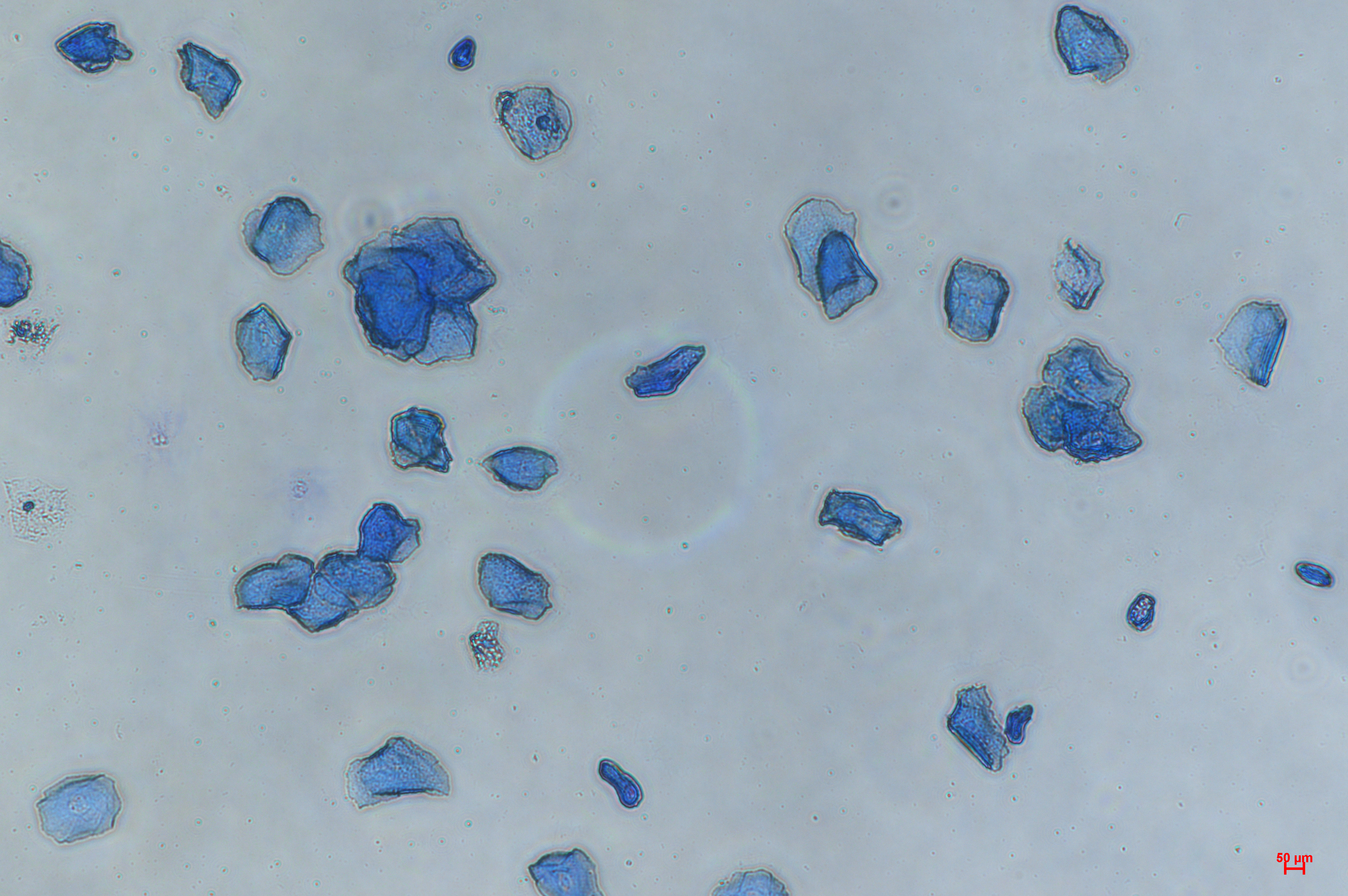

Supplement: Supplementary file 2 — Supporting File: advs75314‐sup‐0002‐RawData.zip. [file ADVS-13-e19337-s001.zip › Estrus.tif]

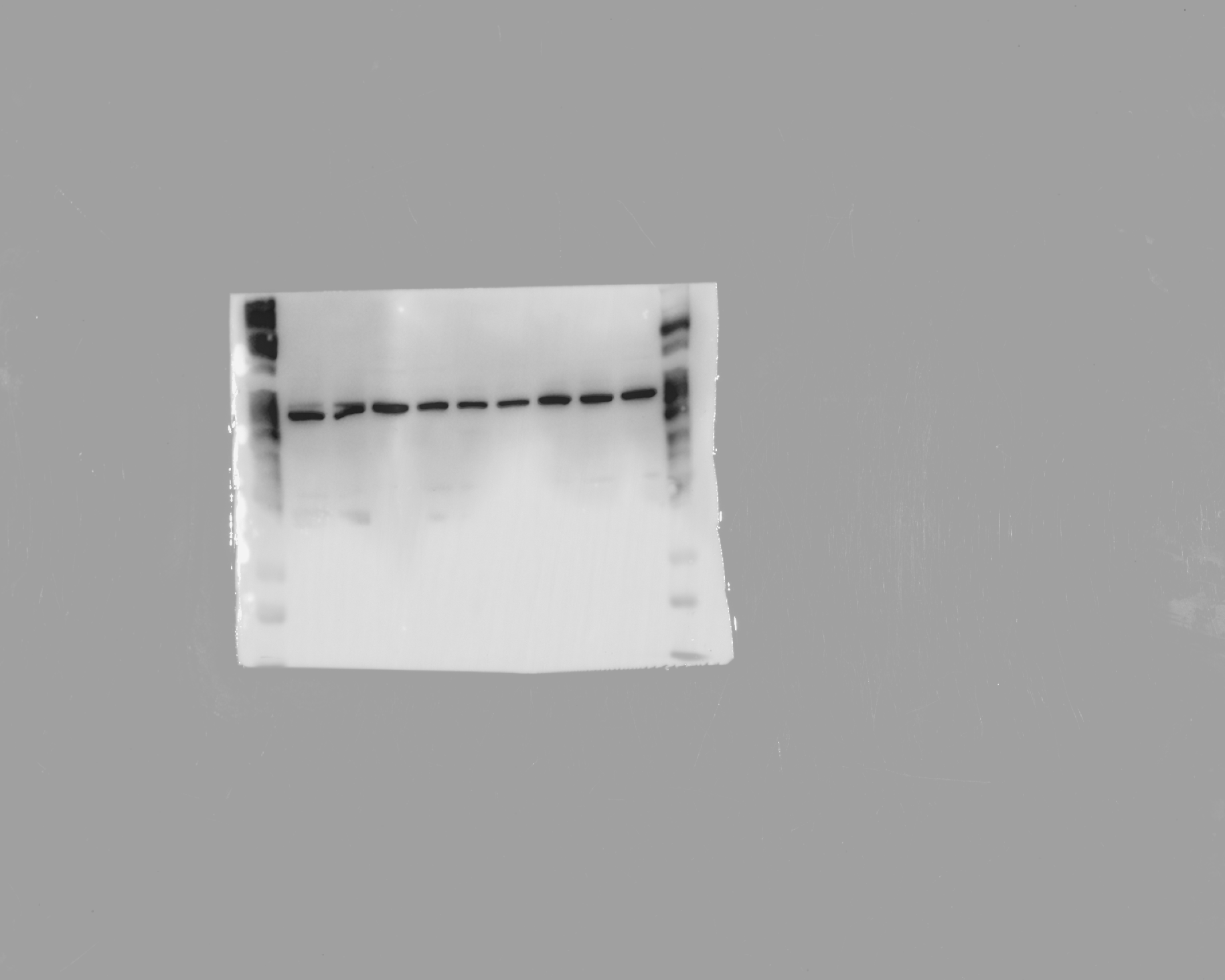

Supplement: Supplementary file 2 — Supporting File: advs75314‐sup‐0002‐RawData.zip. [file ADVS-13-e19337-s001.zip › F ovx jejunal fluid muc2(Composite).tif]

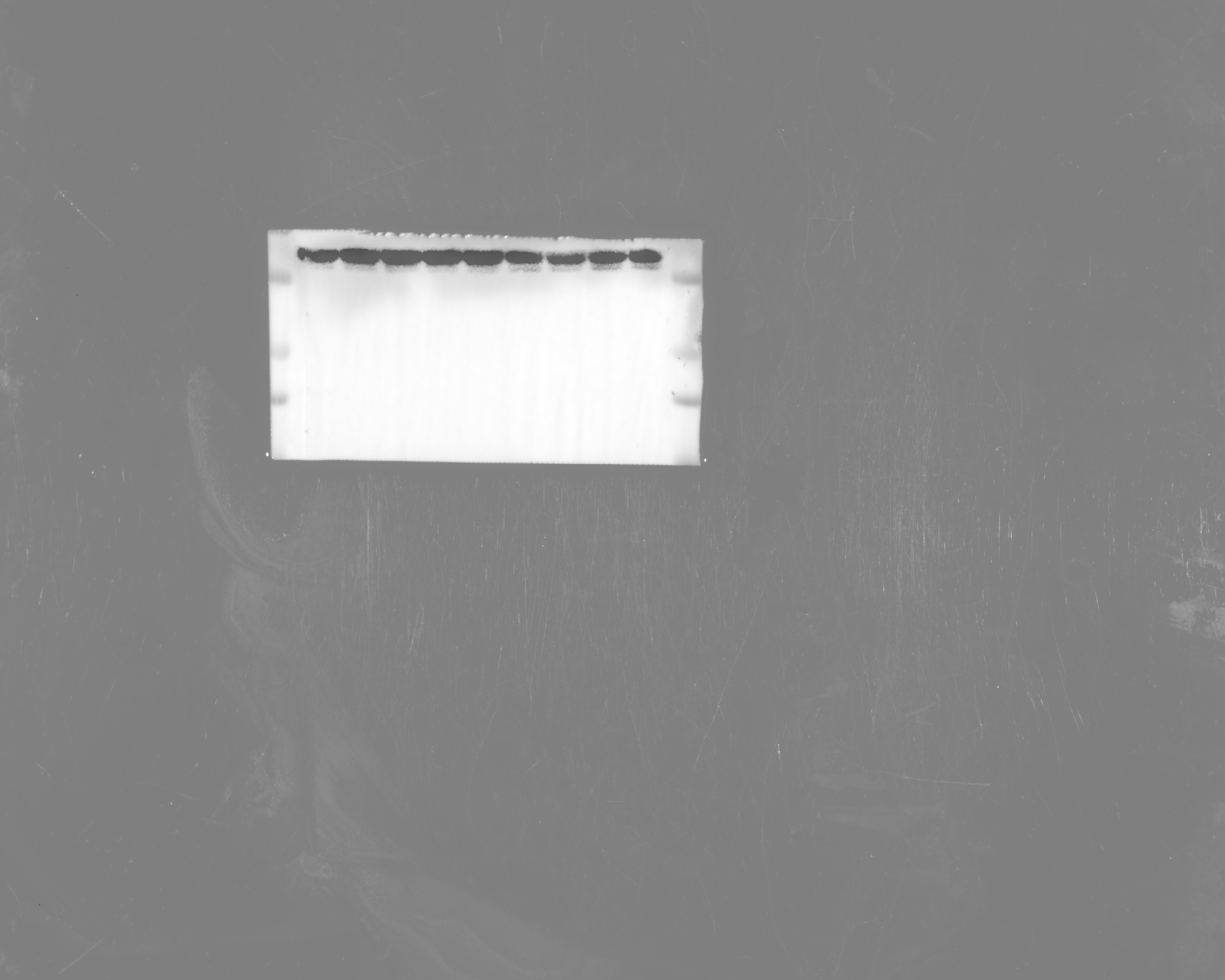

Supplement: Supplementary file 2 — Supporting File: advs75314‐sup‐0002‐RawData.zip. [file ADVS-13-e19337-s001.zip › F ovx tissue actin(Composite).tif]

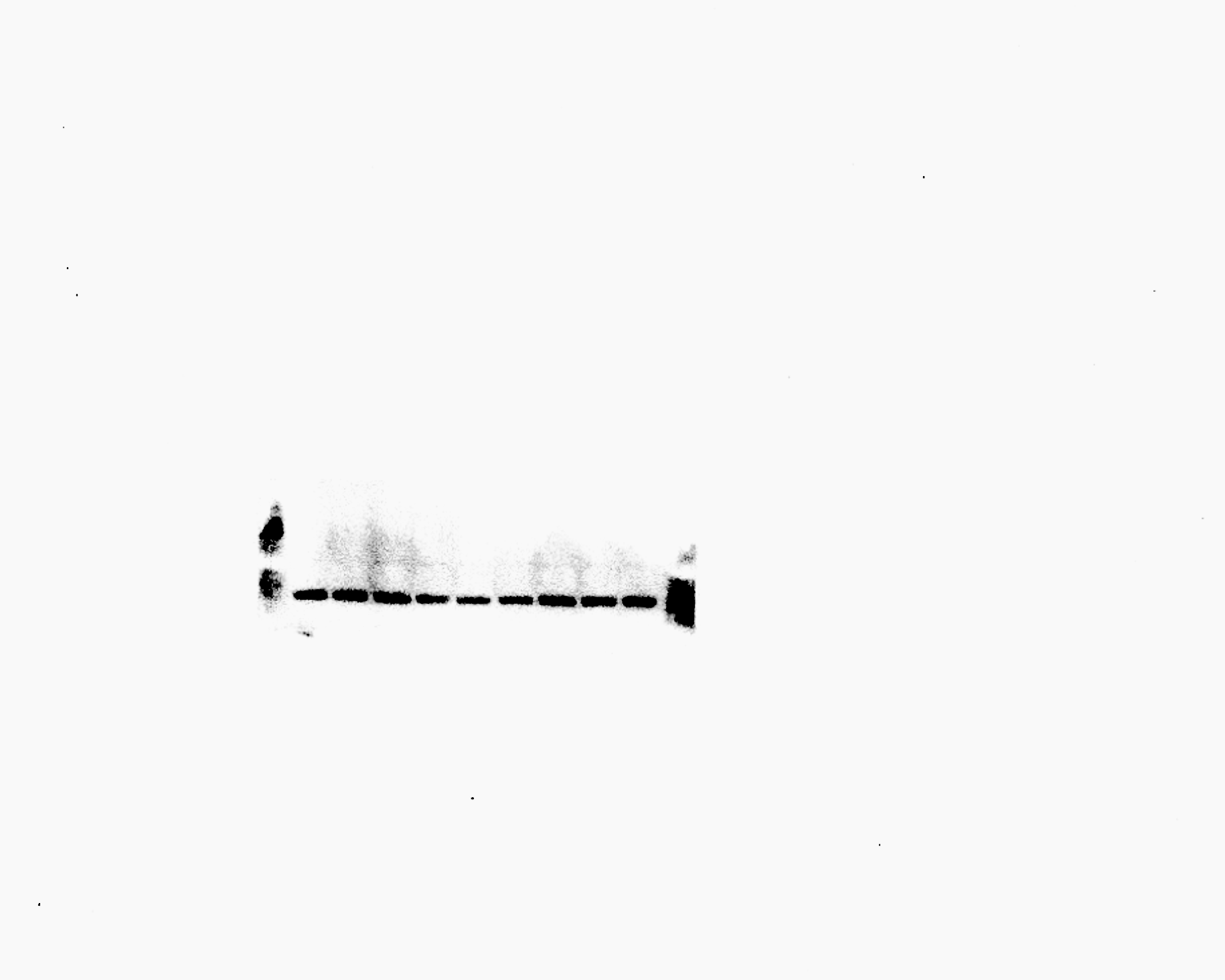

Supplement: Supplementary file 2 — Supporting File: advs75314‐sup‐0002‐RawData.zip. [file ADVS-13-e19337-s001.zip › F ovx tissue muc2(Chemiluminescence).tif]

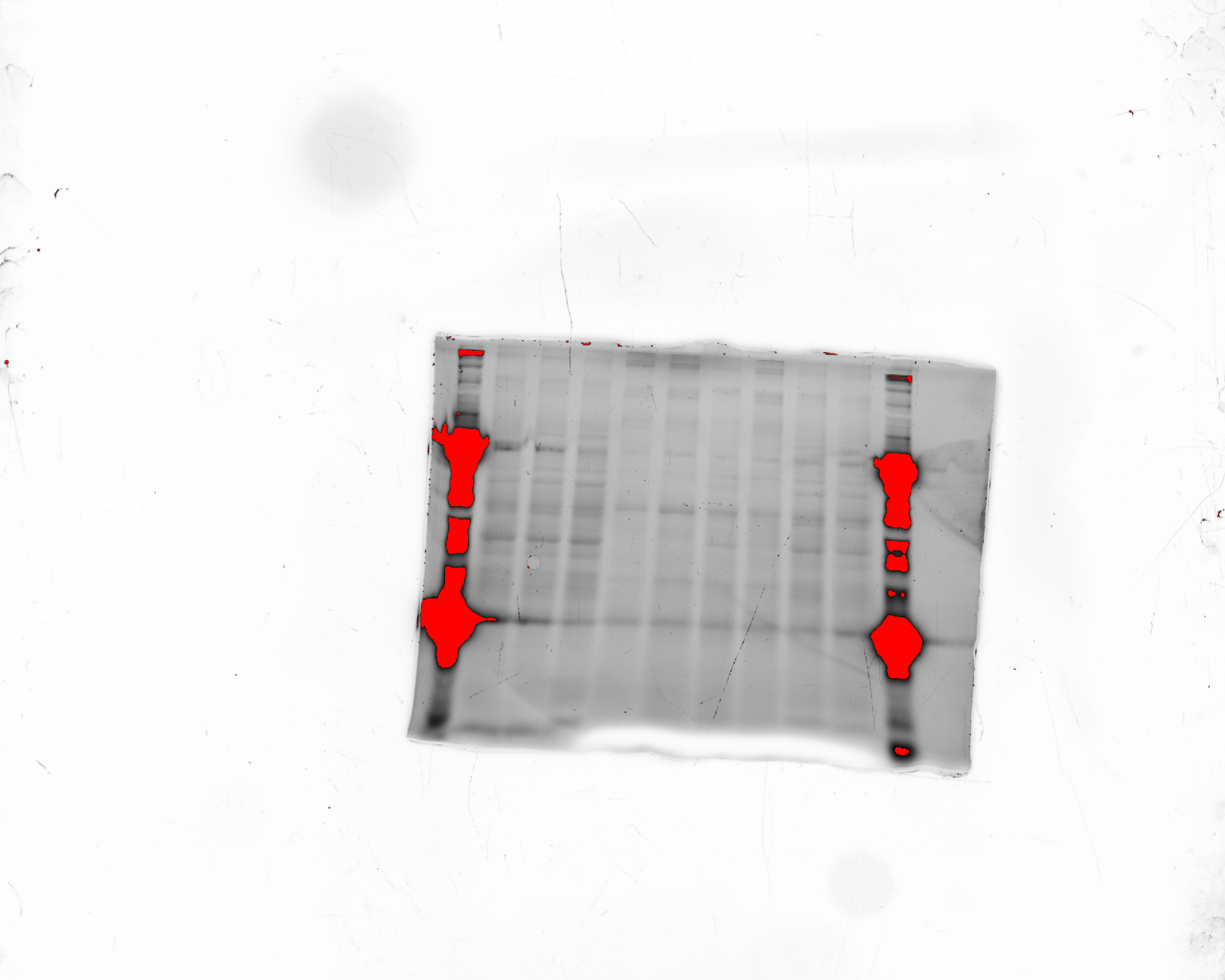

Supplement: Supplementary file 2 — Supporting File: advs75314‐sup‐0002‐RawData.zip. [file ADVS-13-e19337-s001.zip › F VOX jejunal fluid total protein(Stain Free Gel).tif]

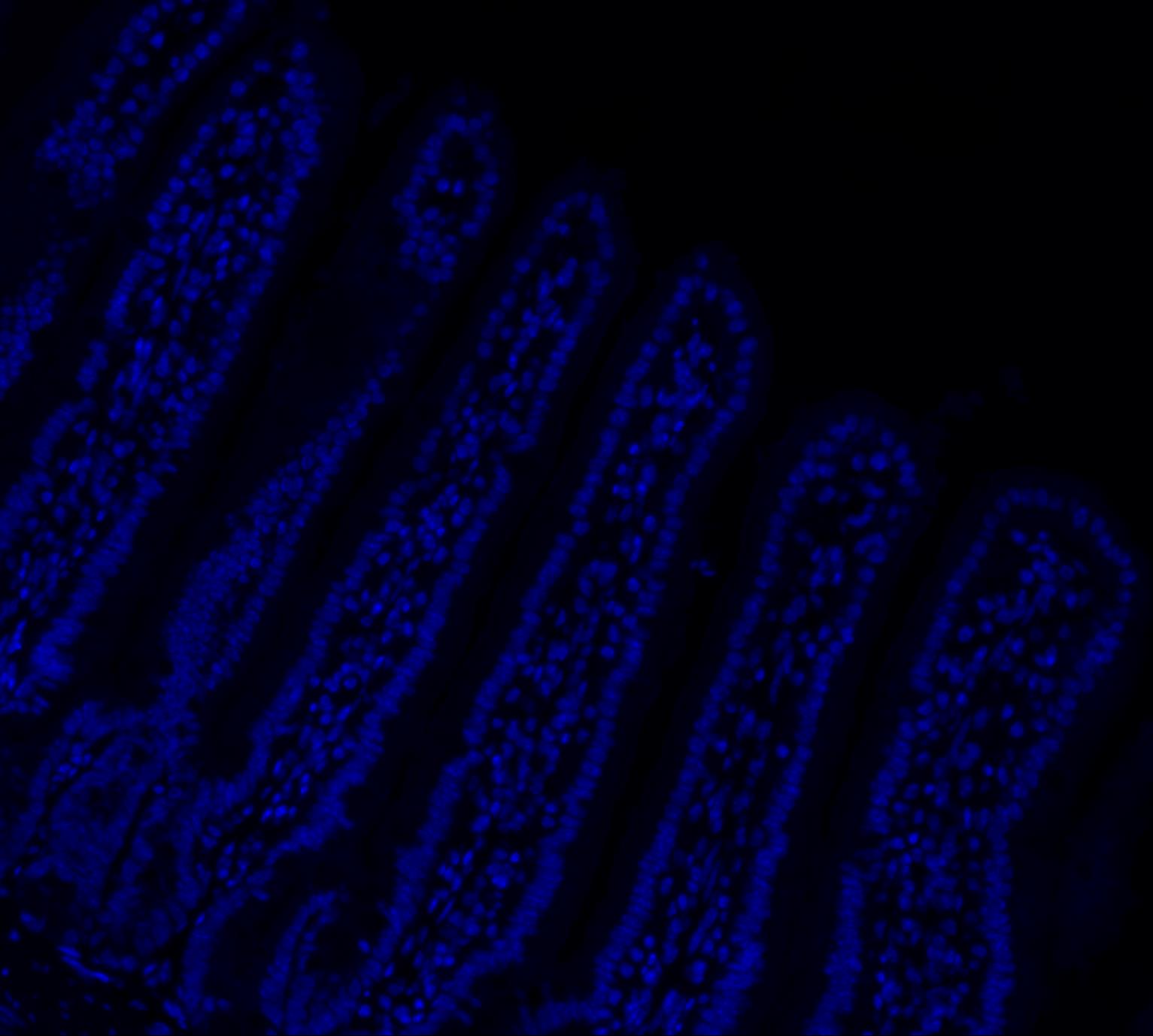

Supplement: Supplementary file 2 — Supporting File: advs75314‐sup‐0002‐RawData.zip. [file ADVS-13-e19337-s001.zip › Female DAPI.jpg]

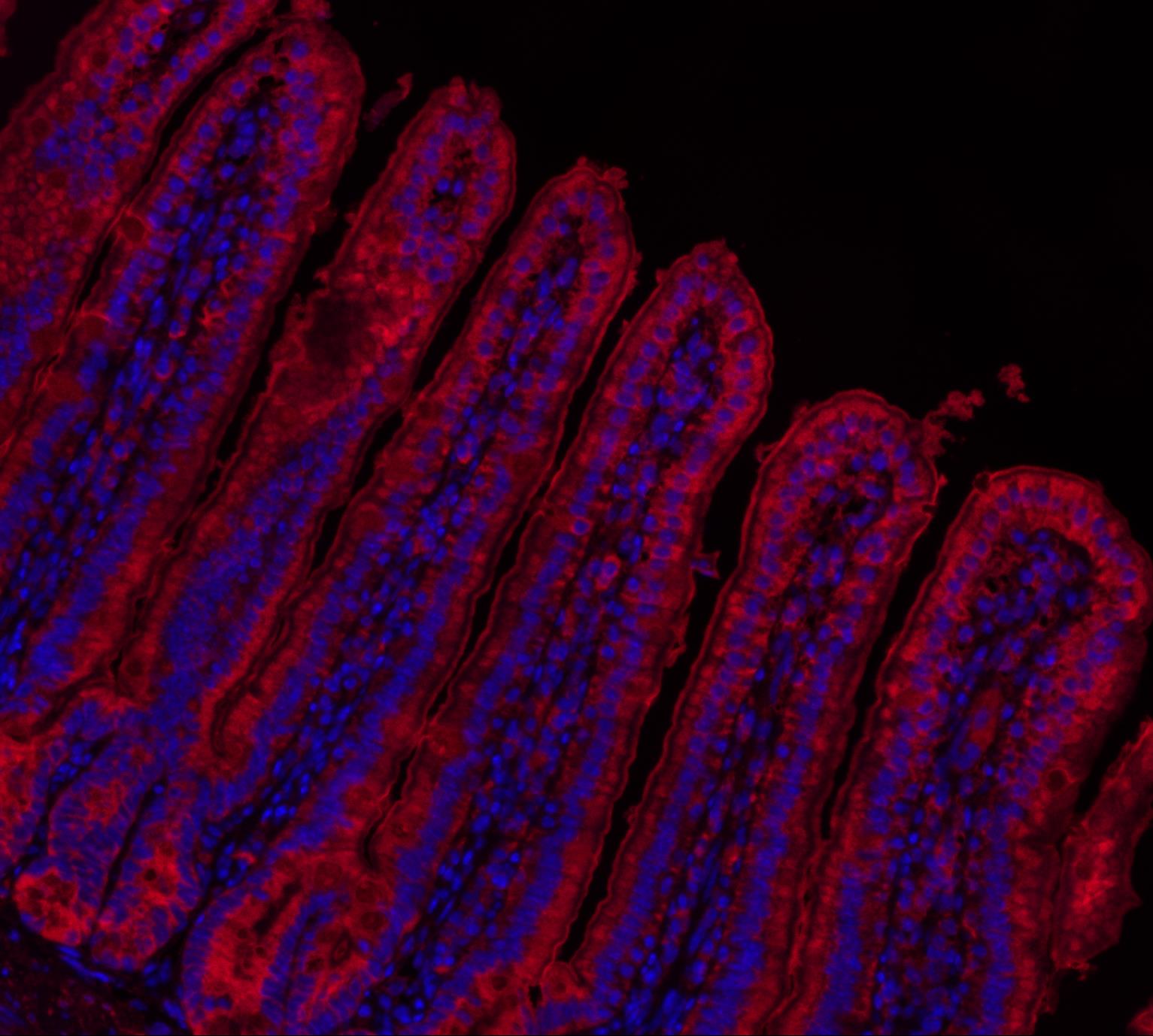

Supplement: Supplementary file 2 — Supporting File: advs75314‐sup‐0002‐RawData.zip. [file ADVS-13-e19337-s001.zip › Female Merged.jpg]

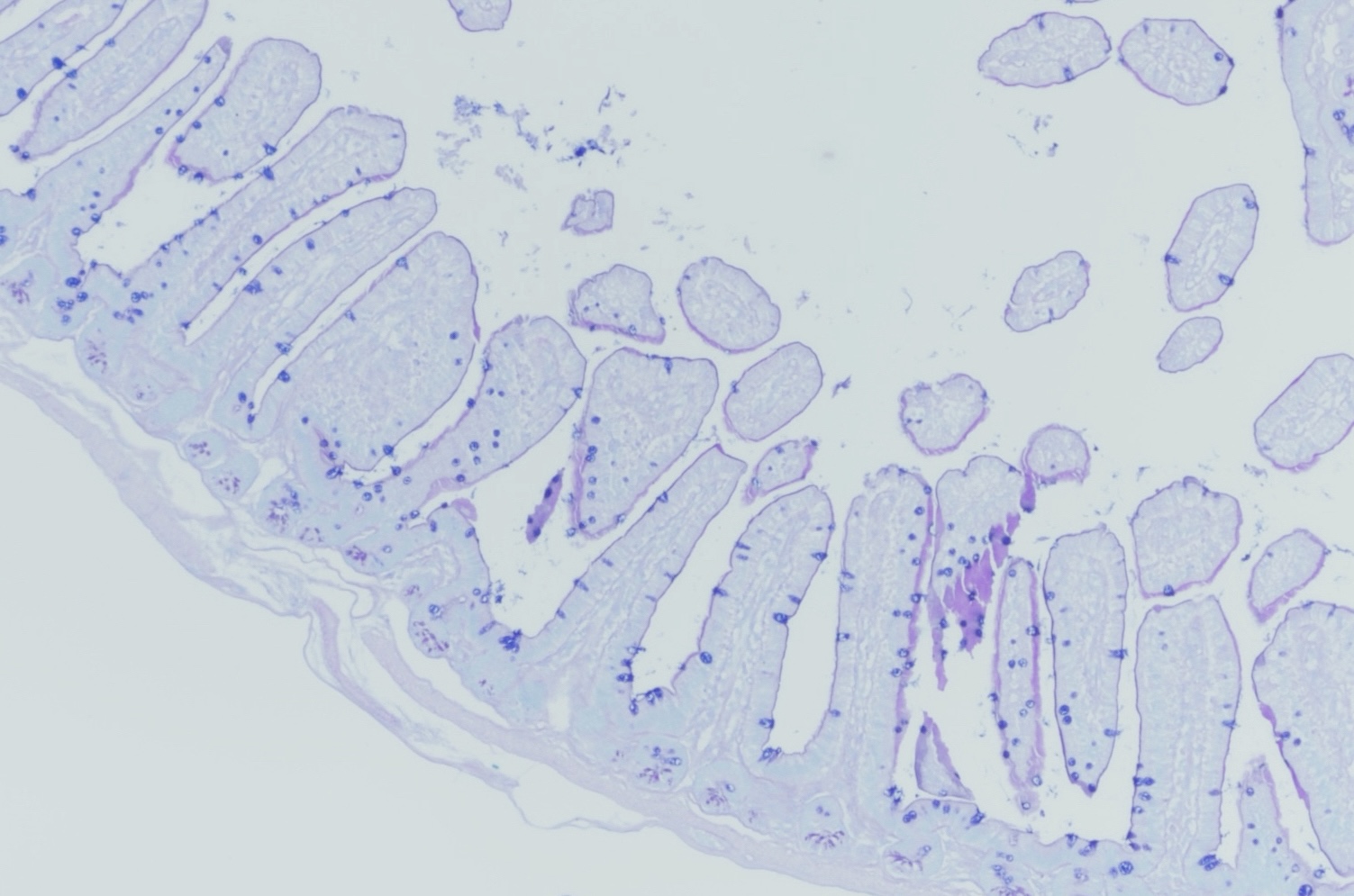

Supplement: Supplementary file 2 — Supporting File: advs75314‐sup‐0002‐RawData.zip. [file ADVS-13-e19337-s001.zip › Female mice.tif]

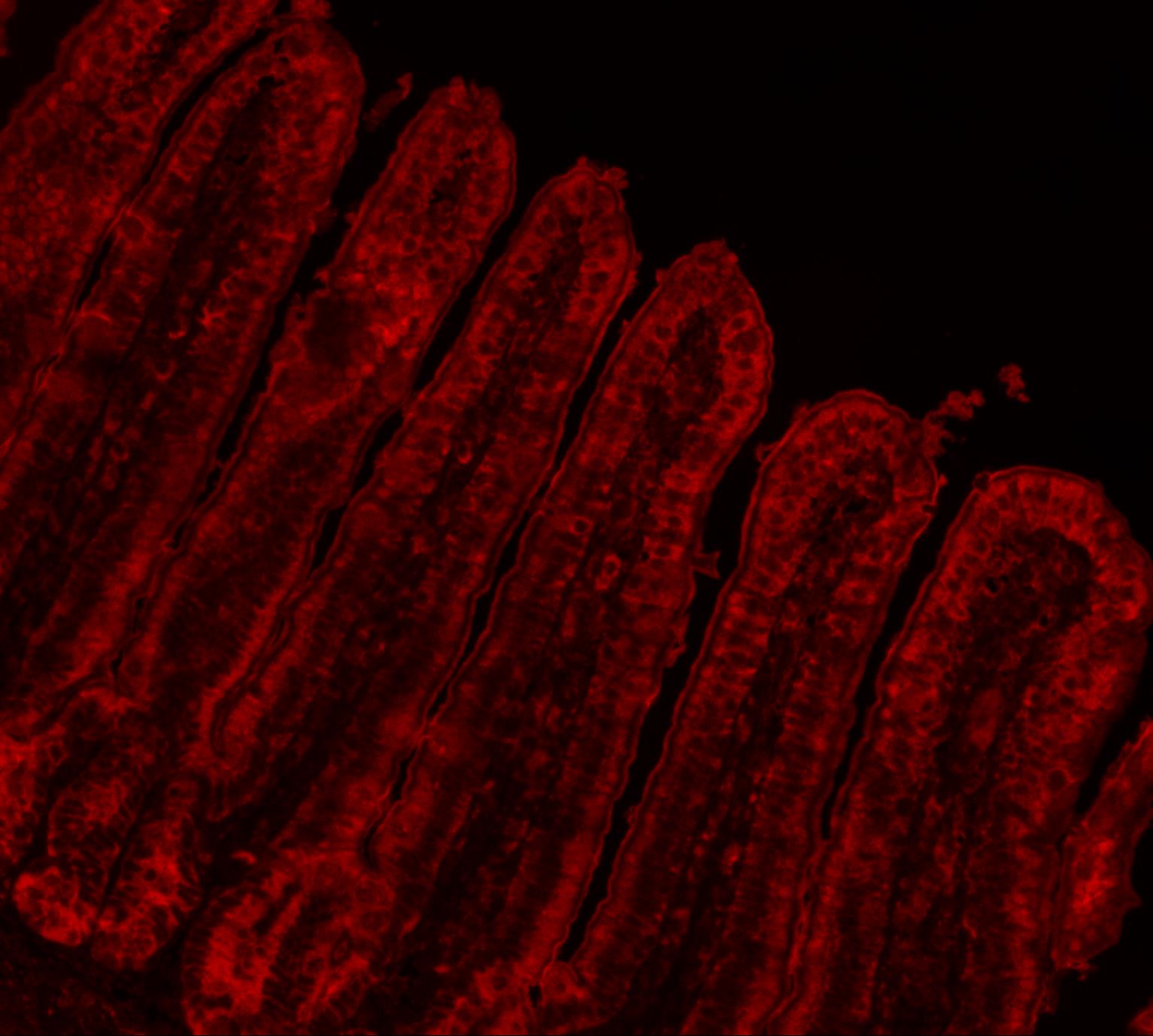

Supplement: Supplementary file 2 — Supporting File: advs75314‐sup‐0002‐RawData.zip. [file ADVS-13-e19337-s001.zip › Female MUC2.jpg]

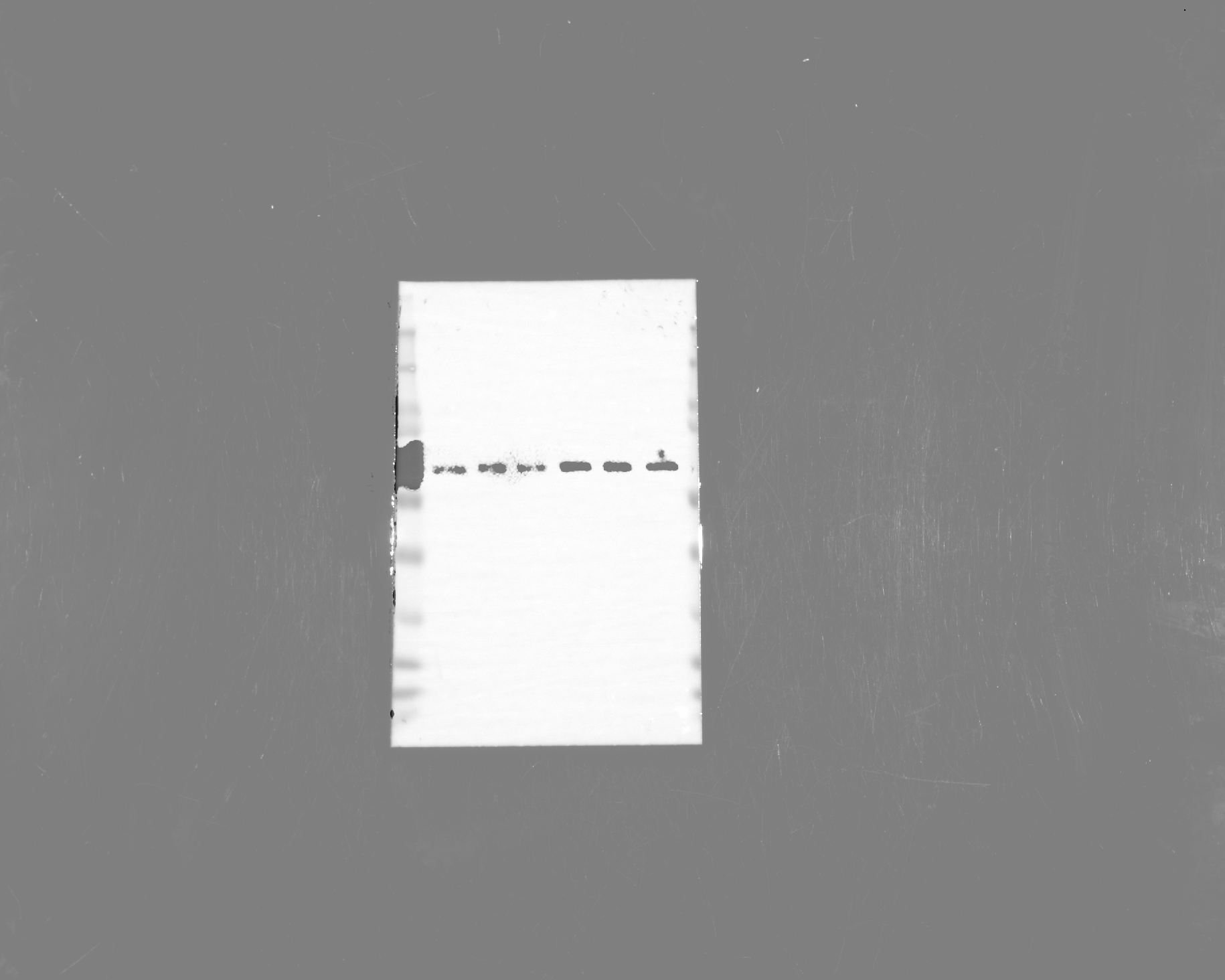

Supplement: Supplementary file 2 — Supporting File: advs75314‐sup‐0002‐RawData.zip. [file ADVS-13-e19337-s001.zip › HFD+STZ mice jejunal fluid muc2(Composite).tif]

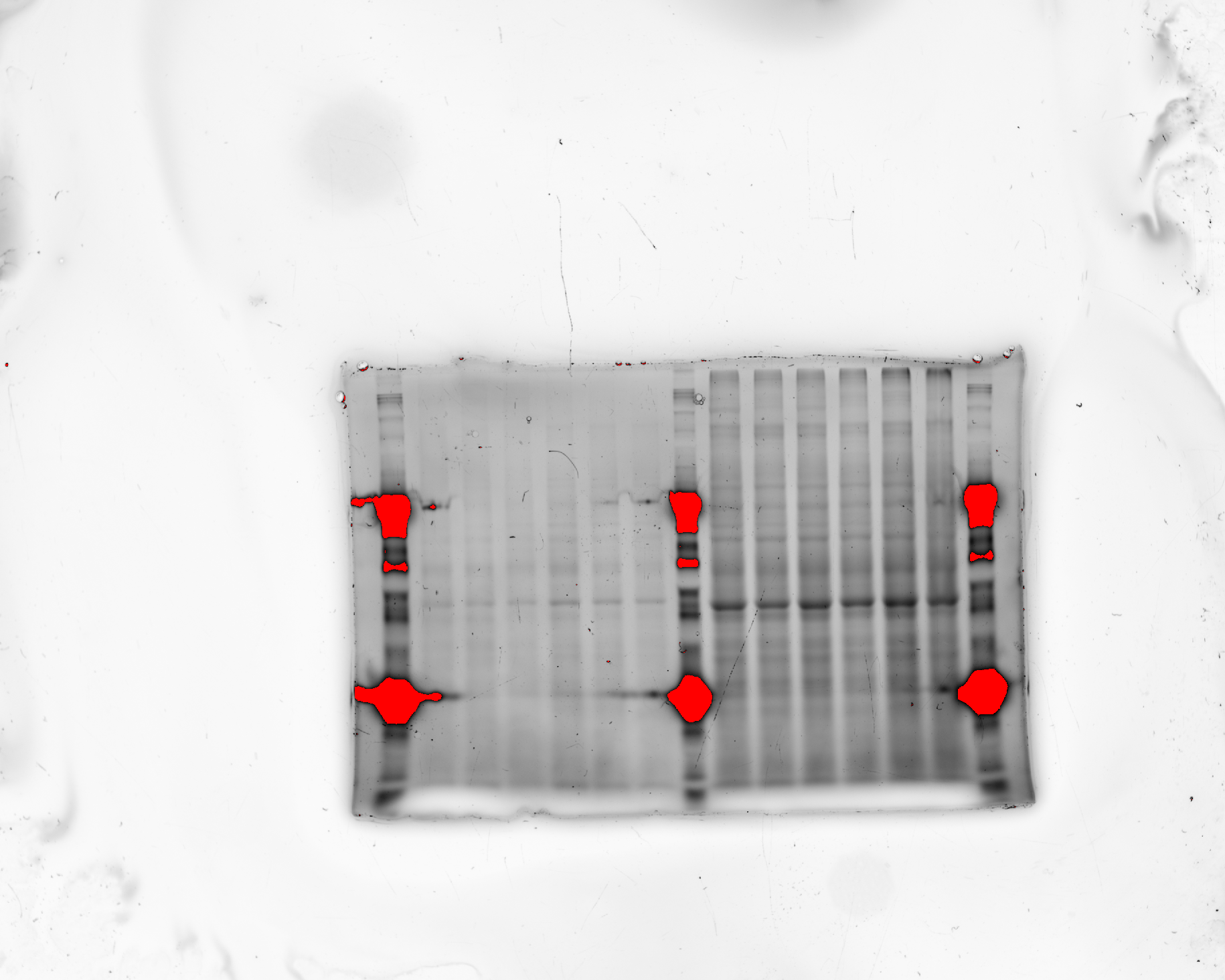

Supplement: Supplementary file 2 — Supporting File: advs75314‐sup‐0002‐RawData.zip. [file ADVS-13-e19337-s001.zip › HFD+STZ mice jejunal fluid total protein(Stain Free Gel).tif]

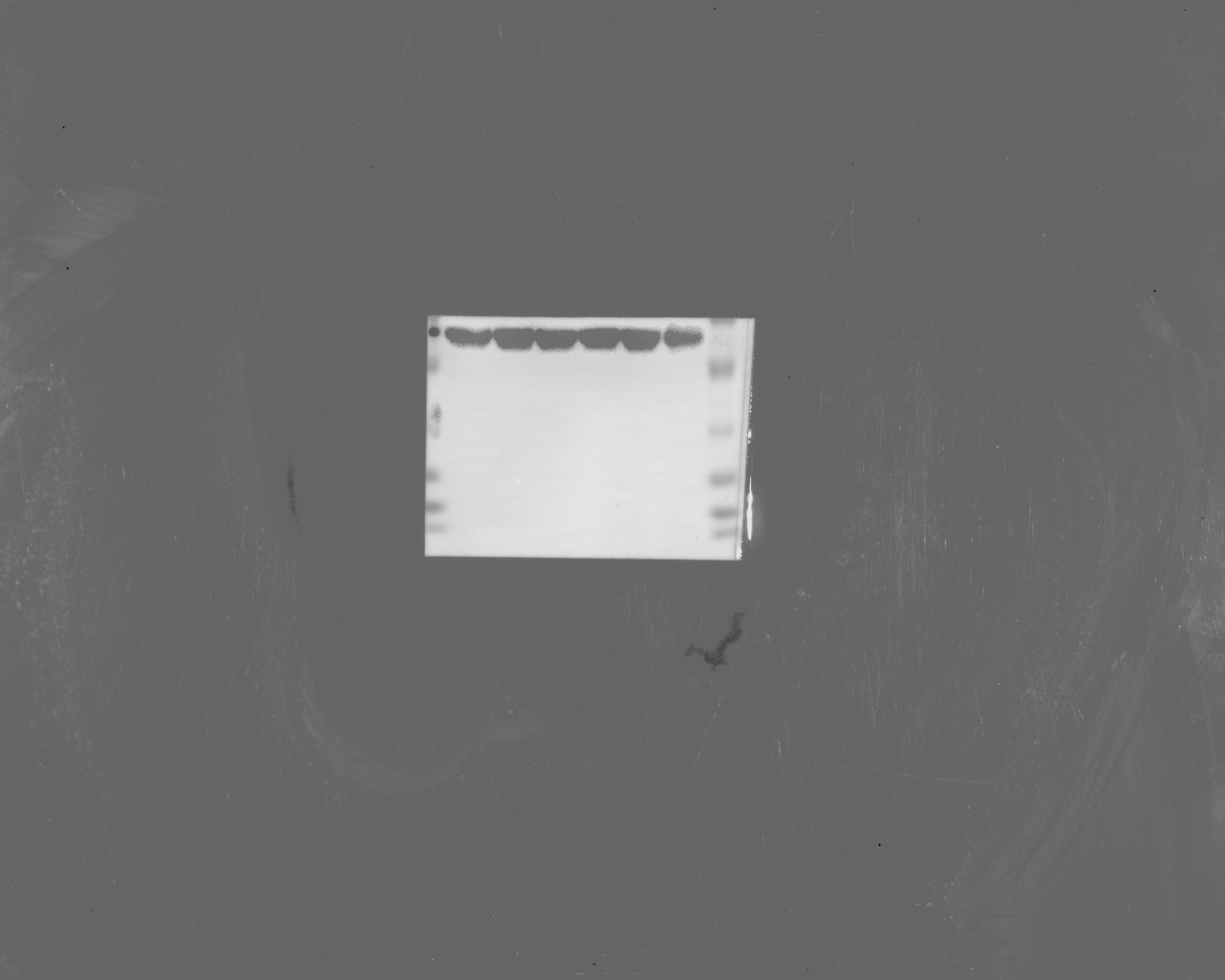

Supplement: Supplementary file 2 — Supporting File: advs75314‐sup‐0002‐RawData.zip. [file ADVS-13-e19337-s001.zip › HFD+STZ mice tissue actin(Composite).tif]

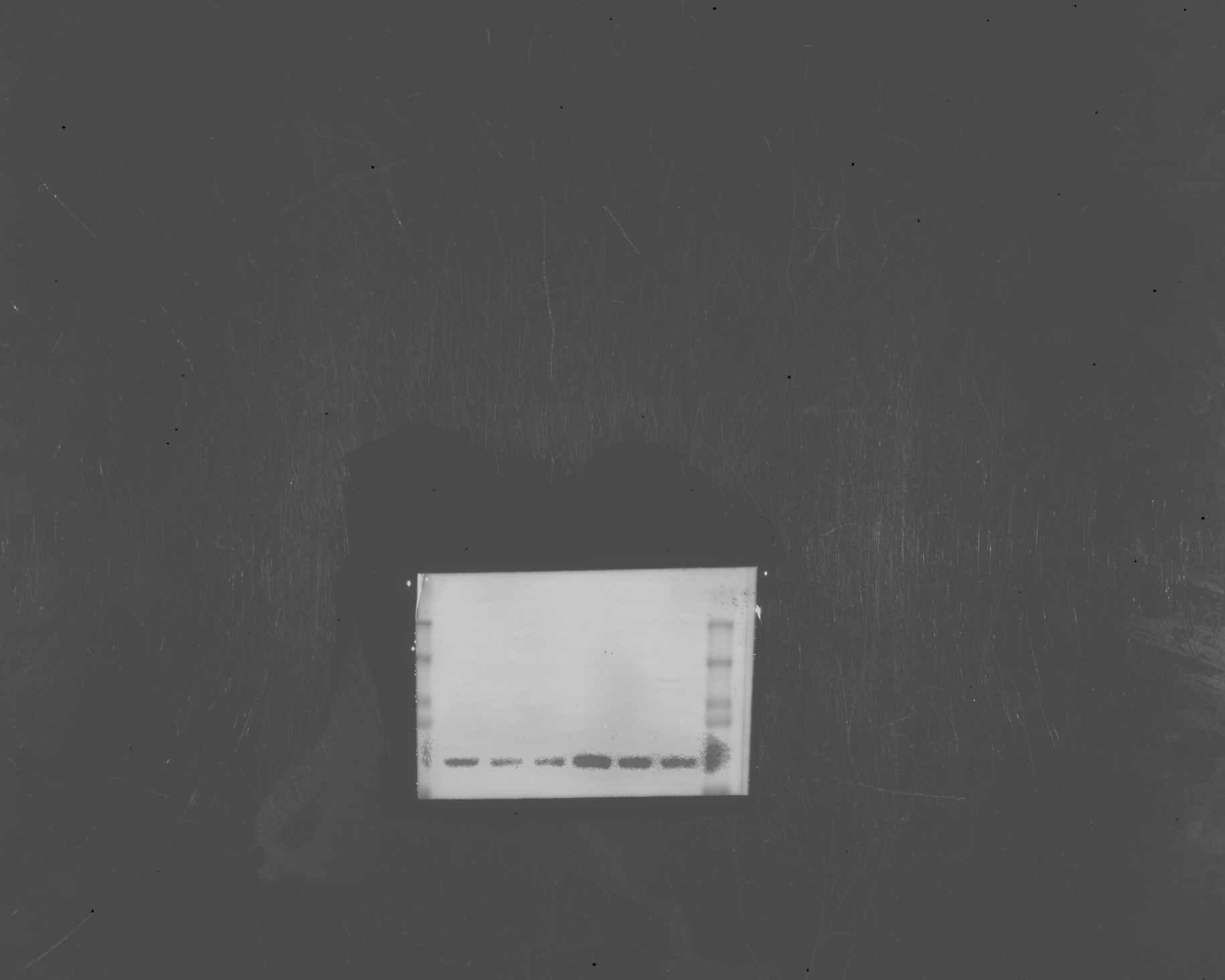

Supplement: Supplementary file 2 — Supporting File: advs75314‐sup‐0002‐RawData.zip. [file ADVS-13-e19337-s001.zip › HFD+STZ mice tissue muc2(Composite).tif]

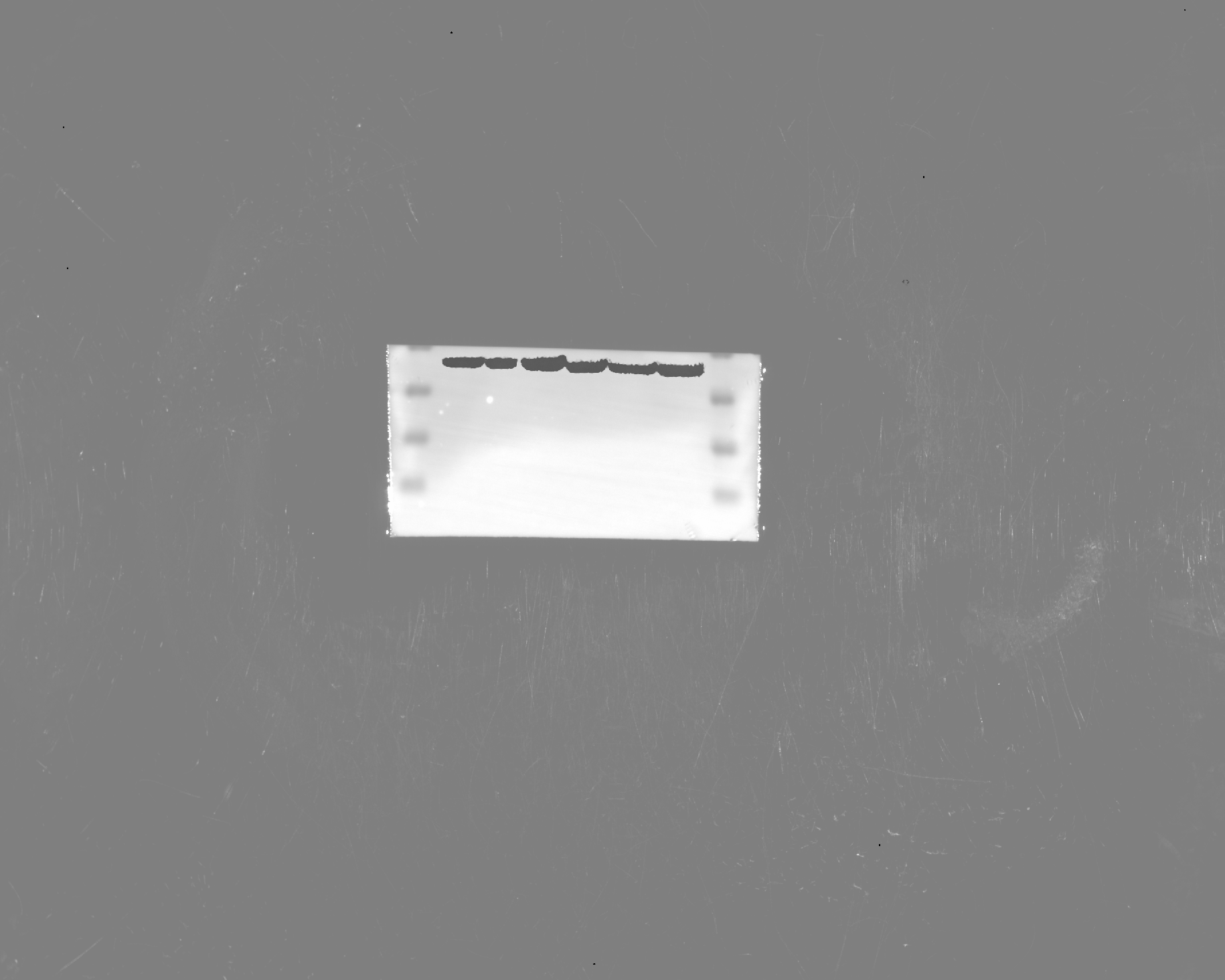

Supplement: Supplementary file 2 — Supporting File: advs75314‐sup‐0002‐RawData.zip. [file ADVS-13-e19337-s001.zip › HT-29 cell actin(Composite).tif]

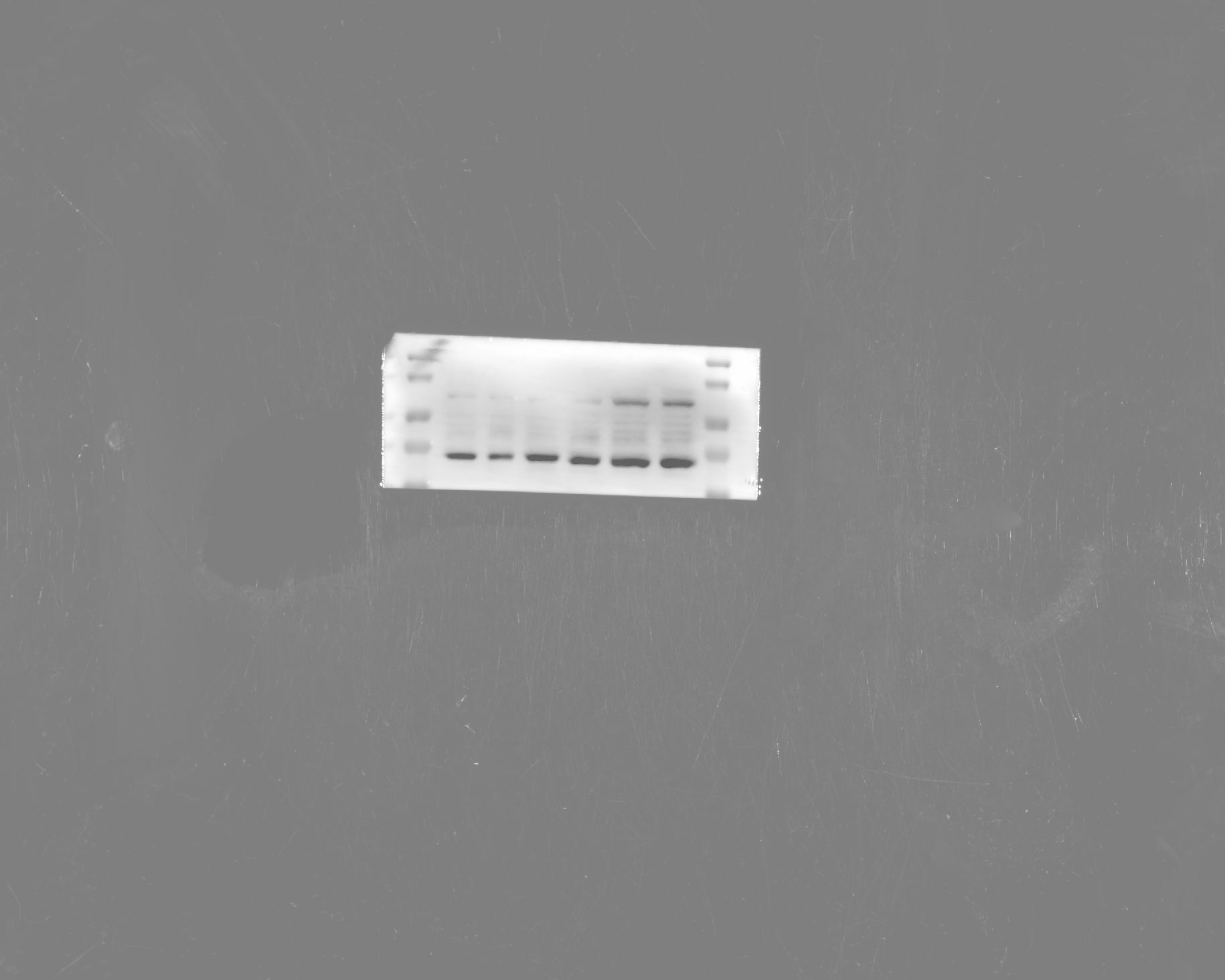

Supplement: Supplementary file 2 — Supporting File: advs75314‐sup‐0002‐RawData.zip. [file ADVS-13-e19337-s001.zip › HT-29 cell Muc2(Composite).tif]

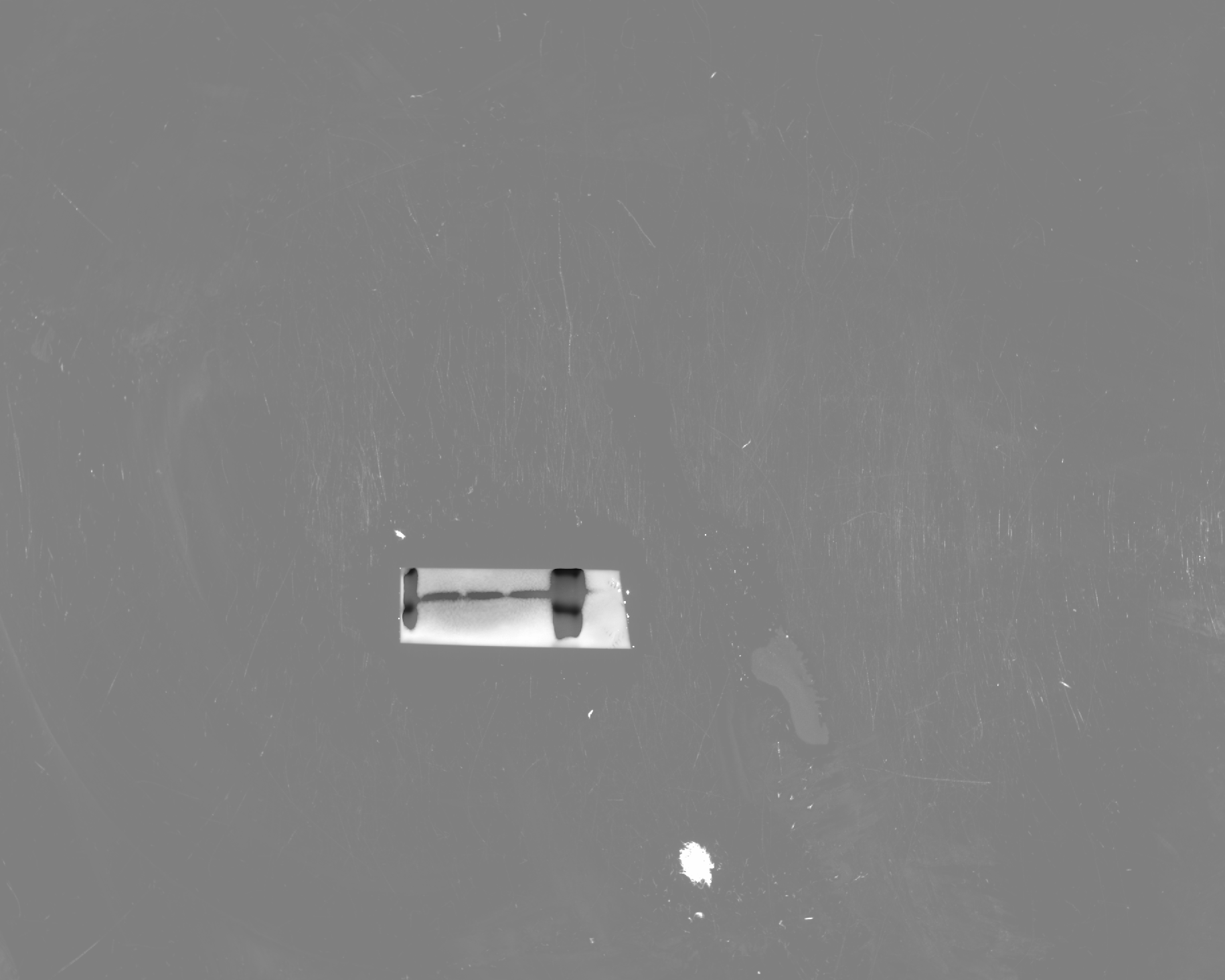

Supplement: Supplementary file 2 — Supporting File: advs75314‐sup‐0002‐RawData.zip. [file ADVS-13-e19337-s001.zip › ko actin(Composite).tif]

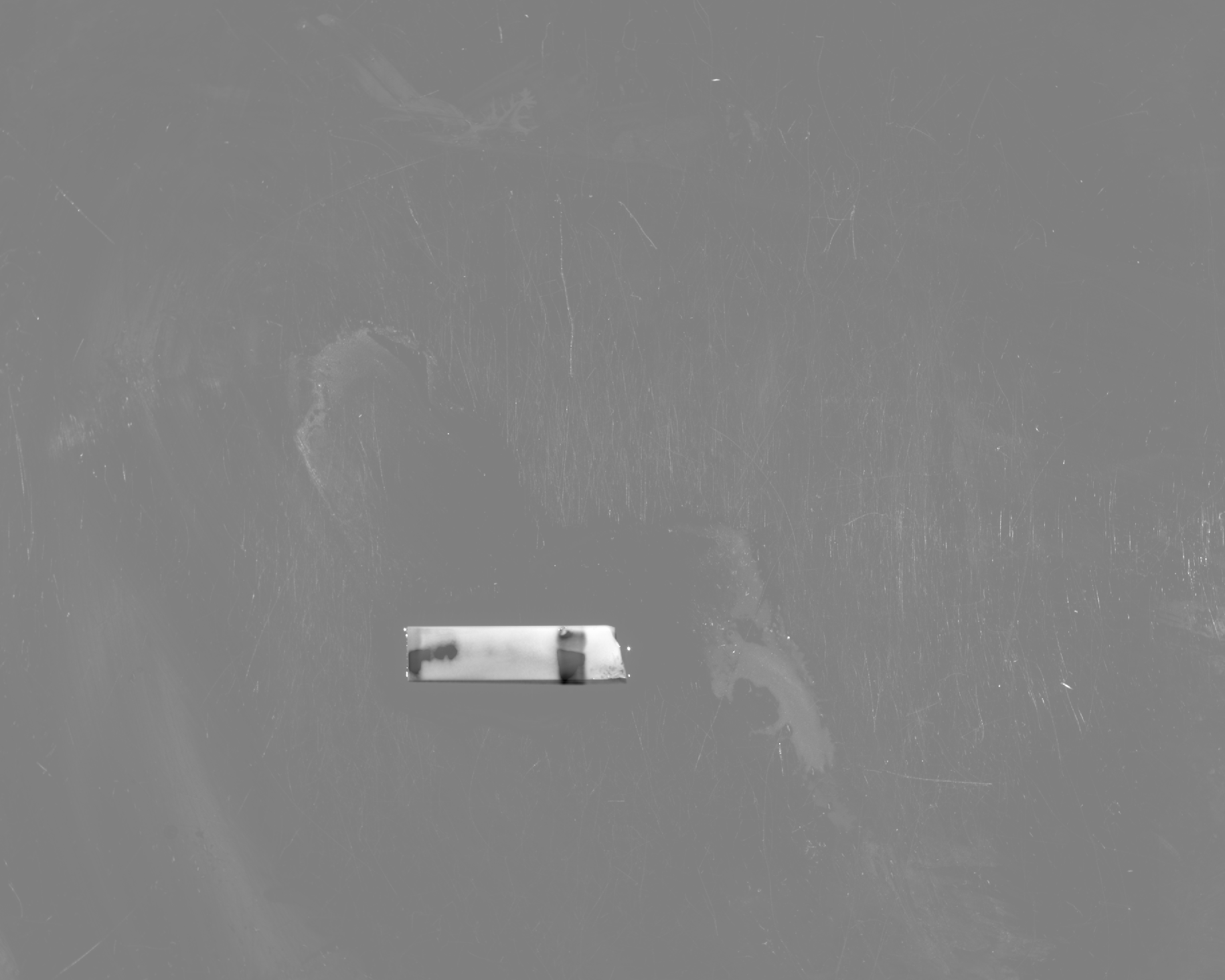

Supplement: Supplementary file 2 — Supporting File: advs75314‐sup‐0002‐RawData.zip. [file ADVS-13-e19337-s001.zip › ko muc2 cells(Composite).tif]

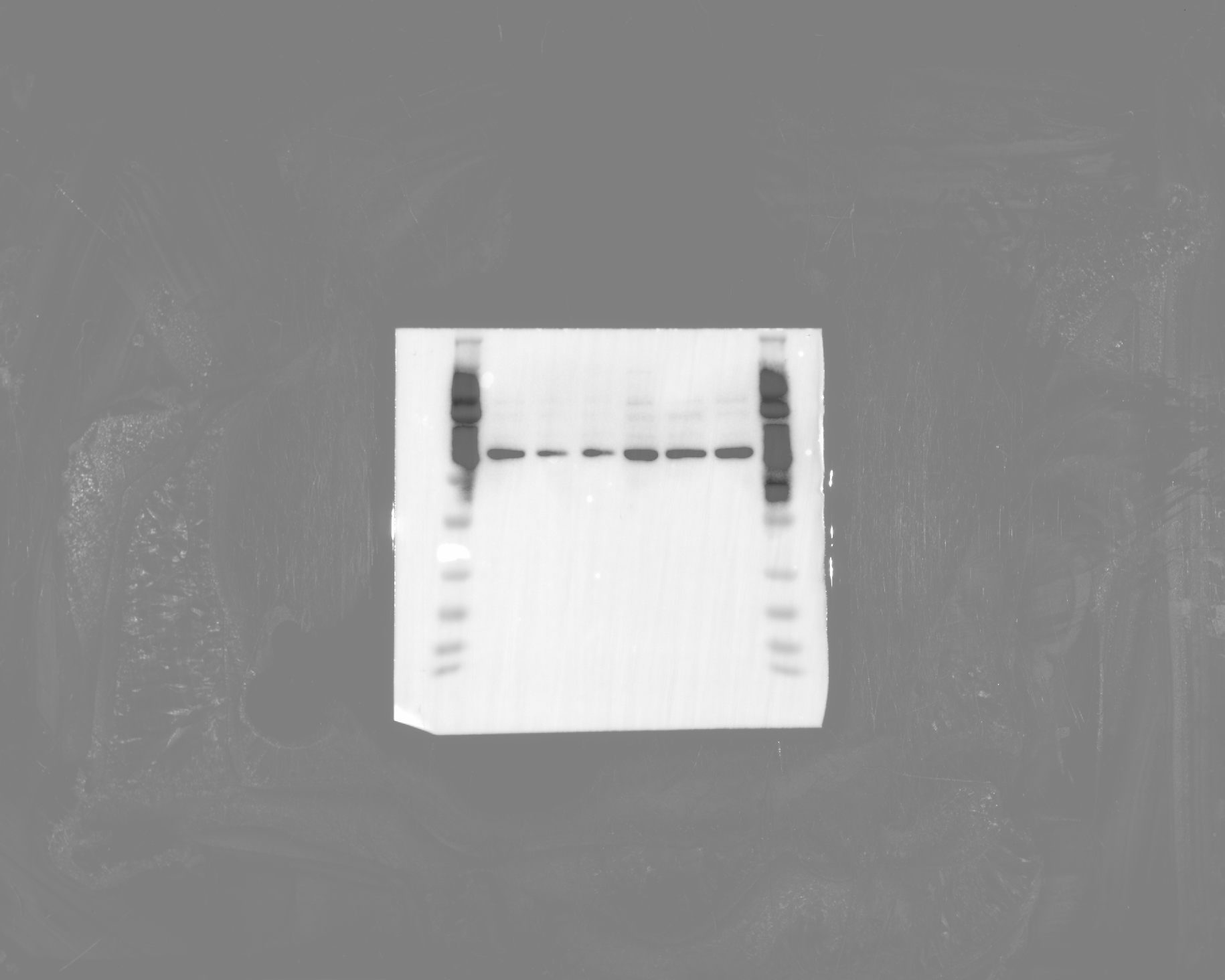

Supplement: Supplementary file 2 — Supporting File: advs75314‐sup‐0002‐RawData.zip. [file ADVS-13-e19337-s001.zip › M+E2 jejunal fluid muc2(Composite).tif]

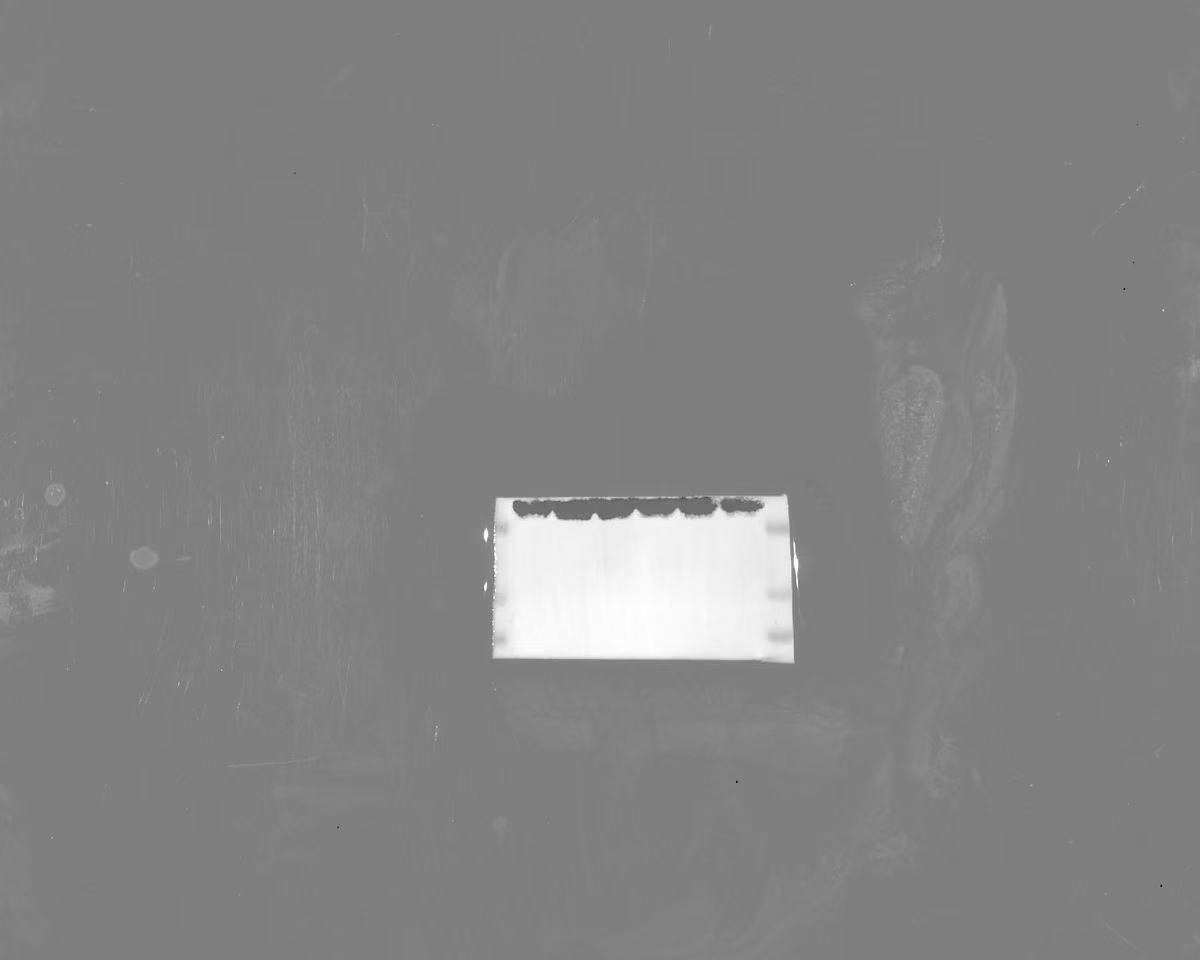

Supplement: Supplementary file 2 — Supporting File: advs75314‐sup‐0002‐RawData.zip. [file ADVS-13-e19337-s001.zip › M+E2 tissue actin(Composite).tif]

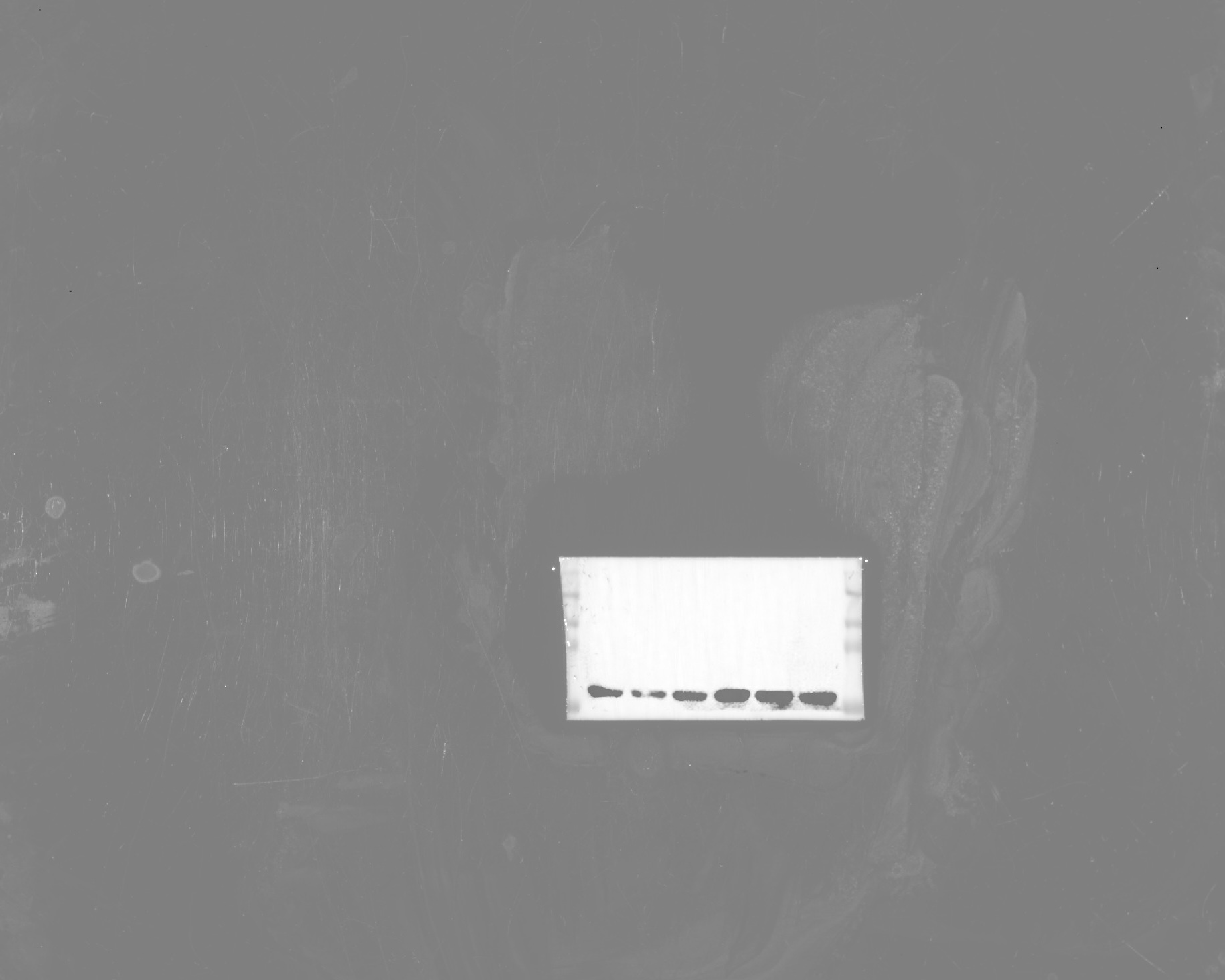

Supplement: Supplementary file 2 — Supporting File: advs75314‐sup‐0002‐RawData.zip. [file ADVS-13-e19337-s001.zip › M+E2 tissue muc2(Composite).tif]

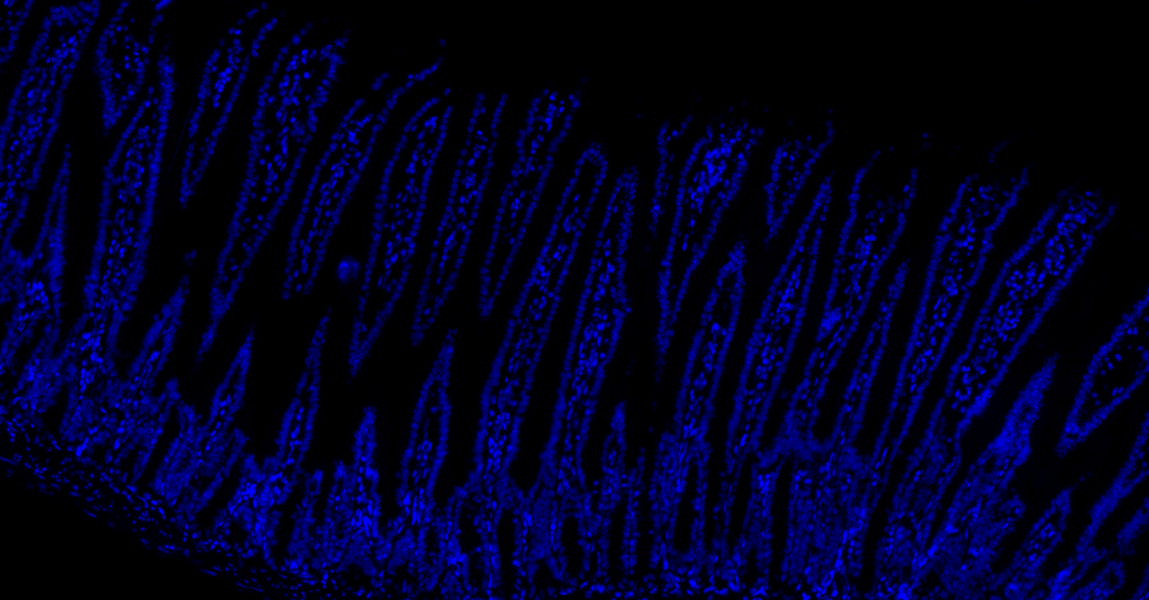

Supplement: Supplementary file 2 — Supporting File: advs75314‐sup‐0002‐RawData.zip. [file ADVS-13-e19337-s001.zip › M1+E2 H DAPI.tif]

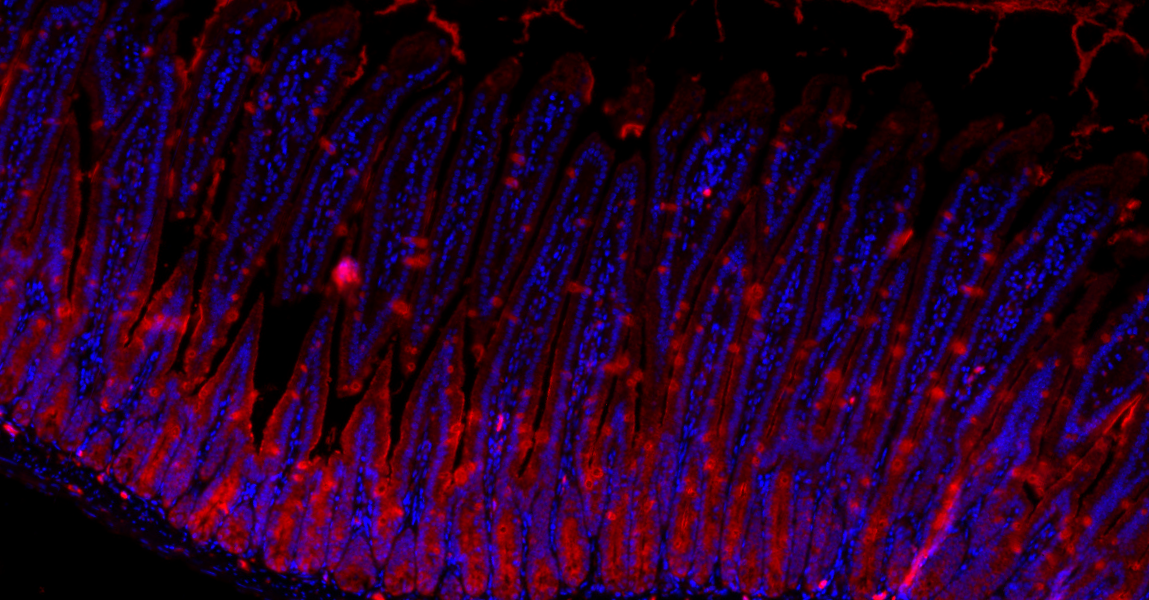

Supplement: Supplementary file 2 — Supporting File: advs75314‐sup‐0002‐RawData.zip. [file ADVS-13-e19337-s001.zip › M1+E2 H Merged.tif]

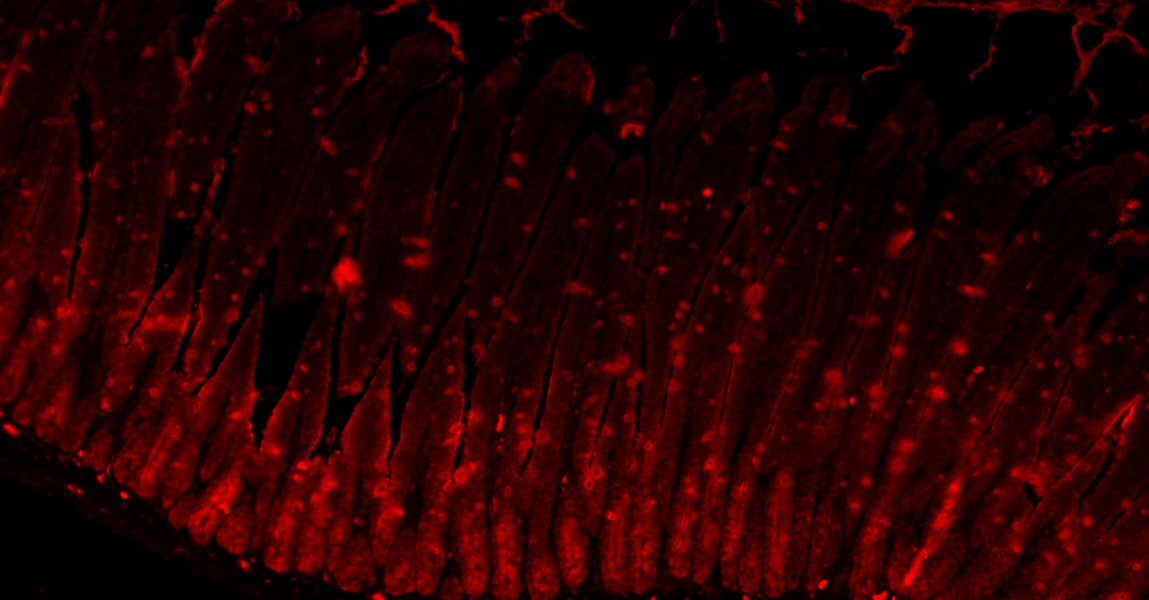

Supplement: Supplementary file 2 — Supporting File: advs75314‐sup‐0002‐RawData.zip. [file ADVS-13-e19337-s001.zip › M1+E2 H MUC2.tif]

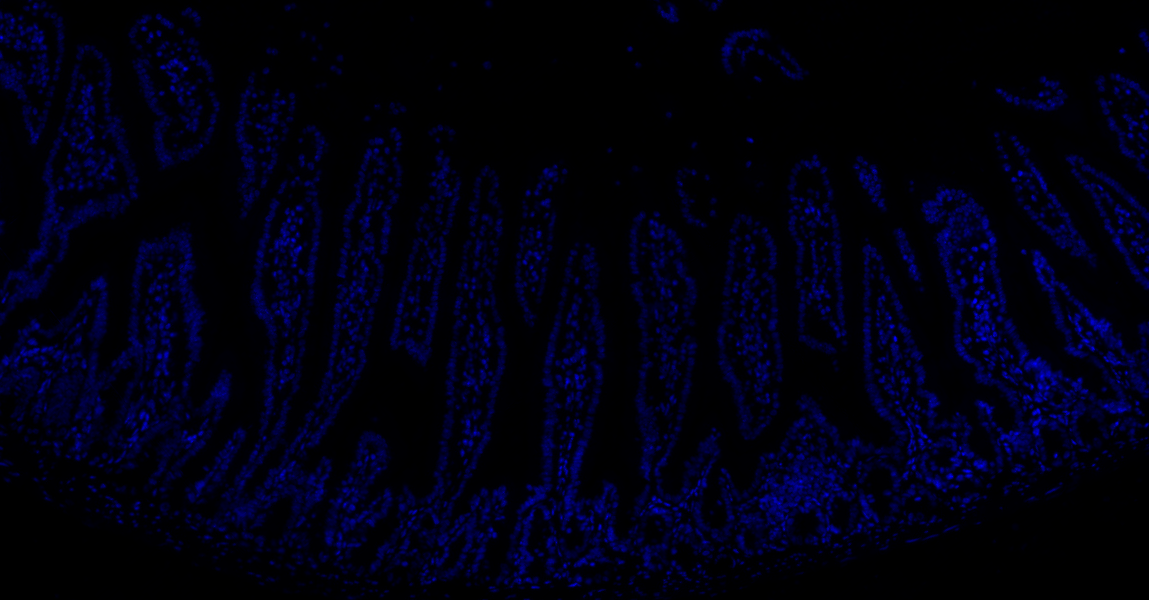

Supplement: Supplementary file 2 — Supporting File: advs75314‐sup‐0002‐RawData.zip. [file ADVS-13-e19337-s001.zip › M2+E2 L DAPI.tif]

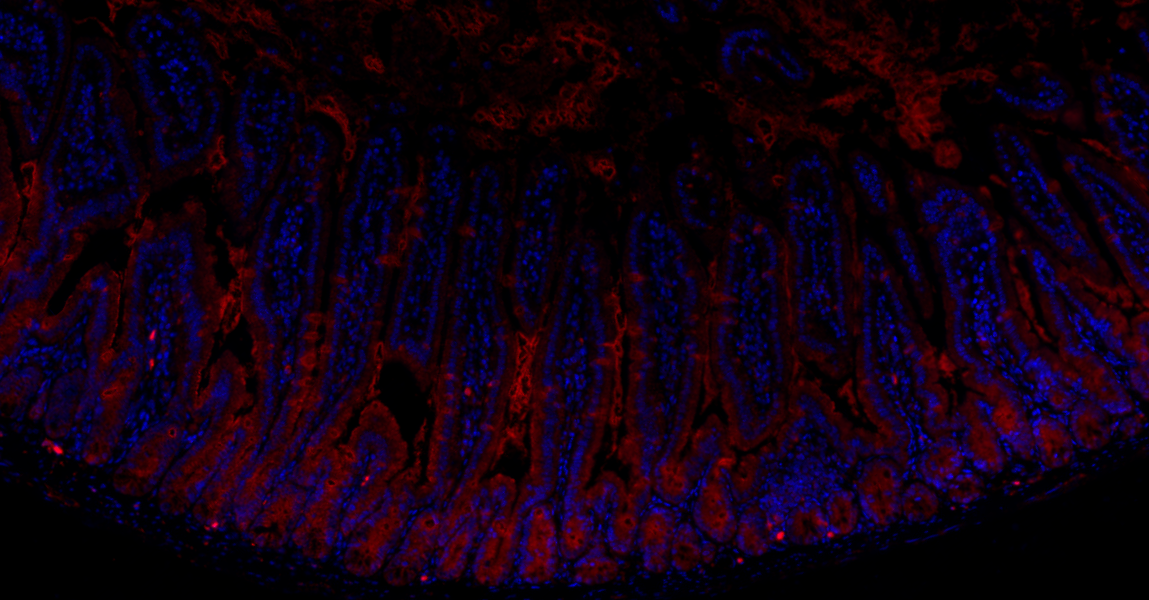

Supplement: Supplementary file 2 — Supporting File: advs75314‐sup‐0002‐RawData.zip. [file ADVS-13-e19337-s001.zip › M2+E2 L Merged.tif]

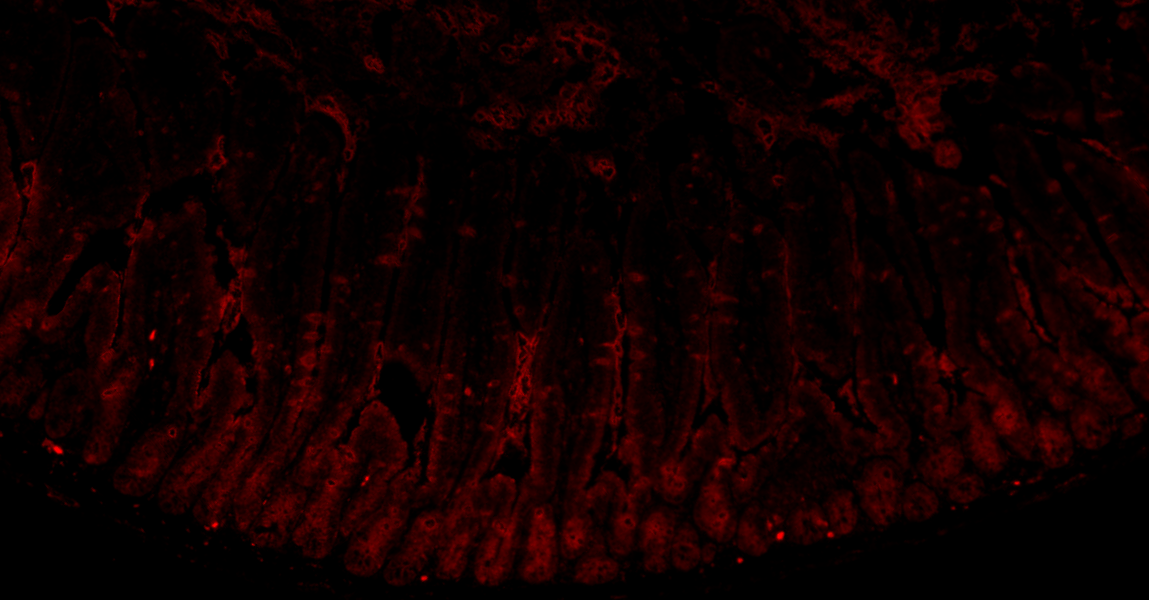

Supplement: Supplementary file 2 — Supporting File: advs75314‐sup‐0002‐RawData.zip. [file ADVS-13-e19337-s001.zip › M2+E2 L MUC2.tif]

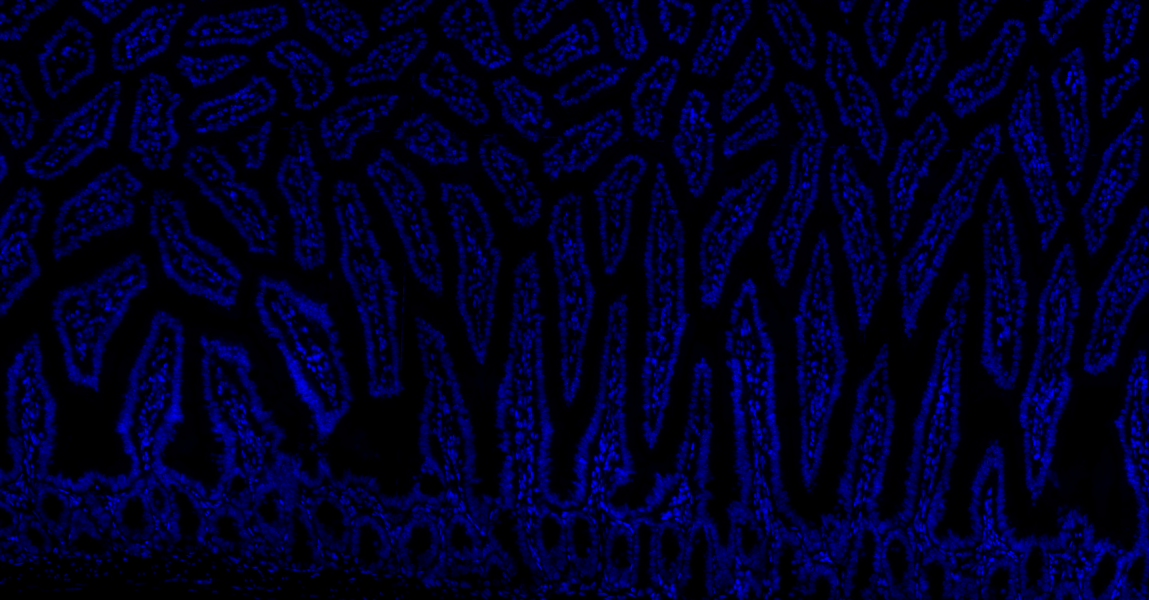

Supplement: Supplementary file 2 — Supporting File: advs75314‐sup‐0002‐RawData.zip. [file ADVS-13-e19337-s001.zip › M3+E2 M DAPI.tif]

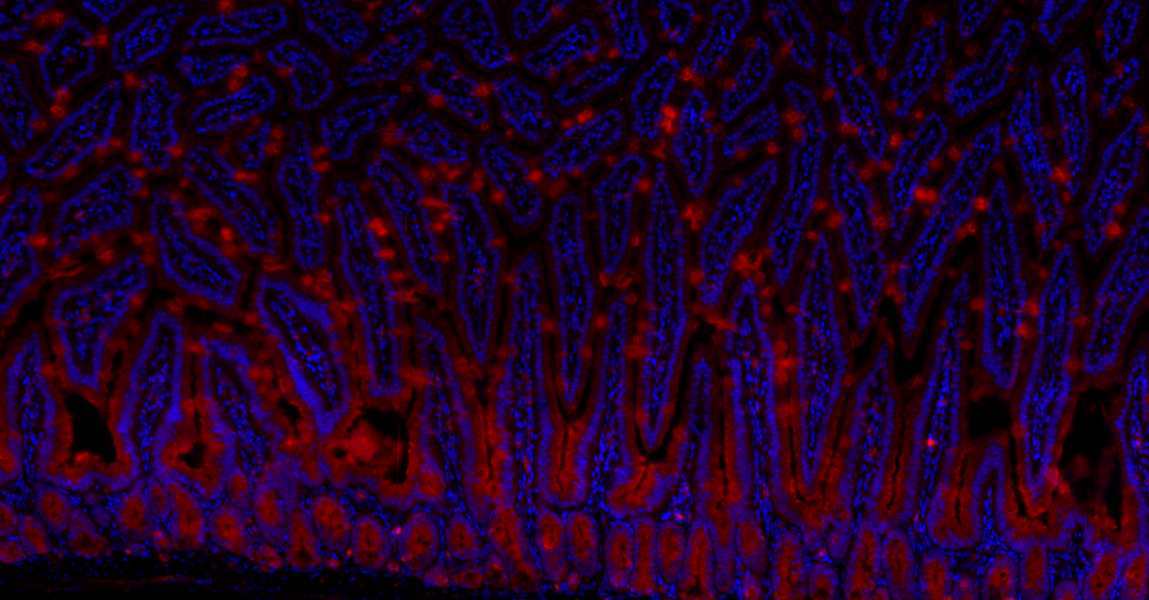

Supplement: Supplementary file 2 — Supporting File: advs75314‐sup‐0002‐RawData.zip. [file ADVS-13-e19337-s001.zip › M3+E2 M Merged.tif]

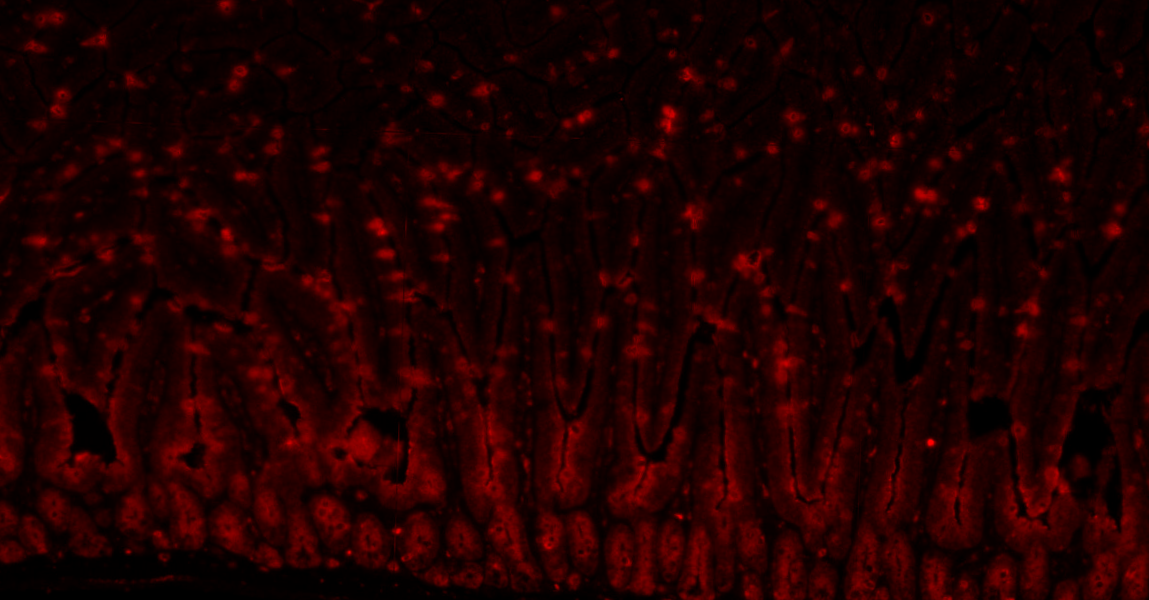

Supplement: Supplementary file 2 — Supporting File: advs75314‐sup‐0002‐RawData.zip. [file ADVS-13-e19337-s001.zip › M3+E2 M MUC2.tif]

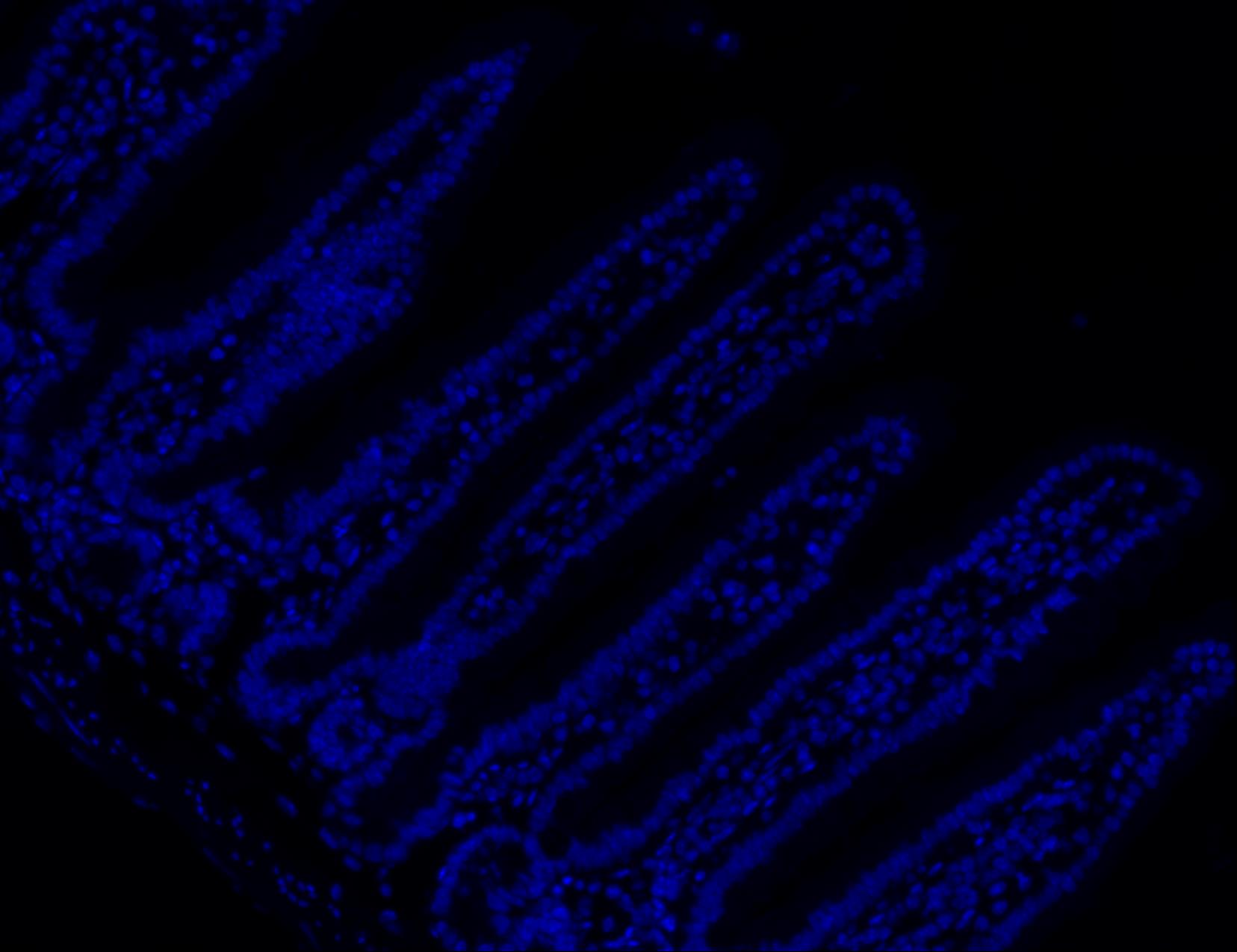

Supplement: Supplementary file 2 — Supporting File: advs75314‐sup‐0002‐RawData.zip. [file ADVS-13-e19337-s001.zip › Male DAPI.jpg]

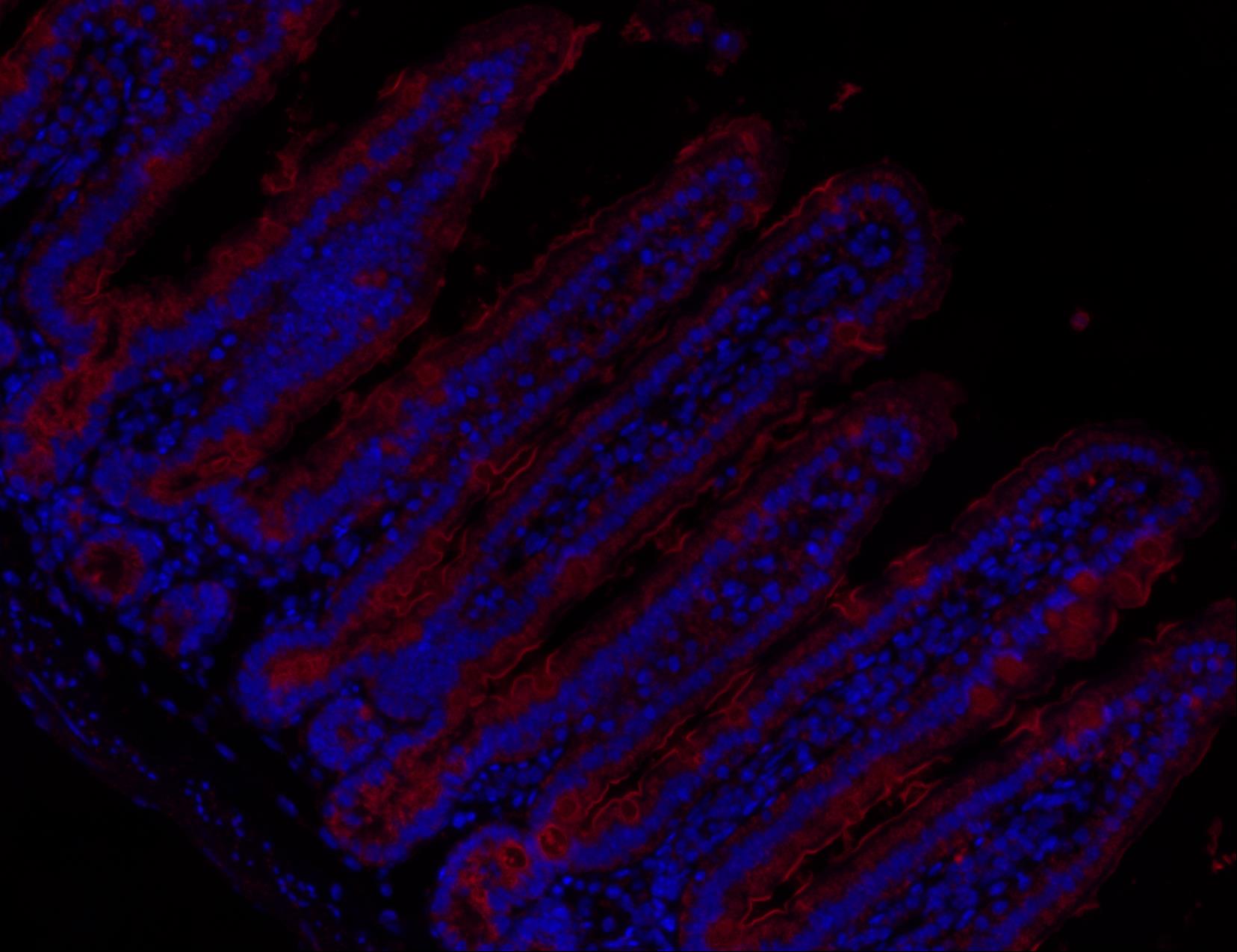

Supplement: Supplementary file 2 — Supporting File: advs75314‐sup‐0002‐RawData.zip. [file ADVS-13-e19337-s001.zip › Male Merged.jpg]

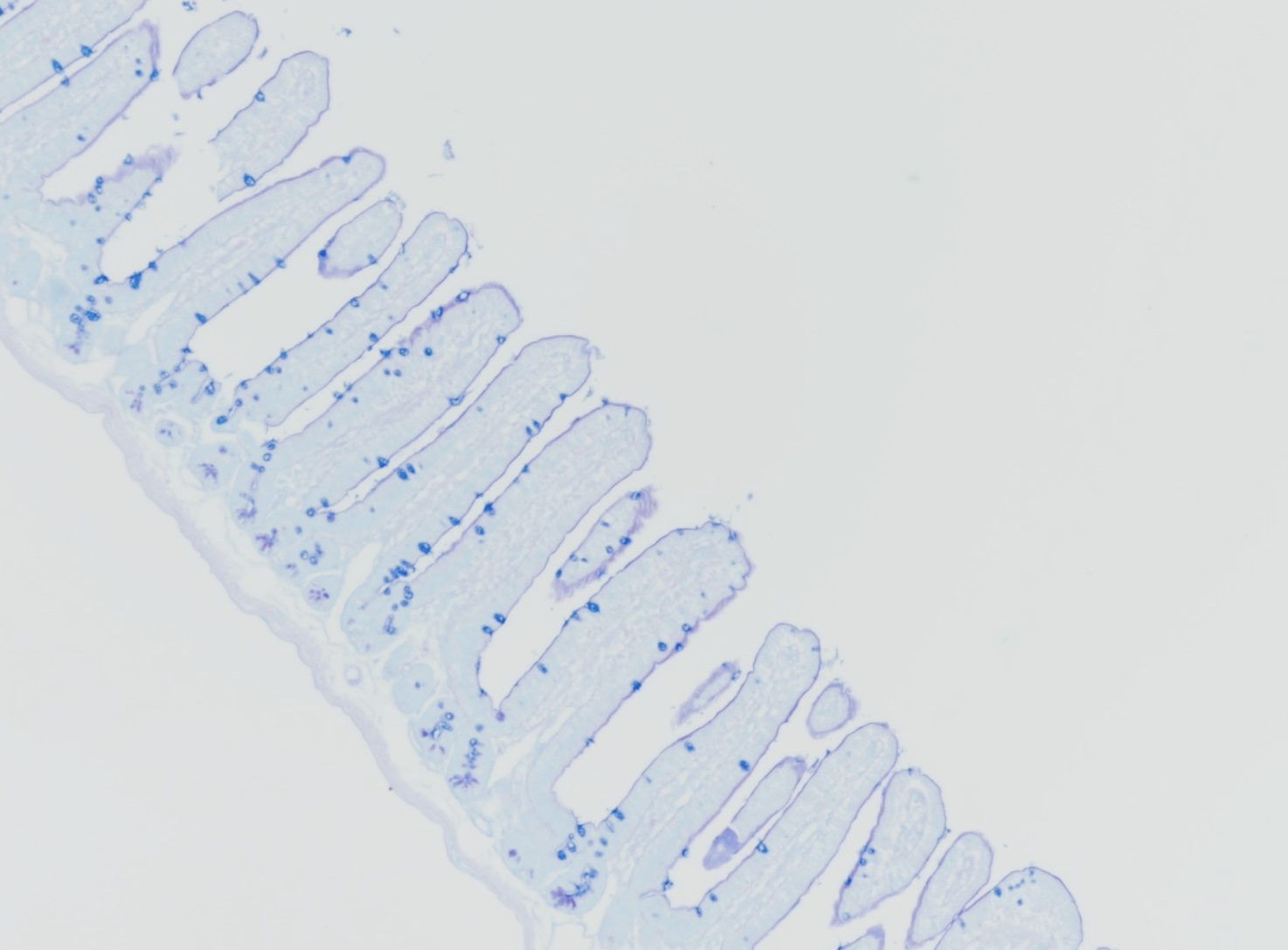

Supplement: Supplementary file 2 — Supporting File: advs75314‐sup‐0002‐RawData.zip. [file ADVS-13-e19337-s001.zip › Male mice.tif]

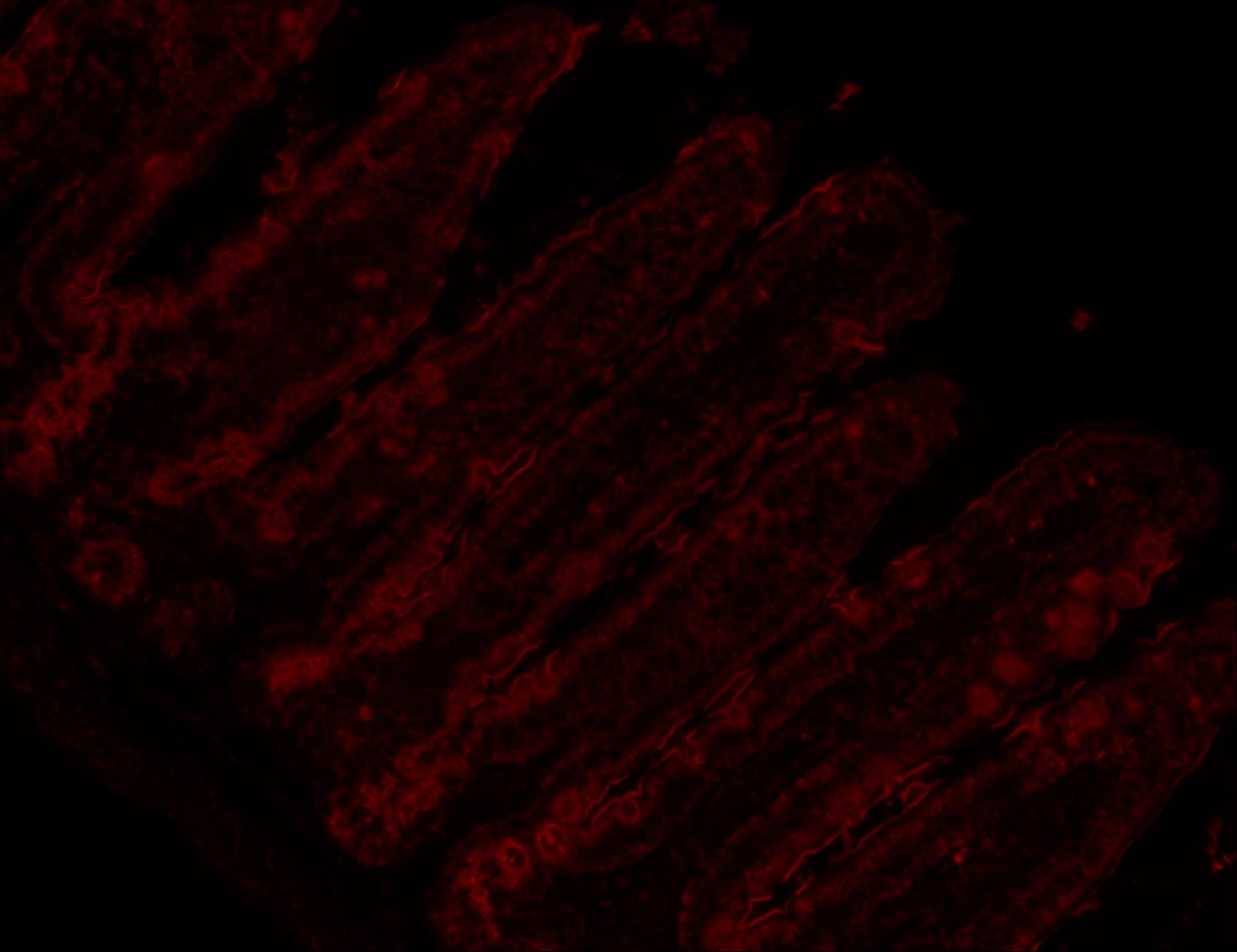

Supplement: Supplementary file 2 — Supporting File: advs75314‐sup‐0002‐RawData.zip. [file ADVS-13-e19337-s001.zip › Male MUC2.jpg]

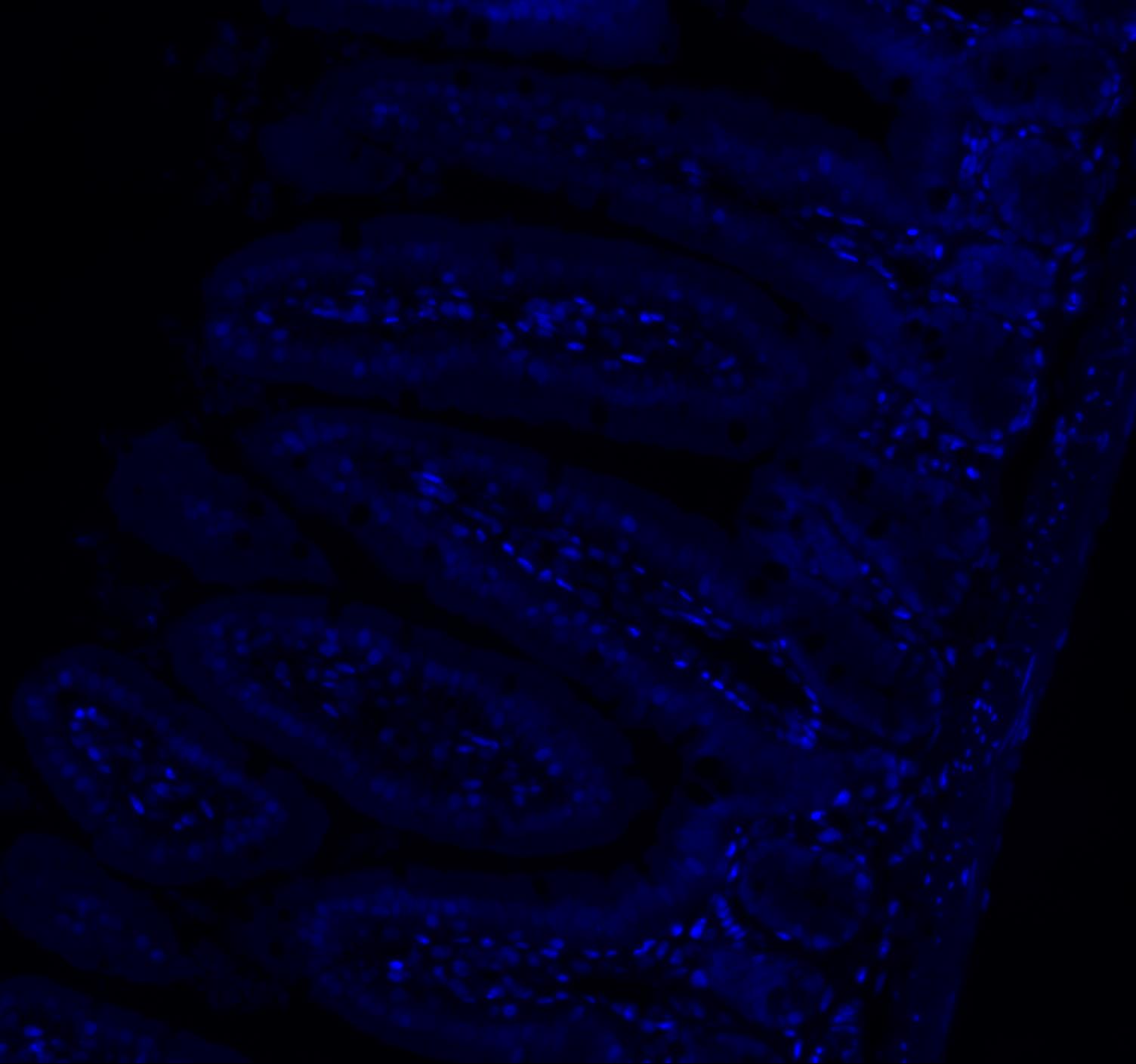

Supplement: Supplementary file 2 — Supporting File: advs75314‐sup‐0002‐RawData.zip. [file ADVS-13-e19337-s001.zip › Male+E2 DAPI.jpg]

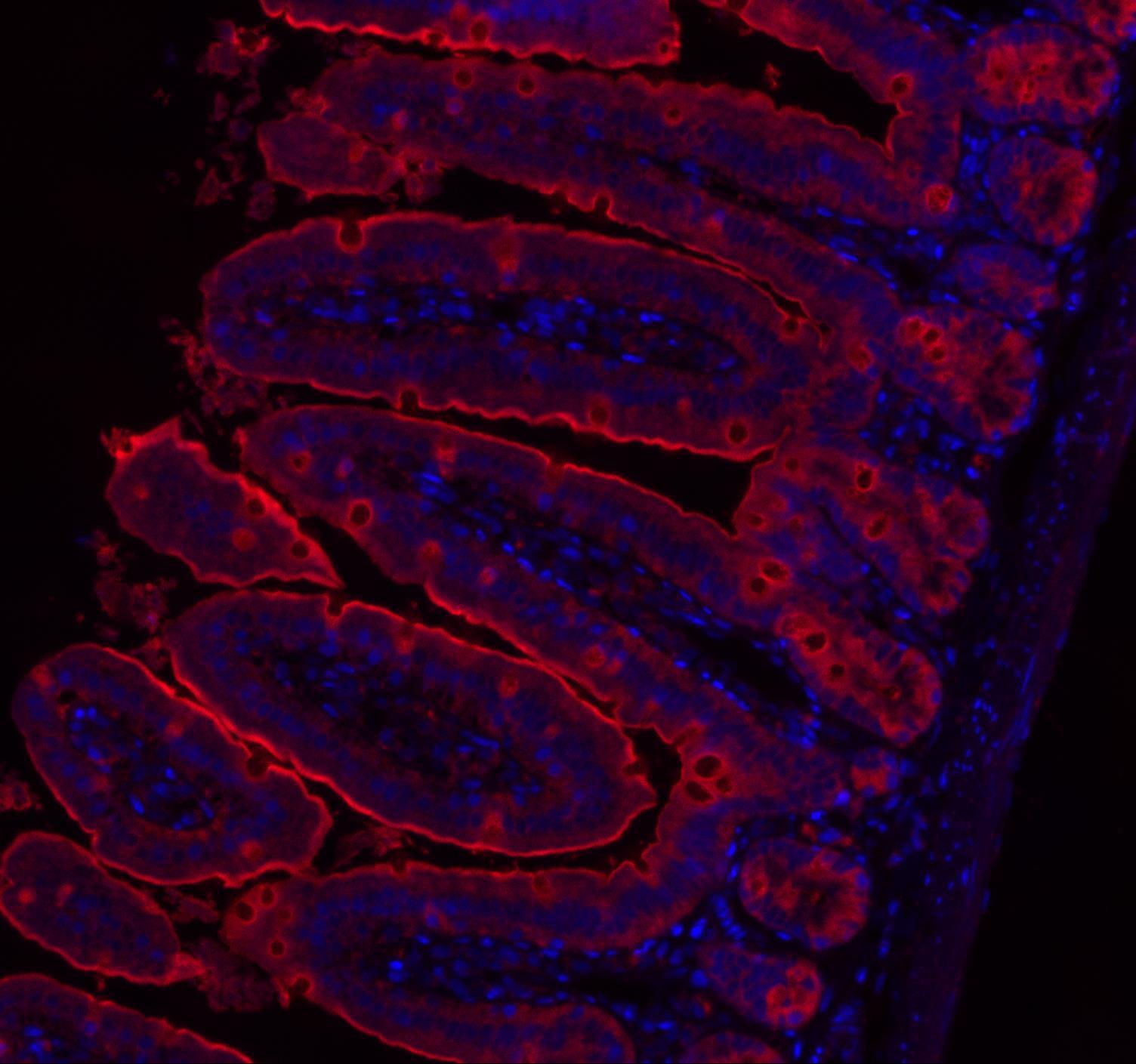

Supplement: Supplementary file 2 — Supporting File: advs75314‐sup‐0002‐RawData.zip. [file ADVS-13-e19337-s001.zip › Male+E2 Merged.jpg]

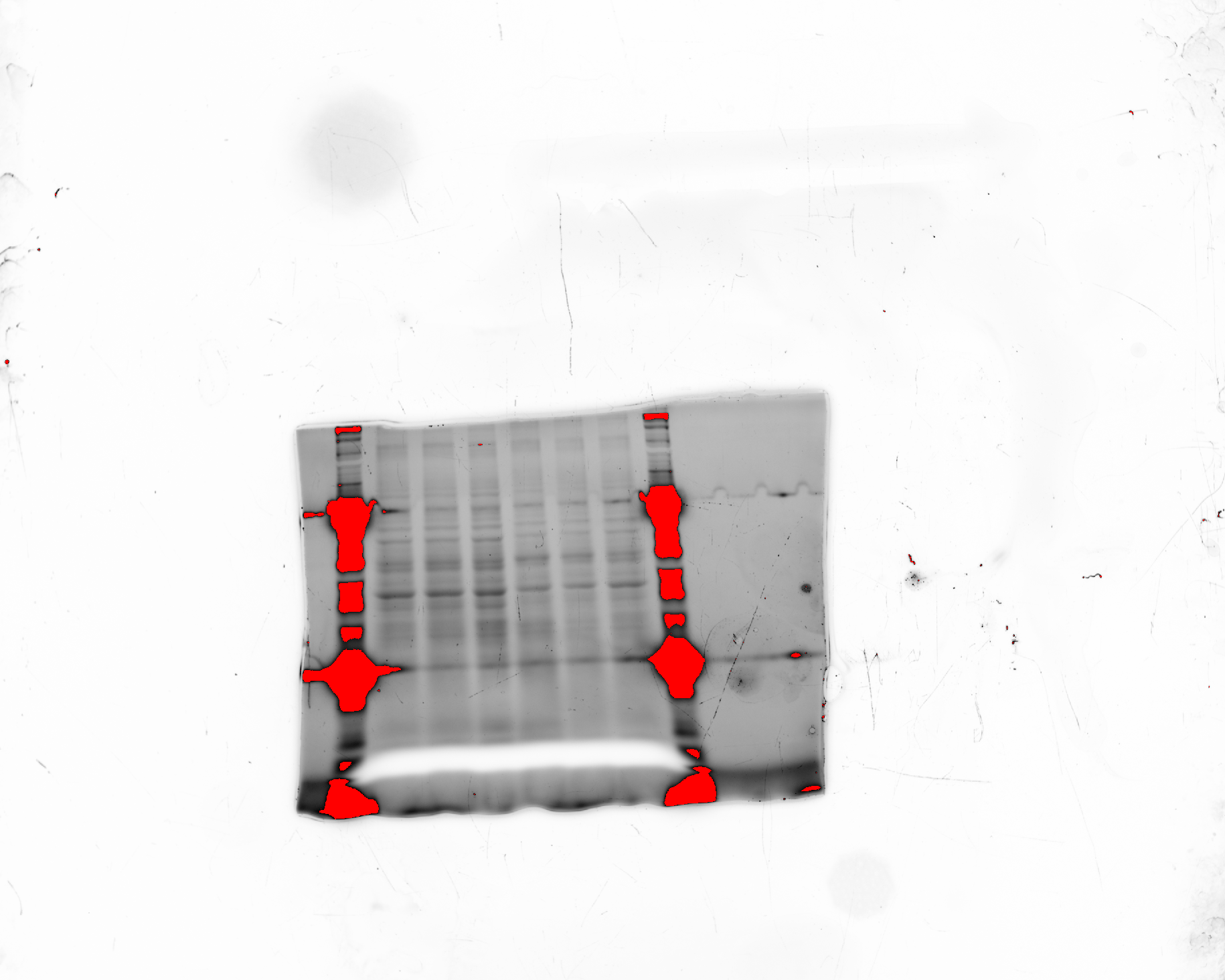

Supplement: Supplementary file 2 — Supporting File: advs75314‐sup‐0002‐RawData.zip. [file ADVS-13-e19337-s001.zip › Male+E2 mice jejunal fluid total protein(Stain Free Gel).tif]

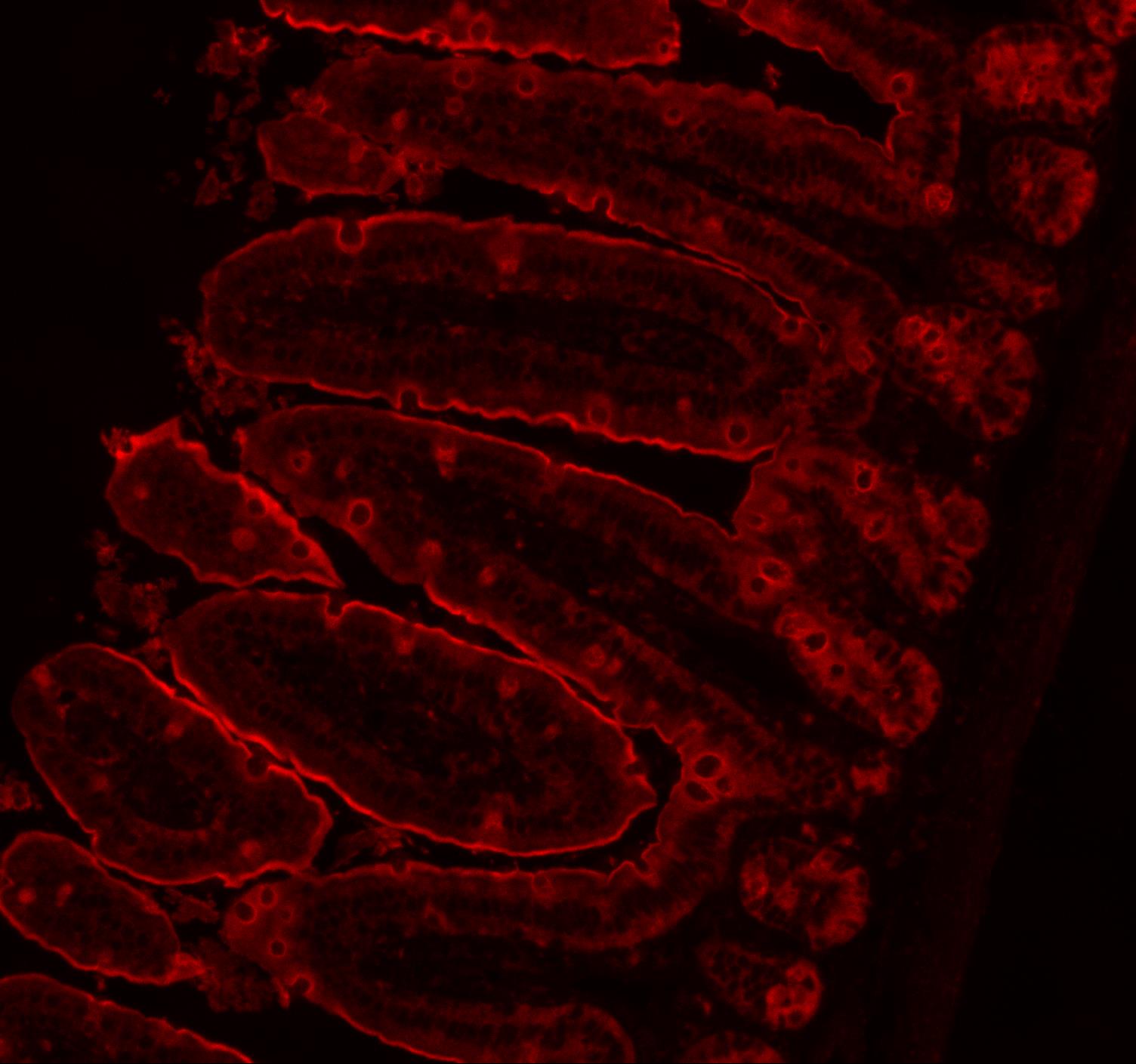

Supplement: Supplementary file 2 — Supporting File: advs75314‐sup‐0002‐RawData.zip. [file ADVS-13-e19337-s001.zip › Male+E2 MUC2.jpg]

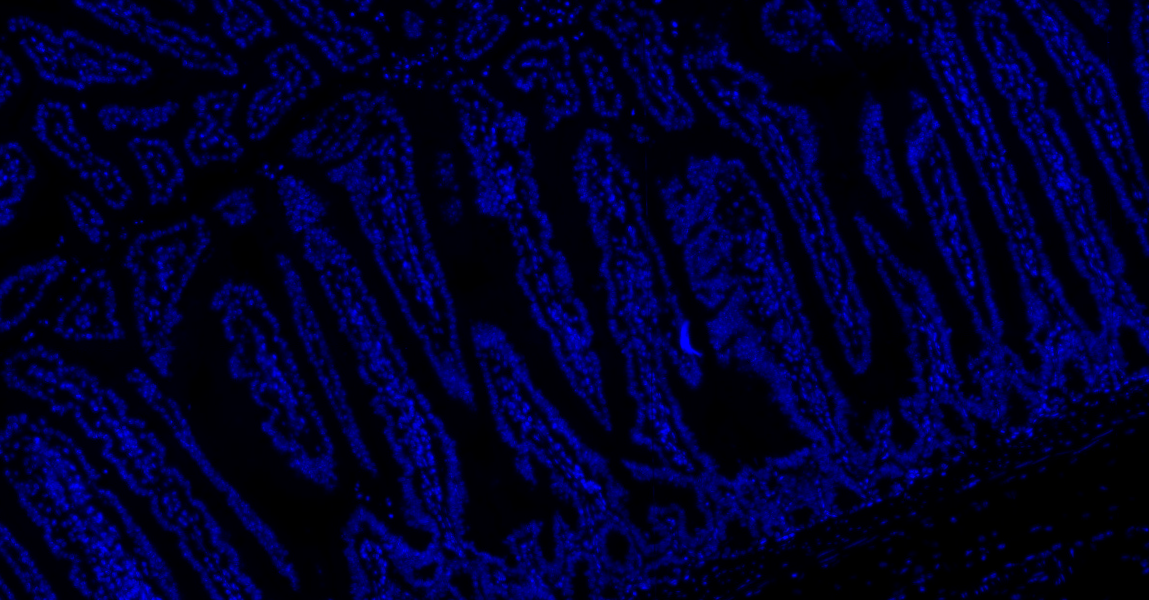

Supplement: Supplementary file 2 — Supporting File: advs75314‐sup‐0002‐RawData.zip. [file ADVS-13-e19337-s001.zip › Metestrus DAPI.tif]

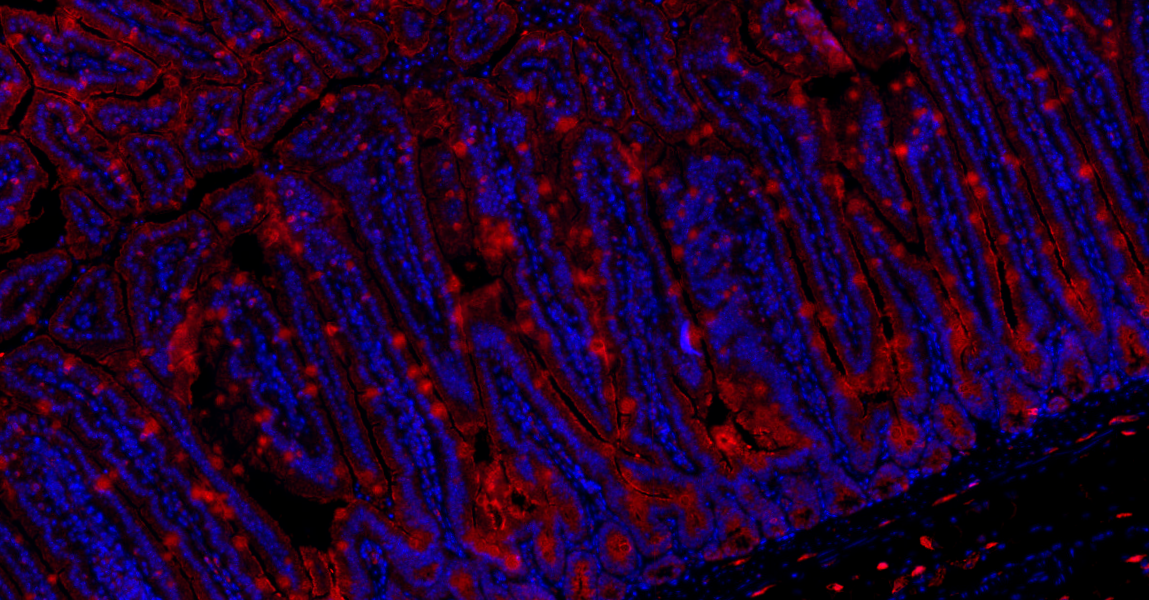

Supplement: Supplementary file 2 — Supporting File: advs75314‐sup‐0002‐RawData.zip. [file ADVS-13-e19337-s001.zip › Metestrus Merged.tif]

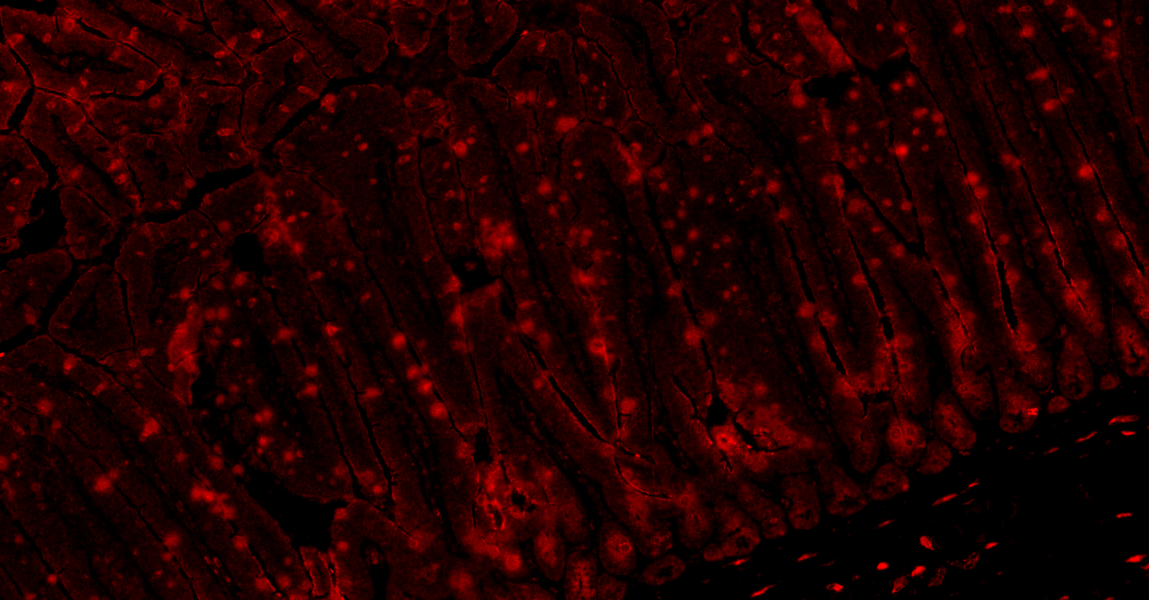

Supplement: Supplementary file 2 — Supporting File: advs75314‐sup‐0002‐RawData.zip. [file ADVS-13-e19337-s001.zip › Metestrus MUC2.tif]

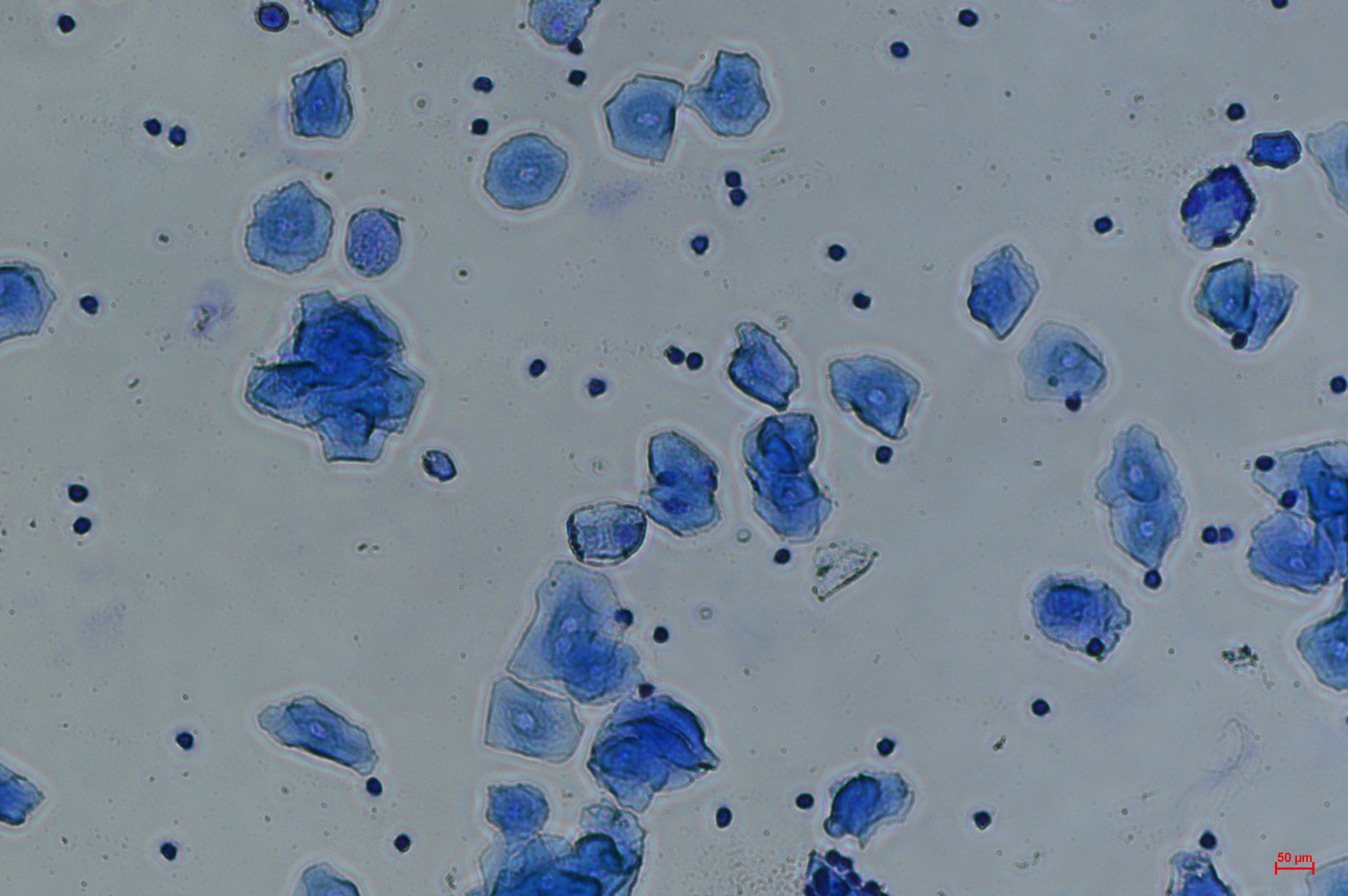

Supplement: Supplementary file 2 — Supporting File: advs75314‐sup‐0002‐RawData.zip. [file ADVS-13-e19337-s001.zip › Metestrus.tif]

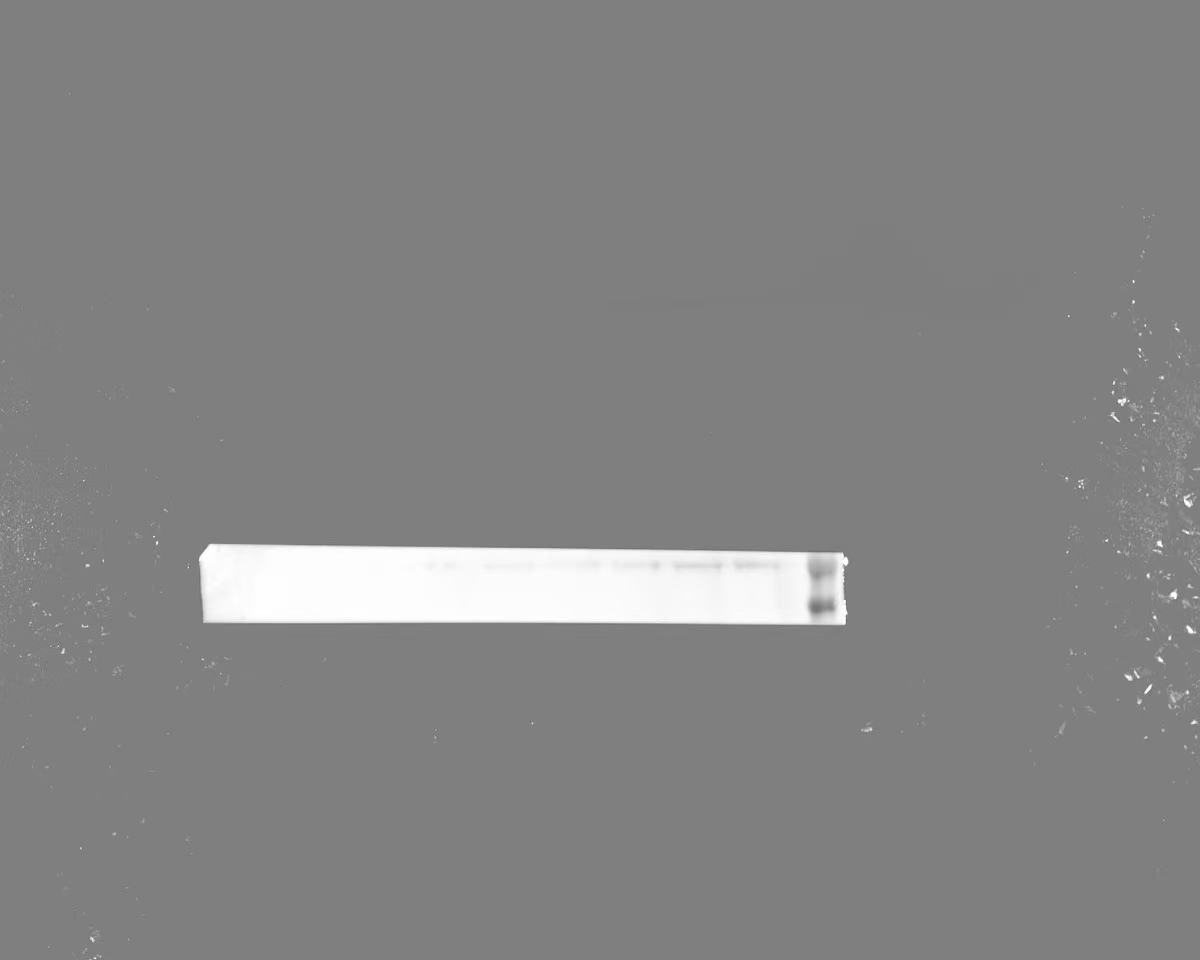

Supplement: Supplementary file 2 — Supporting File: advs75314‐sup‐0002‐RawData.zip. [file ADVS-13-e19337-s001.zip › MUC2 in the cell supernatants (CSNs)(Composite).tif]

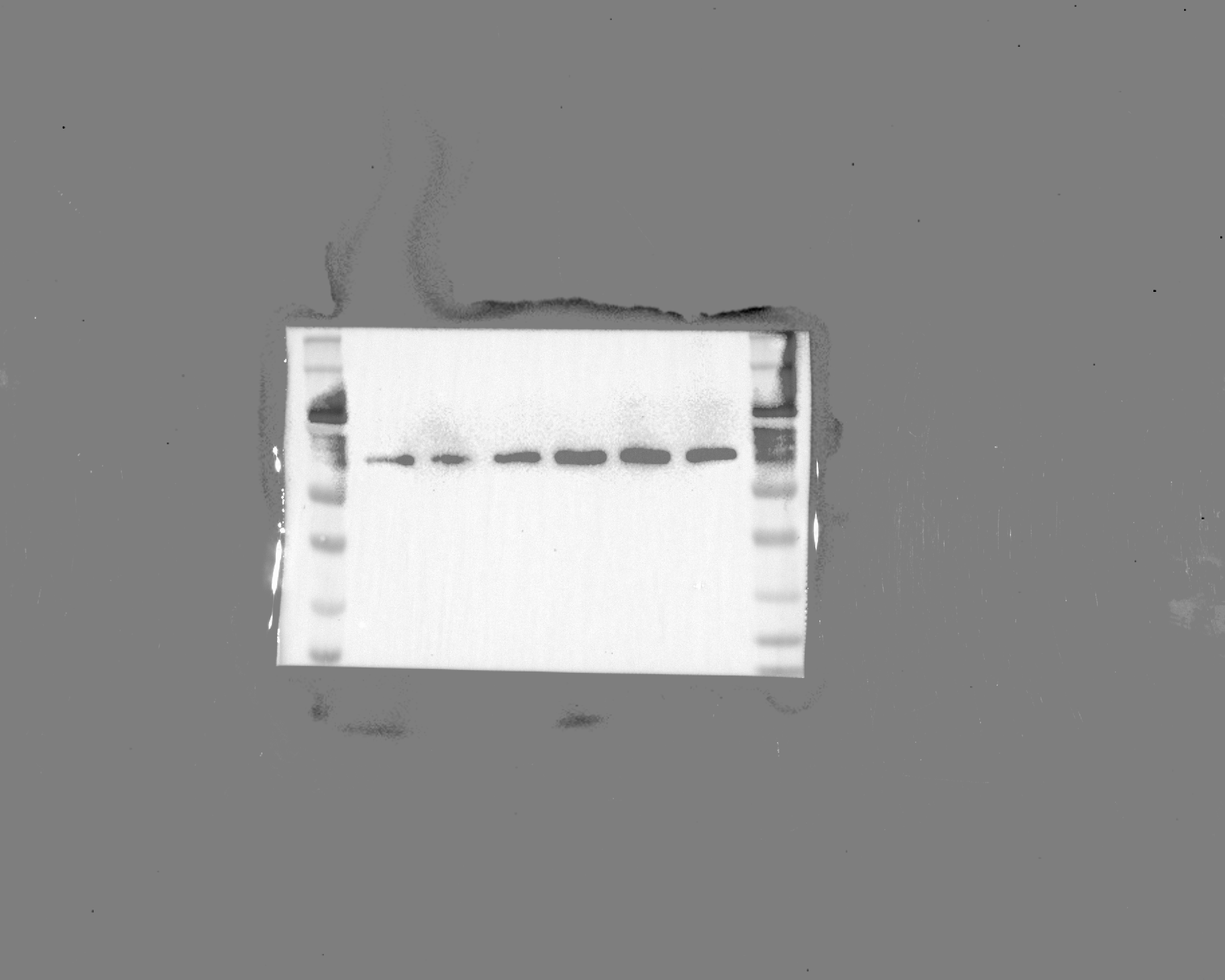

Supplement: Supplementary file 2 — Supporting File: advs75314‐sup‐0002‐RawData.zip. [file ADVS-13-e19337-s001.zip › Normal mice jejunal fluid muc2(Composite).tif]

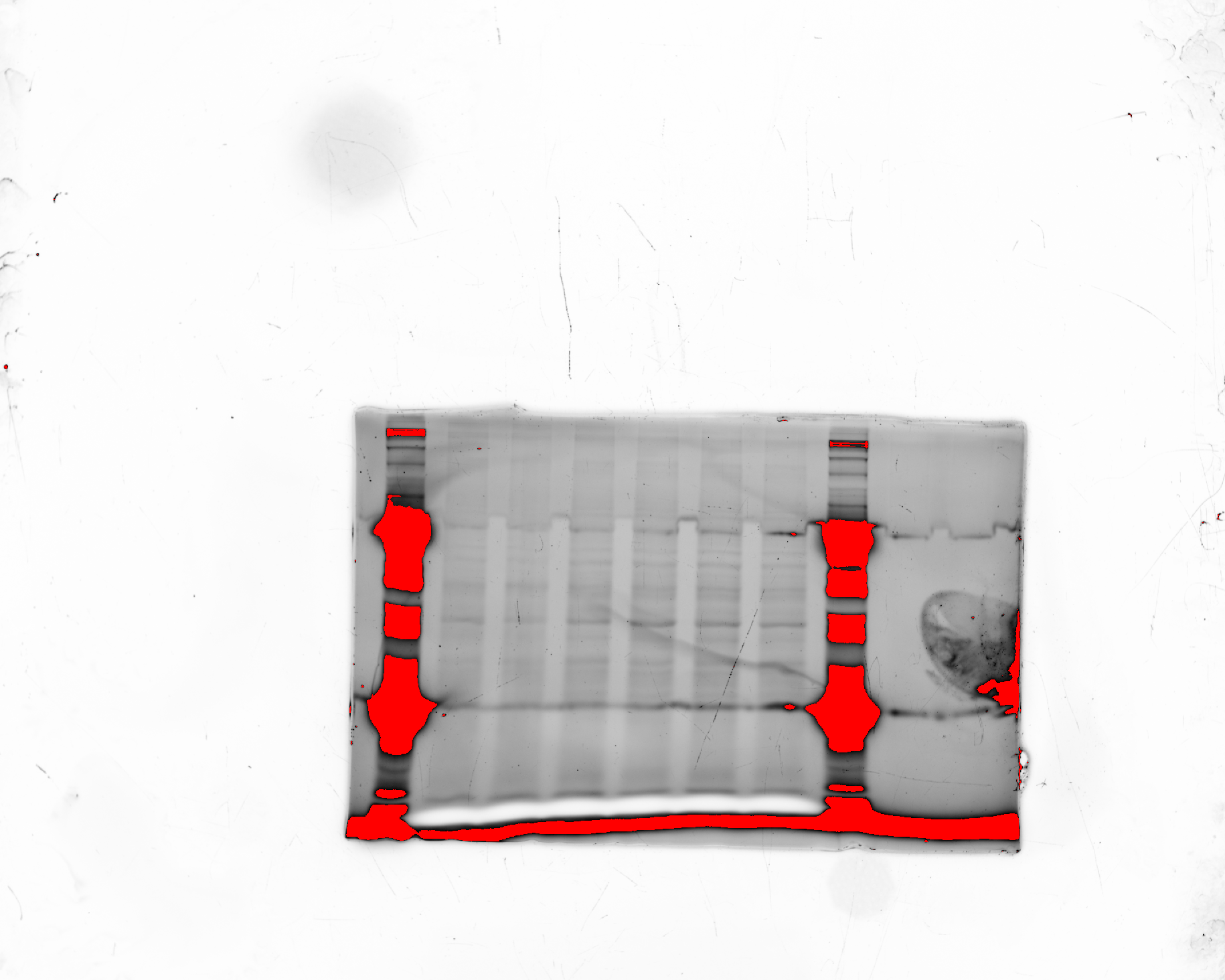

Supplement: Supplementary file 2 — Supporting File: advs75314‐sup‐0002‐RawData.zip. [file ADVS-13-e19337-s001.zip › Normal mice jejunal fluid total protein(Stain Free Gel).tif]

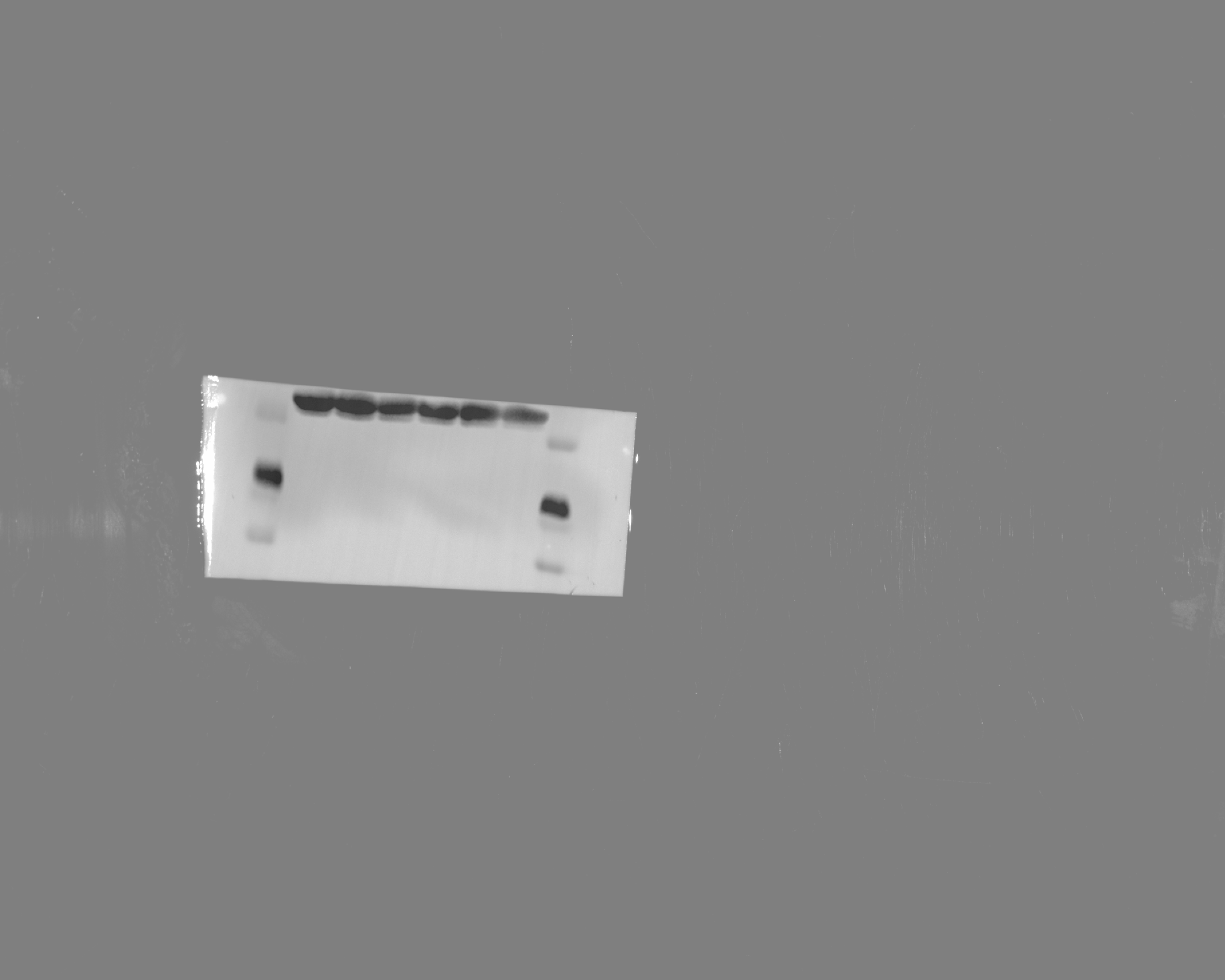

Supplement: Supplementary file 2 — Supporting File: advs75314‐sup‐0002‐RawData.zip. [file ADVS-13-e19337-s001.zip › Normal mice tissue actin(Composite).tif]

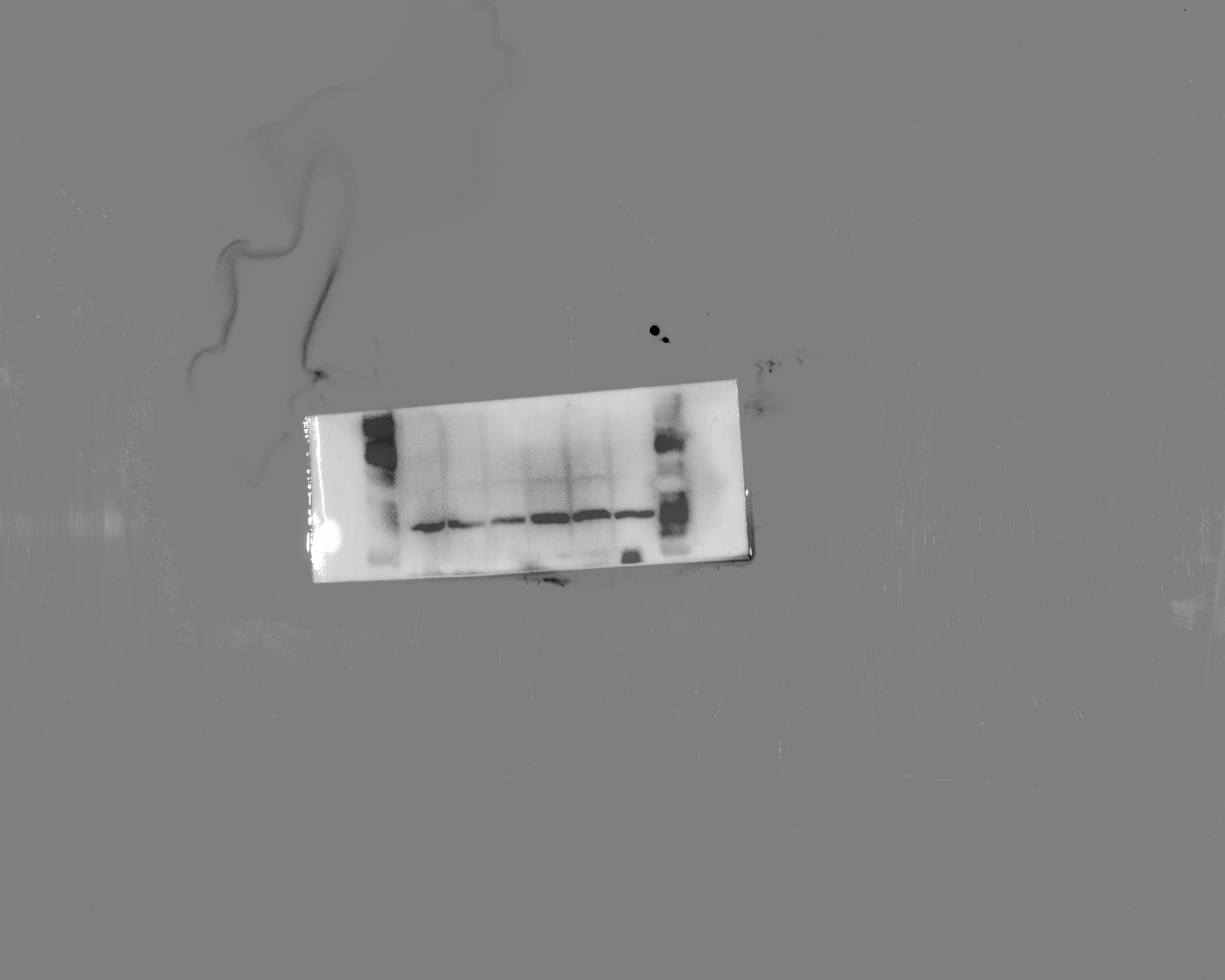

Supplement: Supplementary file 2 — Supporting File: advs75314‐sup‐0002‐RawData.zip. [file ADVS-13-e19337-s001.zip › Normal mice tissue muc2(Composite).tif]

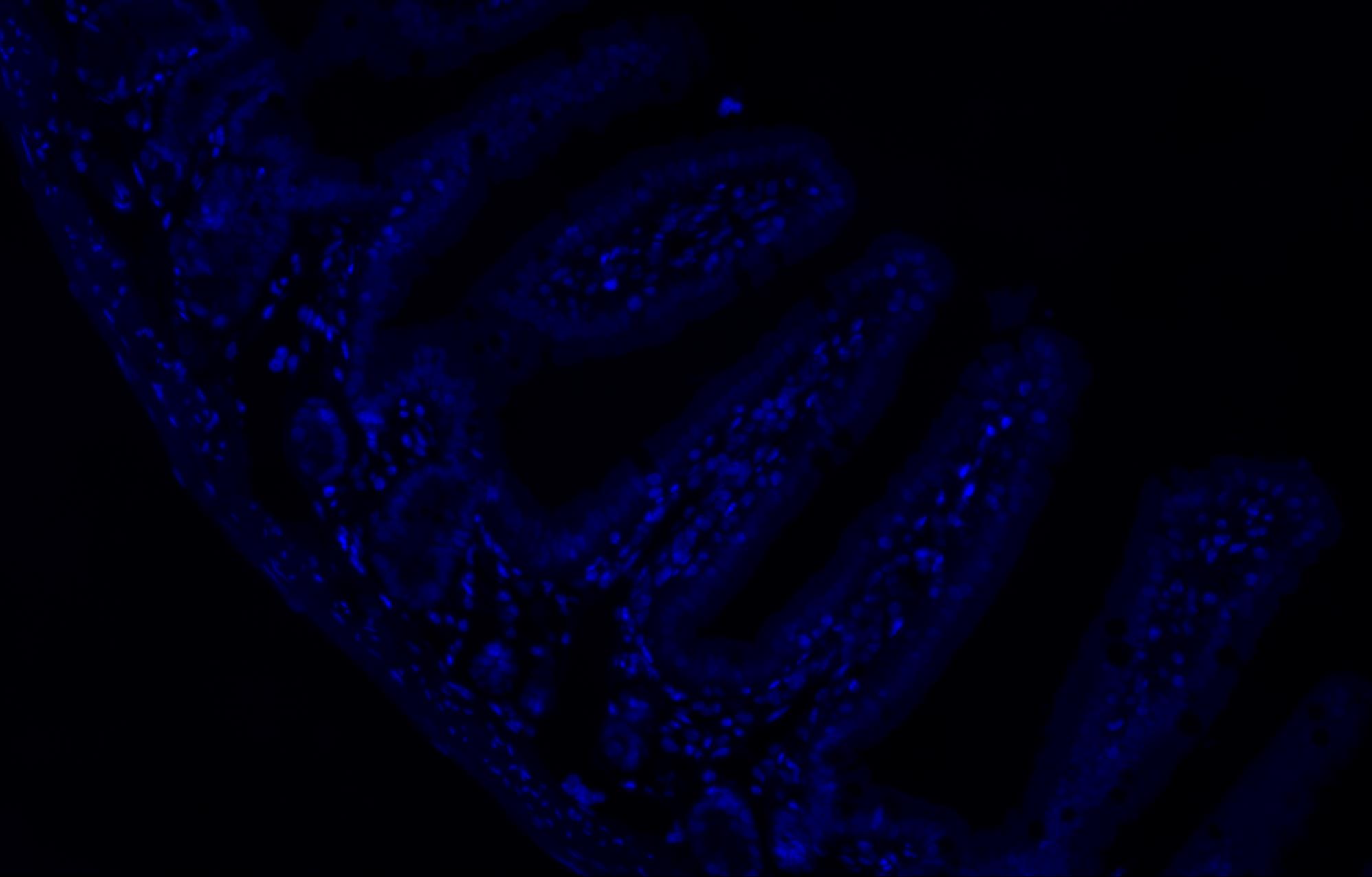

Supplement: Supplementary file 2 — Supporting File: advs75314‐sup‐0002‐RawData.zip. [file ADVS-13-e19337-s001.zip › OVX DAPI.tif]

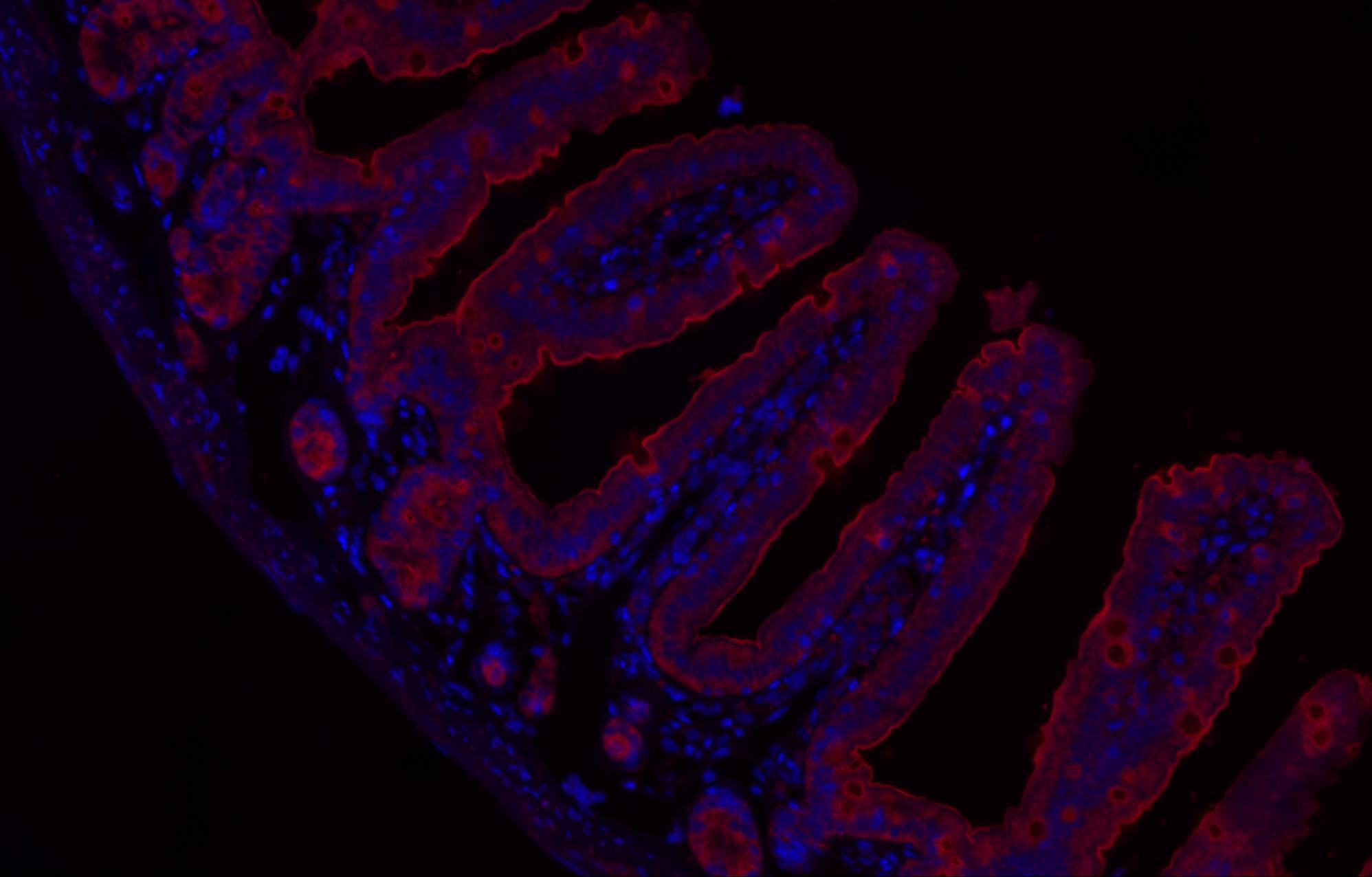

Supplement: Supplementary file 2 — Supporting File: advs75314‐sup‐0002‐RawData.zip. [file ADVS-13-e19337-s001.zip › OVX Merged.tif]

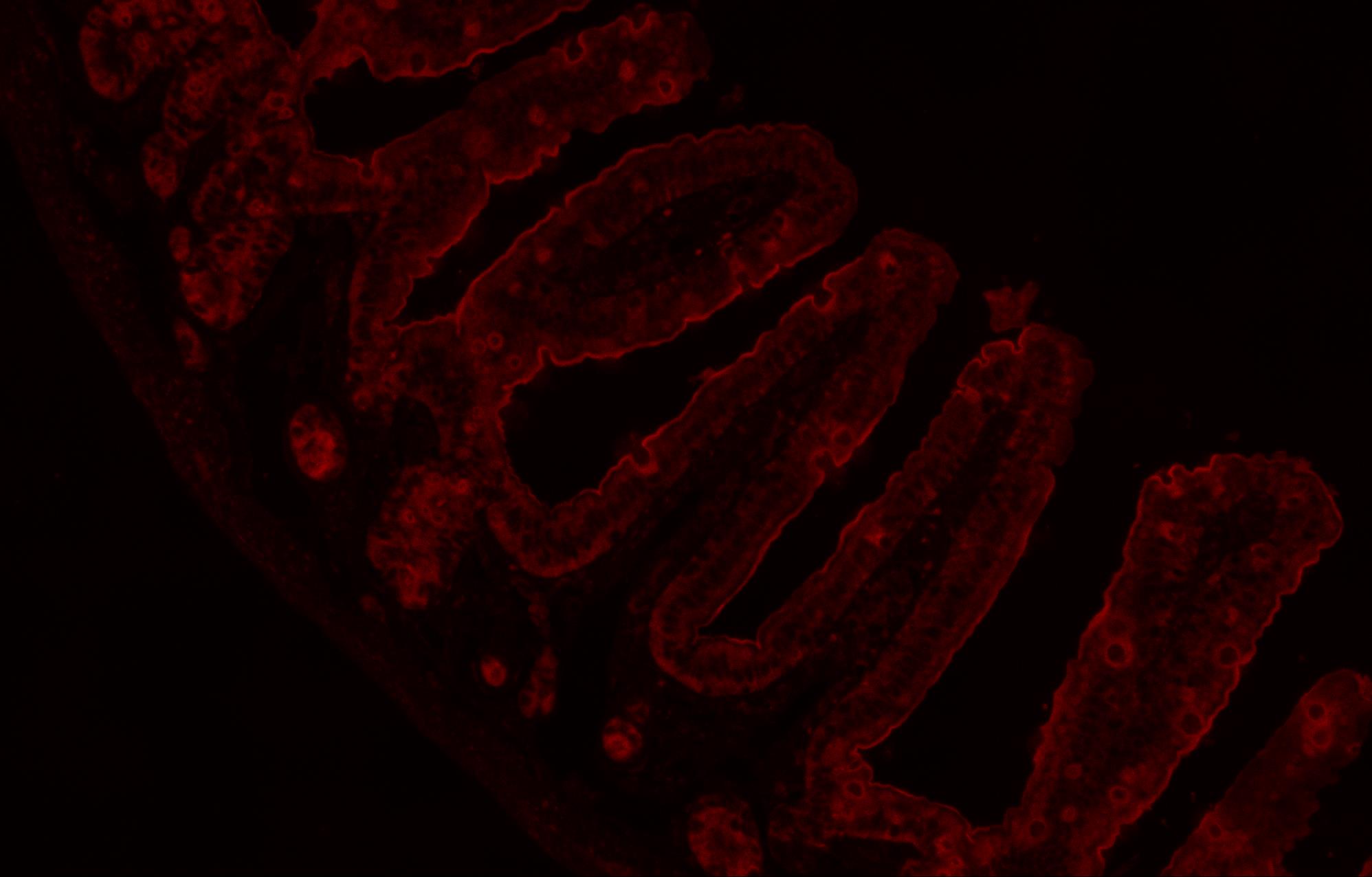

Supplement: Supplementary file 2 — Supporting File: advs75314‐sup‐0002‐RawData.zip. [file ADVS-13-e19337-s001.zip › OVX MUC2.tif]

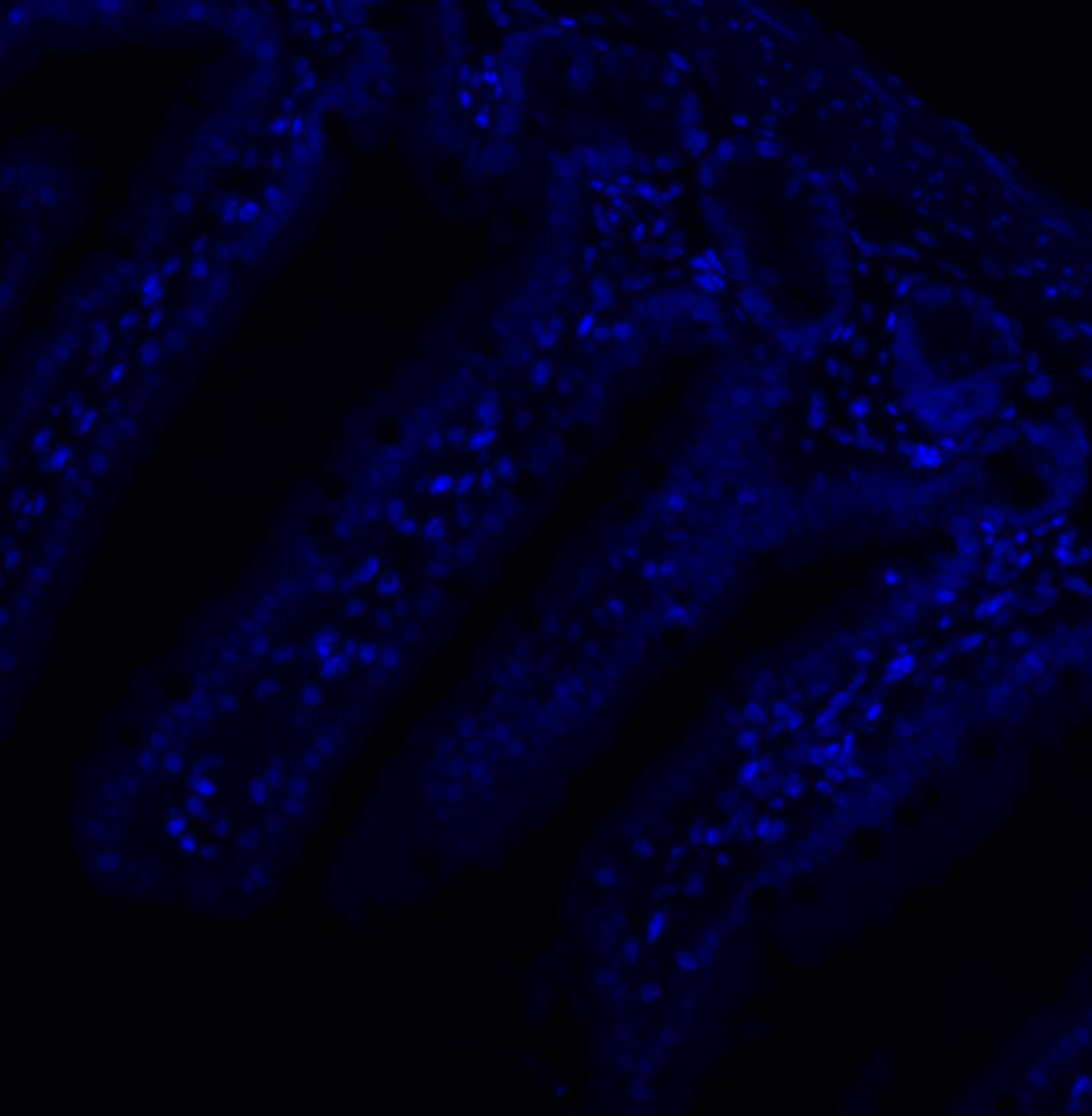

Supplement: Supplementary file 2 — Supporting File: advs75314‐sup‐0002‐RawData.zip. [file ADVS-13-e19337-s001.zip › OVX+E2 DAPI.tif]

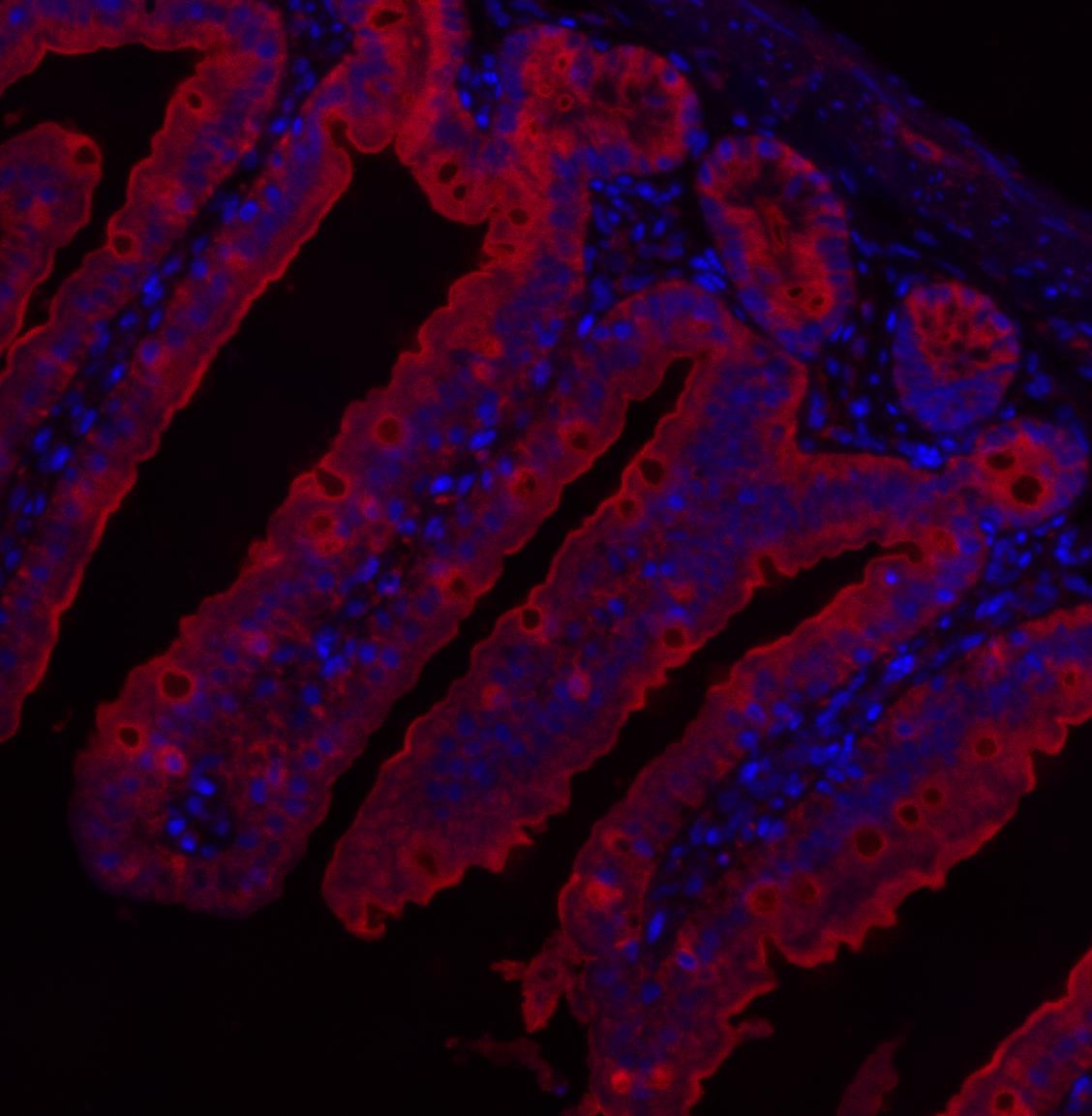

Supplement: Supplementary file 2 — Supporting File: advs75314‐sup‐0002‐RawData.zip. [file ADVS-13-e19337-s001.zip › OVX+E2 Merged.tif]

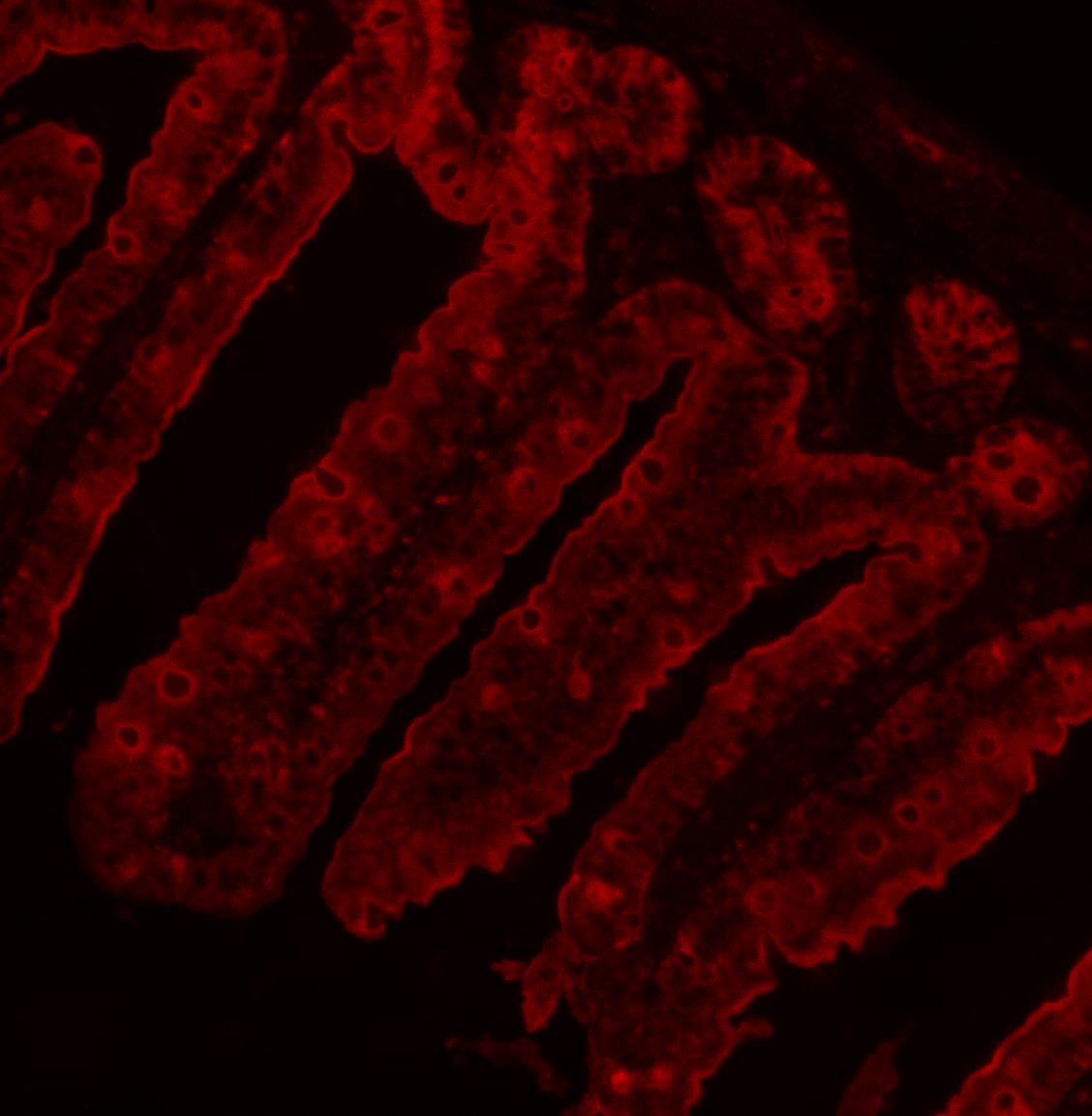

Supplement: Supplementary file 2 — Supporting File: advs75314‐sup‐0002‐RawData.zip. [file ADVS-13-e19337-s001.zip › OVX+E2 MUC2.tif]

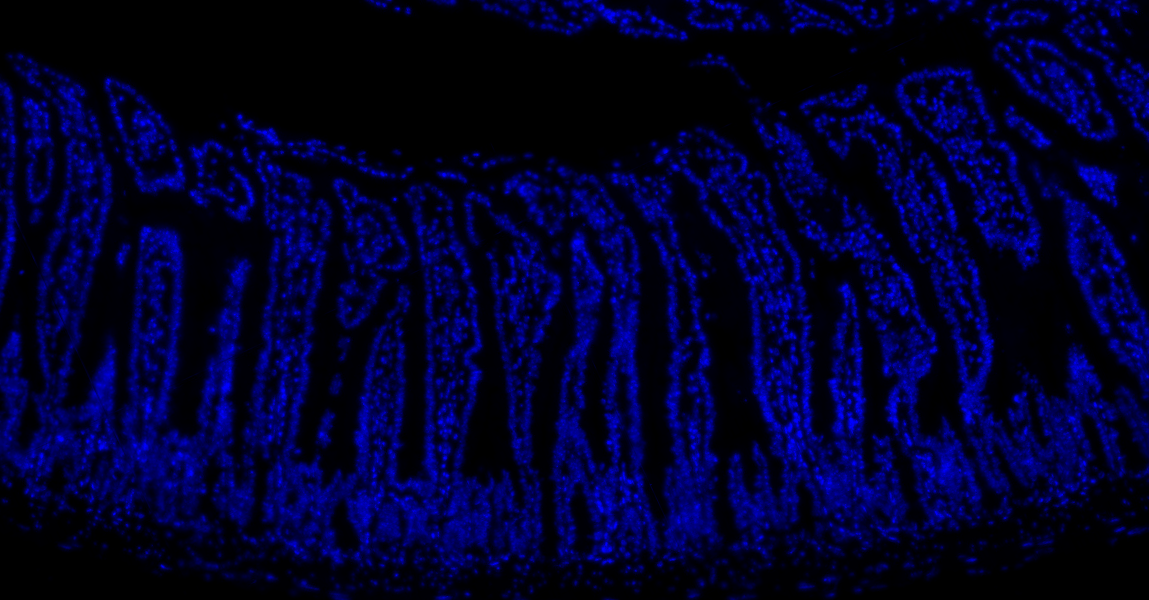

Supplement: Supplementary file 2 — Supporting File: advs75314‐sup‐0002‐RawData.zip. [file ADVS-13-e19337-s001.zip › Proestrus DAPI.tif]

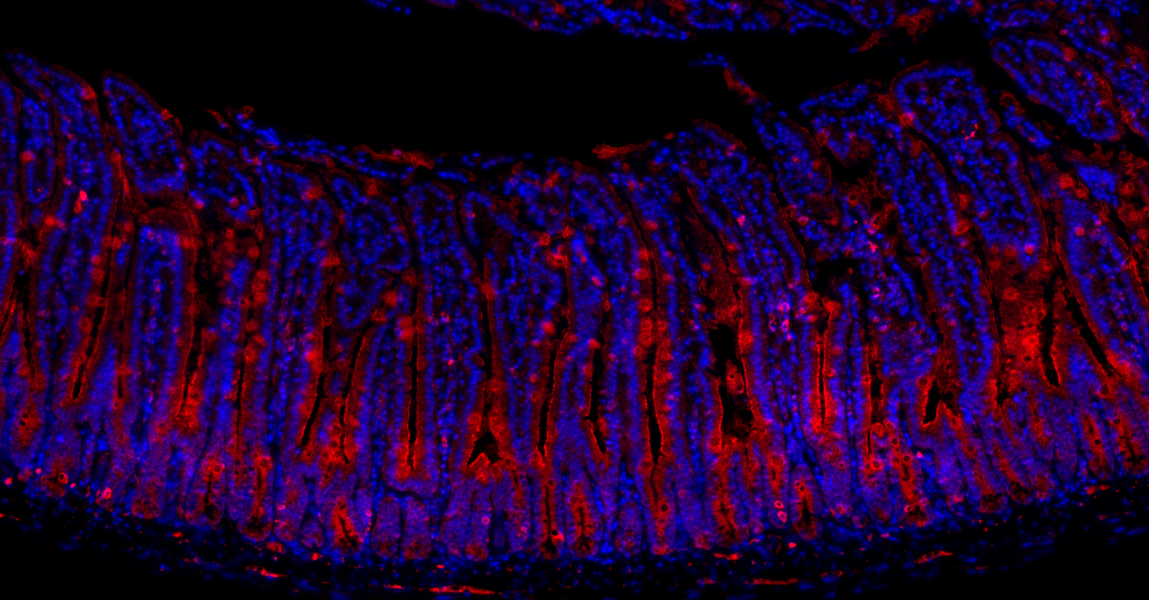

Supplement: Supplementary file 2 — Supporting File: advs75314‐sup‐0002‐RawData.zip. [file ADVS-13-e19337-s001.zip › Proestrus Mergedtif.tif]

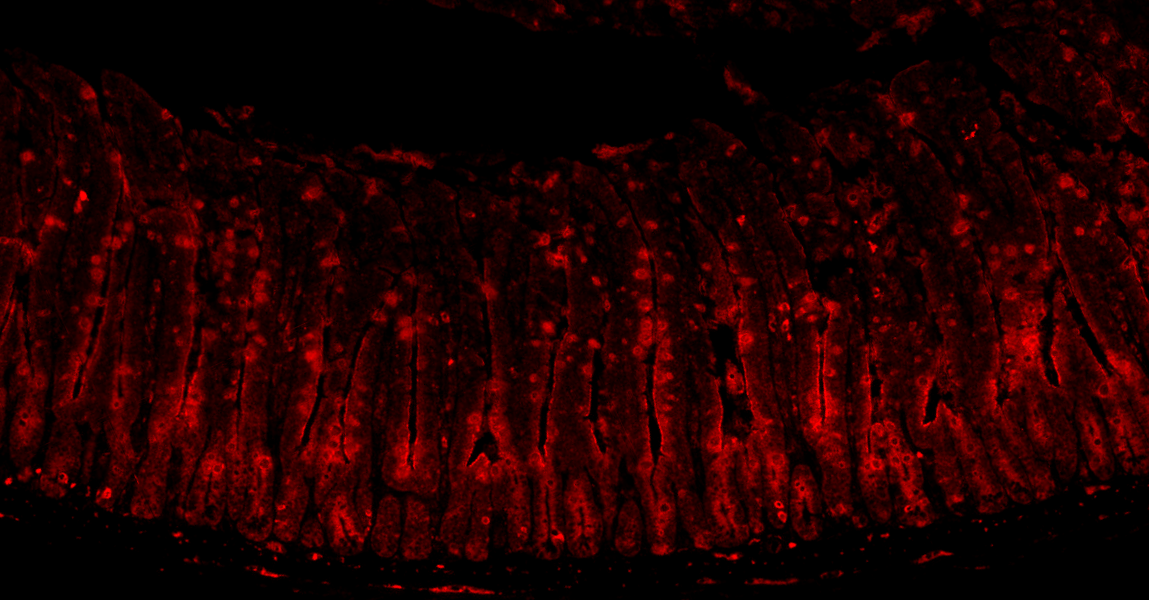

Supplement: Supplementary file 2 — Supporting File: advs75314‐sup‐0002‐RawData.zip. [file ADVS-13-e19337-s001.zip › Proestrus MUC2.tif]

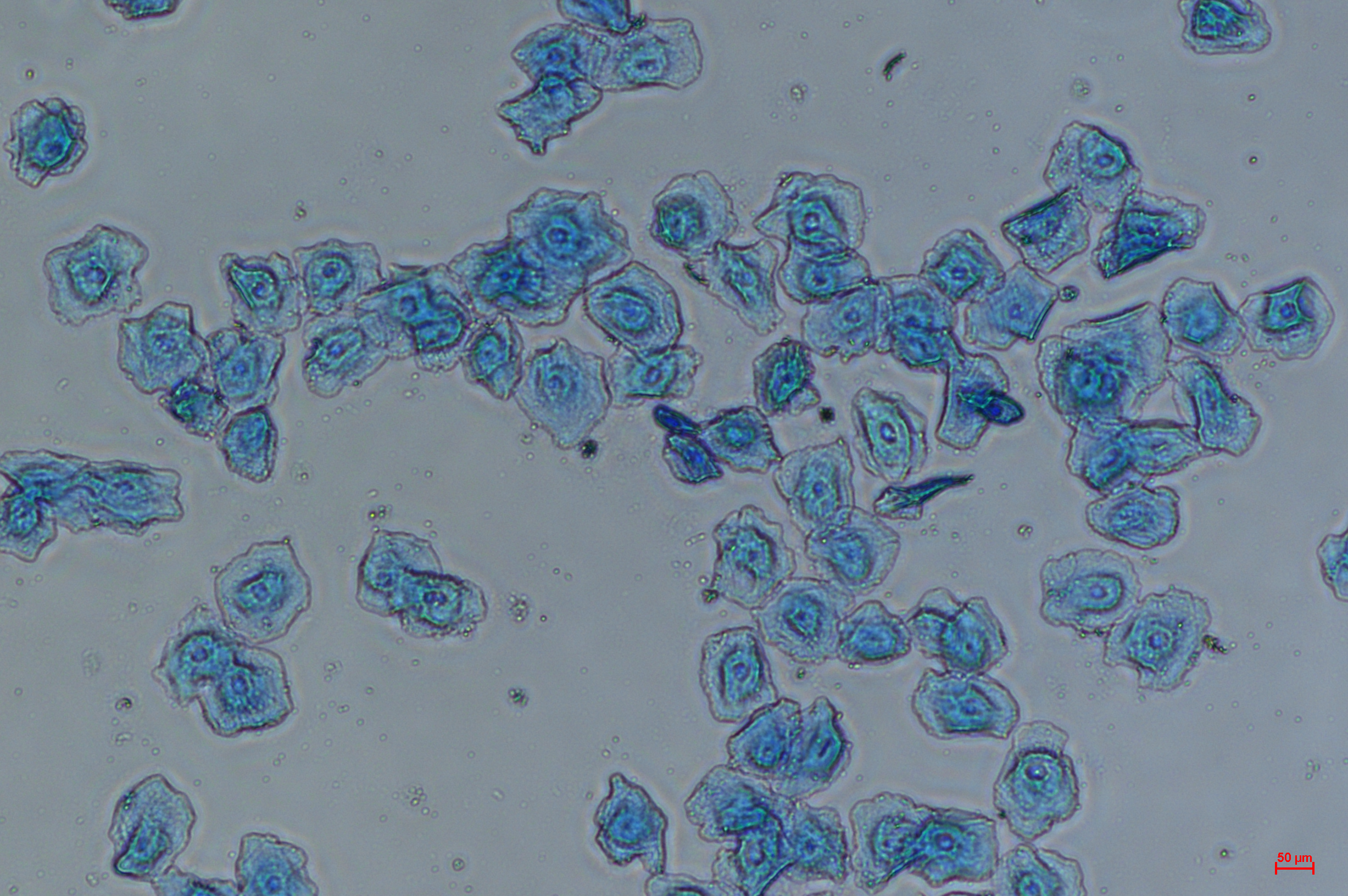

Supplement: Supplementary file 2 — Supporting File: advs75314‐sup‐0002‐RawData.zip. [file ADVS-13-e19337-s001.zip › Proestrus.tif]

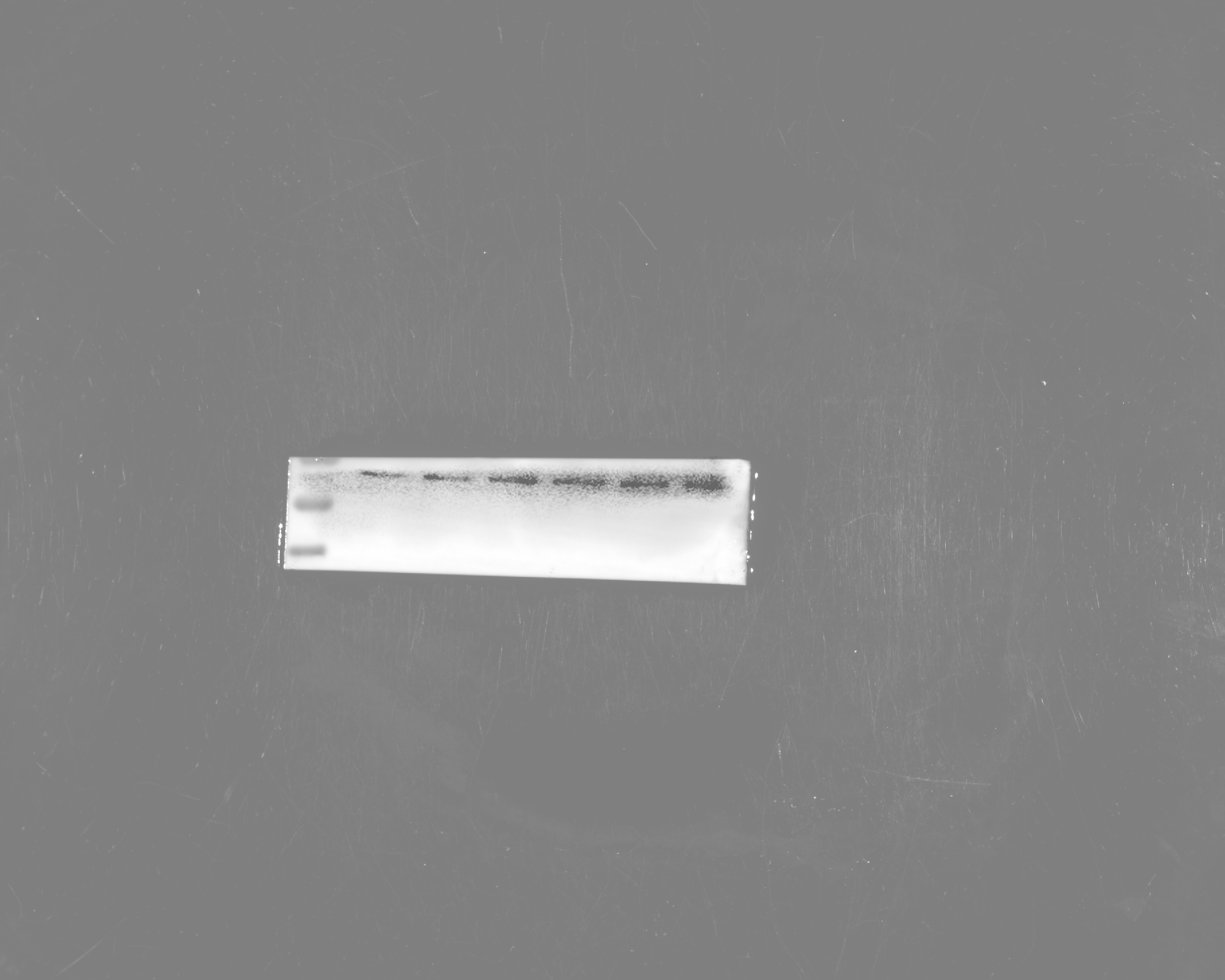

Supplement: Supplementary file 2 — Supporting File: advs75314‐sup‐0002‐RawData.zip. [file ADVS-13-e19337-s001.zip › serum HT-29 E2(Composite).tif]

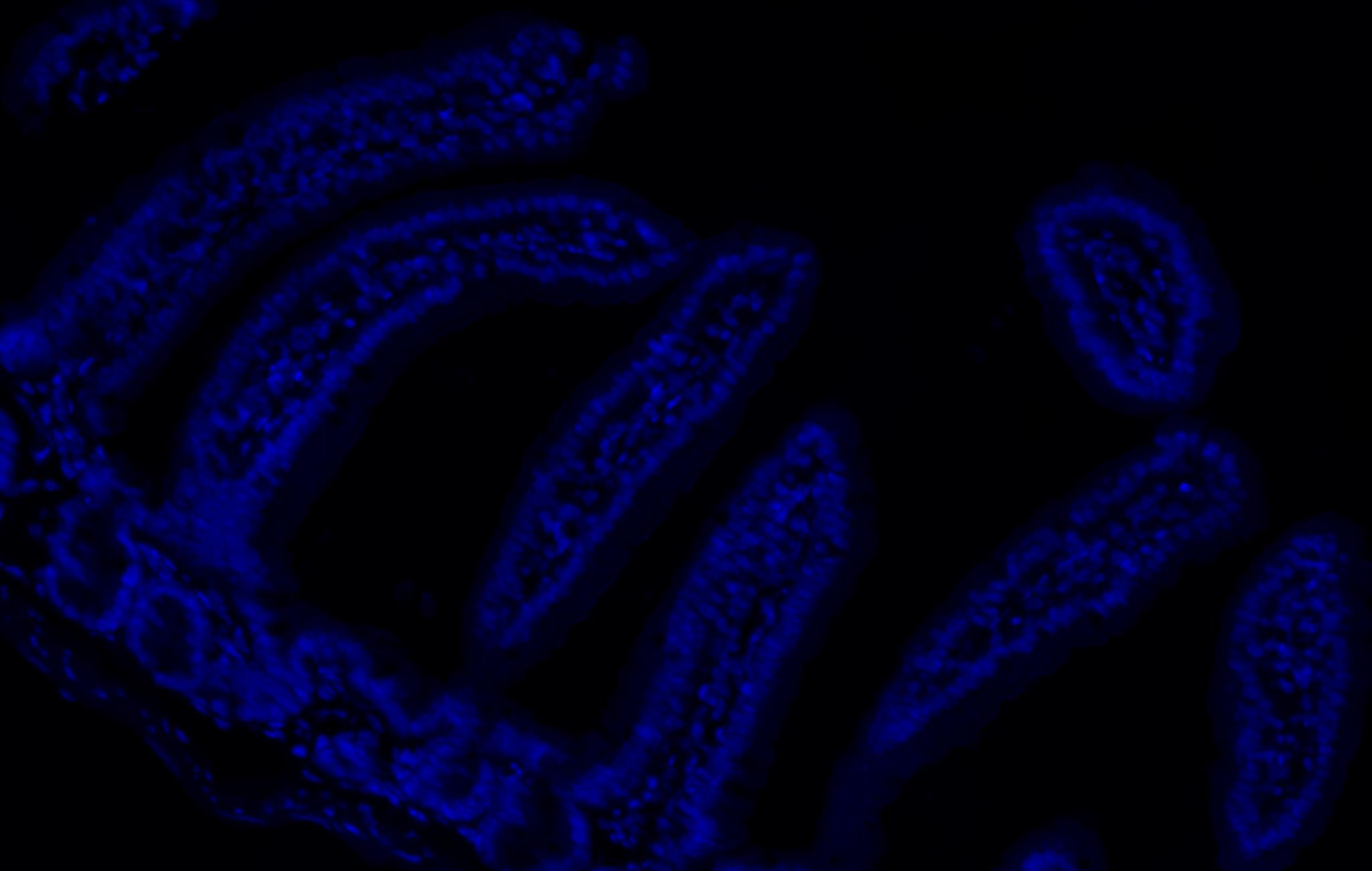

Supplement: Supplementary file 2 — Supporting File: advs75314‐sup‐0002‐RawData.zip. [file ADVS-13-e19337-s001.zip › Sham DAPI.tif]

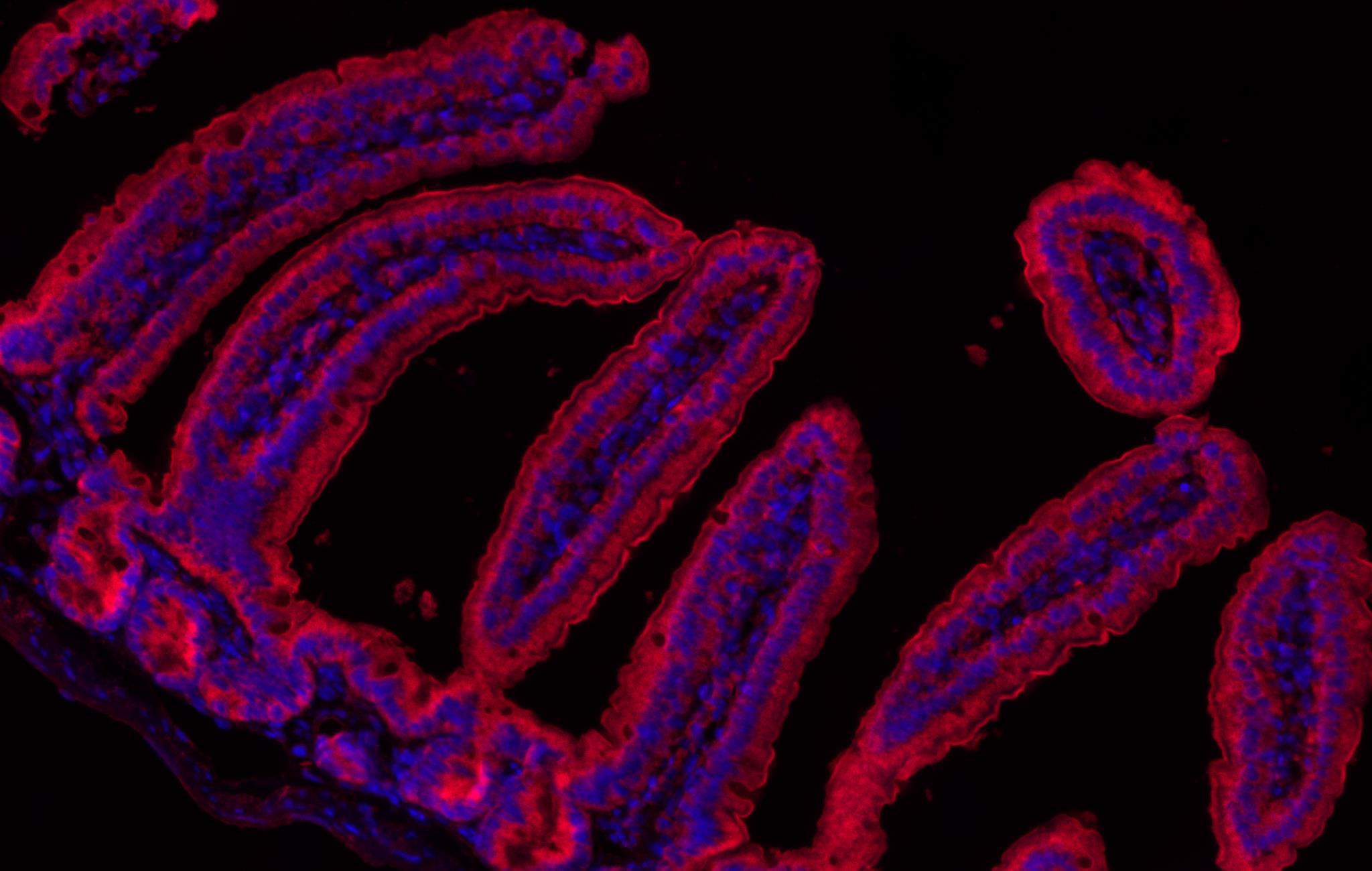

Supplement: Supplementary file 2 — Supporting File: advs75314‐sup‐0002‐RawData.zip. [file ADVS-13-e19337-s001.zip › Sham Merged.tif]

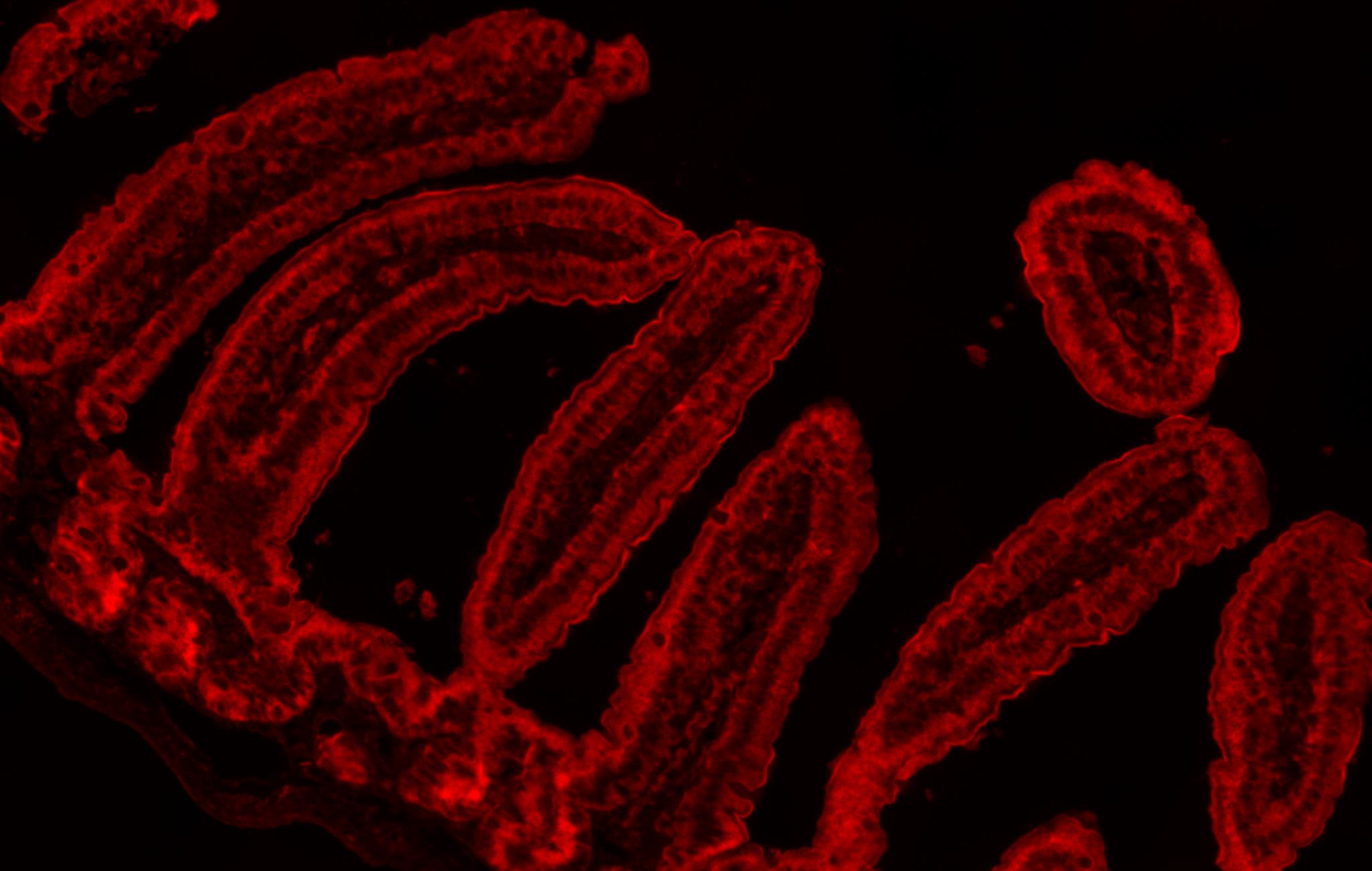

Supplement: Supplementary file 2 — Supporting File: advs75314‐sup‐0002‐RawData.zip. [file ADVS-13-e19337-s001.zip › Sham MUC2.tif]
